# Non-Cisplatin Concurrent Systemic Therapy with Radiotherapy for Locally Advanced Head and Neck Squamous Cell Carcinoma: A Network Meta-Analysis of Randomized Clinical Trials

**DOI:** 10.3390/cancers18101599

**Published:** 2026-05-14

**Authors:** Katharina Sophie Schöbel, Anne-Josephin Schoele, Georg Wurschi, Klaus Pietschmann, Maximilian Römer

**Affiliations:** 1 Department of Radiotherapy and Radiation Oncology, Jena University Hospital, 07747 Jena, Germany; anne-josephin.schoele@uni-jena.de (A.-J.S.); klaus.pietschmann@med.uni-jena.de (K.P.); 2 Comprehensive Cancer Center Central Germany, Partner Site Jena, 07747 Jena, Germany

**Keywords:** chemoradiotherapy, immunoradiotherapy, HNSCC, cisplatin-ineligible, second-line, cetuximab, carboplatin

## Abstract

**Background**: Head and neck squamous cell carcinoma (HNSCC) is among the top 10 most common cancer entities worldwide. For patients with locally advanced, non-metastatic HNSCC, the standard of care treatment often includes concomitant chemoradiotherapy with cisplatin. However, a considerable number of these patients are cisplatin-ineligible. To inform treatment selection for cisplatin-unfit patients undergoing radiotherapy (RT) for HNSCC, a network meta-analysis was performed. **Method**: In November 2023 a systematic search was conducted and updated in January 2026 searching four electronic databases (Medline, Web of Science, Cochrane Library, and Scopus) to identify randomized controlled trials (RCTs) analyzing overall survival (OS), progression-free survival (PFS), locoregional control (LC) and adverse events (AE) of RT combined with chemo-/immunotherapy with relevance to treatment selection in cisplatin-ineligible patients with HNSCC. **Results:** From all (15,553) search results, 52 publications concerning 28 RCTs reporting on approximately 7000 patients were included. The therapeutic concepts in the experimental group included all combinations of RT with concurrent systemic therapy, excluding cisplatin-based regimens, and were compared with any control treatment. All studies had sufficient quality for inclusion. None of the systemic agents showed a significant improvement in OS, PFS, or LC compared to cisplatin. Carboplatin + 5-FU and mitomycin C + 5-FU improved LC compared to hyperfractionated accelerated radiotherapy (HART). Compared to cisplatin, durvalumab and cetuximab were associated with less dysphagia, cetuximab with less renal impairment, and panitumumab and cetuximab with less weight loss. **Conclusions:** Carboplatin + 5-FU and Mitomycin C + 5-FU achieve superior LC compared to HART. Targeted therapy and immunotherapy were associated with less severe AEs.

## Simple Summary

Head and neck squamous cell carcinoma is one of the most common cancer types worldwide. The standard therapy for patients with locally advanced disease that has not spread to distant organs typically includes radiotherapy combined with cisplatin chemotherapy. However, a considerable number of patients are not fit for this regimen, due to poor general health or because of an increased risk for severe side effects like kidney and hearing damage. For this group, alternative treatment options are needed. Our work aims to compare different cisplatin-free regimens, including data from 28 studies with 7000 patients. Compared to cisplatin, none of the alternatives showed superior length of survival or tumor control. However, durvalumab and cetuximab induce less dysphagia, cetuximab induces less renal impairment, and panitumumab and cetuximab induce less weight loss than cisplatin. Our findings may help to guide treatment decisions for patients who cannot receive cisplatin.

## 1. Introduction

With 750,000 new cases worldwide annually, HNSCC is among the most common cancer entities [1]. Men are affected about twice as often as women, and numerous risk factors, such as smoking, alcohol consumption, and HPV infection, favor their development [2]. Accordingly, a therapy that not only achieves a high overall survival rate but also preserves the patients’ quality of life during and after treatment is important. In a curatively intended setting, surgical resection and chemoradiotherapy (CRT) represent the mainstay treatment options. However, feasible therapies vary depending on the stage of the disease. For locally advanced HNSCC, the preferred treatment is surgical resection followed by adjuvant CRT [3]. If the tumor is functionally unresectable or contraindications for surgery exist, definitive CRT is the treatment of choice as a curative-intended approach. Owing to its ability to improve regional control rates and overall survival, cisplatin is the concurrent agent of choice [3]. In addition to known hypersensitivity to the drug, various contraindications exist for its use, including impaired renal function, pre-existing hearing impairment, as well as myelosuppression and peripheral neuropathy [4]. However, no universally standardized definition for cisplatin-ineligibility exists, limiting research in this specific subgroup, and therefore recommendations vary. According to current guidelines [5], carboplatin, cetuximab, and taxane-based treatments are commonly used options for concurrent therapy in this scenario. However, selecting the regimen of choice is challenging, as they differ substantially in terms of reported side-effect profiles and oncologic efficacy [3]. Carboplatin was the first platinum preparation to be developed for better tolerability and features a modified side effect profile with lower nephrotoxicity and emetogenicity compared to cisplatin [6]. Taxanes use a different mechanism of action by inducing the polymerization of microtubules and thus inhibiting cell division [7]. The EGFR antibody cetuximab is a more recent addition to combination regimens. Due to its higher affinity to the epidermal growth factor receptor, it can displace the body’s own ligands and thus negatively regulate cell growth by internalizing the receptor, resulting in inhibition of tumor growth [8]. The heterogeneity of treatment options, particularly combinations of two or more of these drugs, represents an enormous challenge in the clinical setting. This network meta-analysis (NMA) compares cisplatin-free treatment schedules in order to simplify the choice of treatment for cisplatin-ineligible patients.

## 2. Method

### 2.1. Protocol and Study Selection

This systematic review and NMA were conducted in accordance with the 2020 Preferred Reporting Items for Systematic Reviews and Meta-Analyses (PRISMA) guidelines. The review was prospectively registered on PROSPERO (CRD42024578276). The final protocol is provided in the Supplementary Materials (Supplementary Material S1).

A systematic search was initially conducted in four databases (Medline (PubMed), Web of Science Core Collection (WoS), Cochrane Library, and Scopus) from November 2023 to January 2026. For each database, a complex search strategy was developed consisting of a combination of MeSH terms, keywords, and synonyms, including different spelling variants connected to HNSCC and chemoradiotherapy or immunoradiotherapy. The complete search string is available in the Supplementary Materials (Supplementary Material S2).

**Figure 1:**
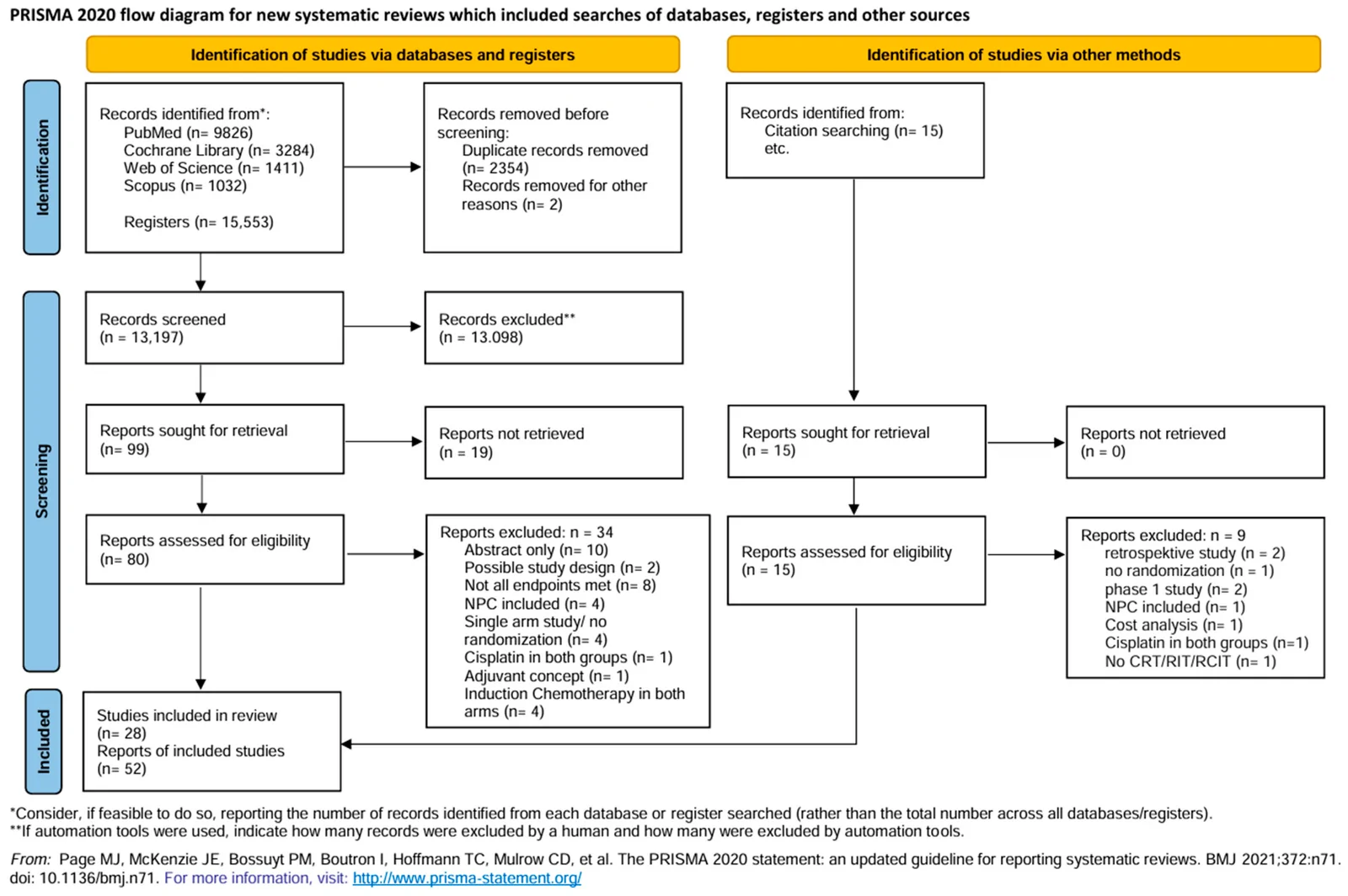
PRISMA flowchart [9]. PRISMA checklist see Supplementary Material S3. For more information, visit: http://www.prisma-statement.org/.

After importing the search results into EndNote 20.6 (Bld 17174), all duplicates were removed, and a title–abstract screening was carried out by two independent reviewers (KS, AS). In case of disagreement, consensus was reached by discussion or, if consensus could not be reached, by consulting a third reviewer (MR). Afterwards, all full texts were retrieved and screened again independently by both reviewers. When the title and abstract did not have sufficient information for screening purposes, a full-text copy was retrieved as well. Additionally, the bibliography of all retrieved articles was manually screened for relevant studies. Such studies were included if they provided a comprehensive description of the study. The study flow during this process is presented in Figure 1.

### 2.2. Criteria for Including and Excluding Studies in This Network Meta-Analysis

As cisplatin-ineligibility is a rather common difficulty when treating patients with HNSCC, many trials evaluating alternatives exist. To ensure maximum methodological homogeneity, statistical rigor, and a lower risk of bias, only randomized controlled trials were included in this NMA.

Trials including nasopharyngeal carcinoma (NPC) were excluded, because NPC differs distinctly from HNSCC regarding pathogenesis, treatment and outcome [2,10,11]. A publication cutoff excluding trials from before 2005 was chosen a priori to focus on contemporary treatment options and to reduce heterogeneity related to outdated RT techniques, imaging, staging, and supportive care.

The inclusion criteria were as follows:

- Patients with HNSCC undergoing definitive CRT/radioimmunotherapy (RIT)/radiochemoimmunotherapy (RCIT).
- At least one treatment arm of concomitant CRT/RIT/RCIT with active agents other than cisplatin.
- Randomization of patients between treatment arms.

Detailed inclusion and exclusion criteria are provided in Table 1.

### 2.3. Data Extraction

Data extraction was performed by two independent reviewers (KS, MR) using a standardized extraction template in Microsoft Word (Supplementary Material S4 [12,13,14,15,16,17,18,19,20,21,22,23,24,25,26,27,28,29,30,31,32,33,34,35,36,37,38,39,40,41]).

The template included:

(a) General study information: author, journal, year of publication, number of patients in general and for each treatment arm, observation period.
(b) Patient characteristics: age, sex, cancer sites, cancer stage.
(c) Intervention: type of irradiation procedure, total dose and dose per fraction, type and dose of systemic treatment.
(d) Main reported outcomes: OS, PFS, LC, dermatitis, mucositis, weight loss, acneiform rash, dysphagia, and renal impairment.

### 2.4. Assessment of Risk of Bias and Methodological Quality

All characteristics were assessed by two independent reviewers (KS, AS). Discrepancies were resolved by discussion. If consensus could not be reached, a third reviewer was consulted (MR).

The risk of bias of the included RCTs was analyzed with the Scottish Intercollegiate Guidelines Network (SIGN) Methodology Checklist 2: Controlled Trials [42]. Further, these studies were rated according to the Oxford criteria [43].

Additional criteria concerning methodology were the size of the population, application of power analysis, adequacy of statistical tests (e.g., control of premises or multiple testing), and selective outcome reporting (report of all assessed outcomes with specification of statistical data as the *p*-value), as well as possible conflicts of interest.

To assess publication bias, a funnel plot for every network with ≥10 studies was created, as recommended by Sterne et al. [44].

### 2.5. Data Synthesis

Overall survival (OS), progression-free survival (PFS), and locoregional control (LC) were the primary outcomes. These outcomes were analyzed using hazard ratios (HR). When an HR was not reported, it was estimated from its Kaplan–Meier curve using digitizeIt (version 2.5.9) and the Excel spreadsheet (version 3.0) developed by Matthew Sydes and Jayne Tierney [45,46,47]. Different treatment classes were included in the same network if they addressed the same clinical decision context: RT-based treatment of locally advanced HNSCC when cisplatin is unsuitable or serves as the comparator. Clinically distinct strategies, including altered-fractionation RT and regimens containing induction or adjuvant systemic therapy, were retained as separate nodes to avoid inappropriate pooling. Transitivity was assessed qualitatively by comparing key potential effect modifiers across trials, including age, sex, tumor site, tumor stage, performance status where available, HPV status where reported, RT technique, RT dose/fractionation, and systemic treatment regimen. Because reporting of cisplatin-ineligibility, baseline comorbidities, HPV status, performance status, and RT quality was incomplete and heterogeneous, transitivity could not be formally confirmed, and residual intransitivity was considered an important limitation.

Dermatitis, mucositis, weight loss, acneiform rash, dysphagia, and renal impairment were analyzed as secondary outcomes using odds ratios (ORs) for each. ORs were calculated from the reported percentages. As not all studies reported adverse events according to CTCAE grading, and because concomitant CRT carries a high risk of mild adverse events (grade 1–2), while toxicity-related deaths (grade 5) were inconsistently reported across studies, only grade 3–4 adverse events were extracted whenever possible. If toxicity data were not reported in sufficient detail to identify the relevant endpoint, grade, numerator, and denominator, the study was not included in the corresponding endpoint-specific adverse-event analysis. When only percentages were reported, odds ratios were calculated from these percentages and the available arm-level denominators, acknowledging the possibility of rounding errors. Missing toxicity data were not imputed.

The statistical analysis was performed using R Studio version 4.4.1 (14 June 2024) “Cranberry Hibiscus” [48].

We performed a frequentist network meta-analysis to compare the treatment options directly and indirectly. A frequentist random effects framework was chosen because it provides a transparent and reproducible approach without requiring prior assumptions, which were difficult to justify given the heterogeneity of interventions and the limited amount of direct evidence for several comparisons [49]. For the primary outcomes, the log hazard ratios and their standard errors were used. For the secondary outcomes, the log odds ratios and their standard errors were used. Additionally, to account for the slightly different populations of the studies, a random effects model was used. The tolerance of consistency had to be set to 0.5 because three three-arm studies were included.

For every outcome, a netgraph, forest plot, and treatment ranking were created. Treatment rankings were generated as exploratory descriptive summaries of the network results. If subnetworks occurred, those were analyzed individually. For the endpoint of dysphagia, the second subnetwork consisted of only one study (Rodríguez et al. [12]) and could not be compared further. The second subnetwork of weight loss consisted also of only one study (Fallai et al. [13]) and could not be compared further. For better readability, the treatment regimens of induction therapy with Docetaxel + Cisplatin + 5-FU, and concurrent Cetuximab were abbreviated as “Induction TPF + Cetuximab” and concurrent Cetuximab with adjuvant Cetuximab as “Cetuximab + adj. CTX” in plots and figures.

## 3. Results

The systematic search revealed 15,553 results. An additional 15 studies were added by hand search. At first, duplicates were removed, leaving 13,197 studies. After screening the title and abstract, 99 studies remained, which were retrieved to complete full-text screening. Finally, 52 publications of 28 studies were included in this NMA, but only 26 could be analyzed, because two studies (Nagpal et al. [14] and Argiris et al. [15]) missed a connection to the network.

Detailed characterization of the included studies may be seen in Table 2.

### 3.1. Characteristics of Included Studies

Concerning all relevant studies, 6870 patients were included, and 6687 of them were analyzed, due to 183 dropouts. The age of patients ranged from 20 to 90 years. A total of 1.147 (17.2%) participants were female, and 5.540 (82.8%) were male.

### 3.2. Excluded Studies

A list of the studies excluded after full-text screening and the reasons for exclusion are presented in the Supplementary Materials (Supplementary Material S5 [50,51,52,53,54,55,56,57,58,59,60,61,62,63,64,65,66,67,68,69,70,71,72,73,74,75,76,77,78,79,80,81,82,83,84,85,86,87,88,89,90,91,92,93,94,95,96,97,98,99,100]).

### 3.3. Risk of Bias of Included Studies

The methodical quality was rated according to the SIGN Methodology Checklist 2: Controlled Trials [42]. These studies were further rated based on the Oxford criteria [43]. These results and additional comments on methodology are provided in Supplementary Material S6. Overall, all studies were of sufficient quality and therefore included in further analysis.

### 3.4. Efficacy of Cisplatin-Free Chemoradiotherapy 

#### 3.4.1. Overall Survival

**Figure 2:**
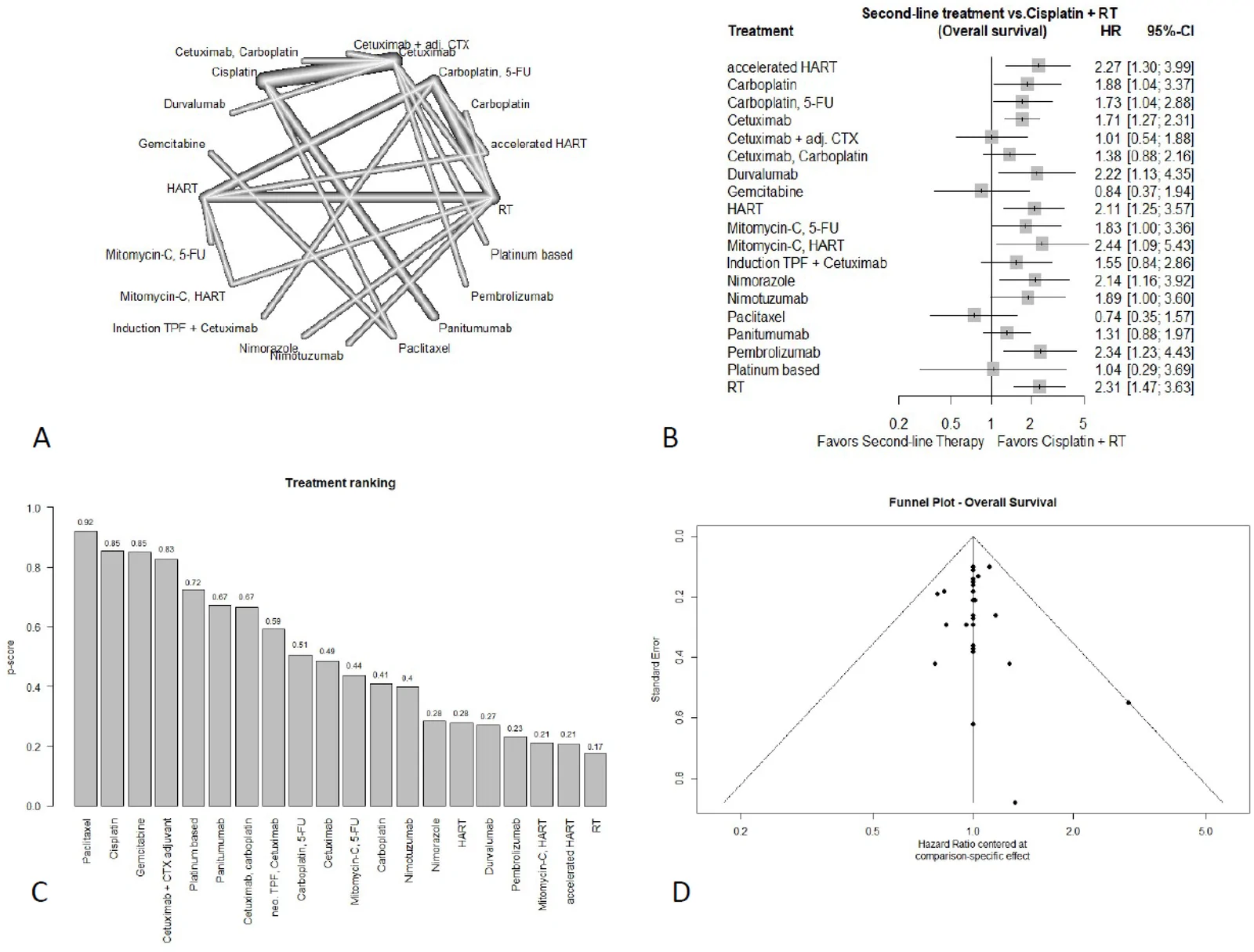
Results of OS (**A**) netgraph, (**B**) forest plot, (**C**) treatment ranking, (**D**) funnel plot.

The analysis of OS included 25 studies with 6535 patients. The corresponding netgraph is shown in Figure 2A. Compared to the standard regimen of cisplatin plus RT, no alternative treatment demonstrated a significant OS advantage. For details, see Figure 2B. A numerical improvement of OS was seen with paclitaxel (HR = 0.74; 95%-CI: 0.35–1.57) and gemcitabine (HR = 0.84; 95%-CI: 0.37–1.94). Concomitant cetuximab plus adjuvant cetuximab (HR = 1.01; 95%-CI: 0.54–1.88) and platinum-based therapy (HR = 1.04; 95%-CI: 0.29–3.69) showed comparable results to the standard regimen, while panitumumab (HR = 1.31; 95%-CI: 0.88–1.97), cetuximab, carboplatin (HR = 1.38; 95%-CI: 0.88–2.16) and induction TPF + cetuximab (HR = 1.55; 95%-CI: 0.84–2.86) showed numerically worse OS.

Carboplatin + 5-FU (HR = 1.73; 95%-CI: 1.04–2.88), cetuximab (HR = 1.71; 95%-CI: 1.27–2.31), mitomycin-C + 5-FU (HR = 1.83; 95%-CI: 1.00–3.36), carboplatin (HR = 1.88; 95%-CI: 1.04–3.37), nimotuzumab (HR = 1.89; 95%-CI: 1.00–3.60), nimorazole (HR = 2.14; 95%-CI: 1.16–3.92), HART (HR = 2.11; 95%-CI: 1.25–3.57), durvalumab (HR = 2.22; 95%-CI: 1.13–4.35), pembrolizumab (HR = 2.34; 95%-CI: 1.23–4.43), mitomycin C + HART (HR = 2.44; 95%-CI: 1.09–5.43), and accelerated HART (HR = 2.27; 95%-CI: 1.30–3.99) each showed significantly worse OS with conventional RT without concomitant systemic treatment (cRT) (HR = 2.31; 95%-CI: 1.47–3.63) being the least favorable option. The full treatment ranking can be seen in Figure 2C.

#### 3.4.2. Progression-Free Survival

**Figure 3:**
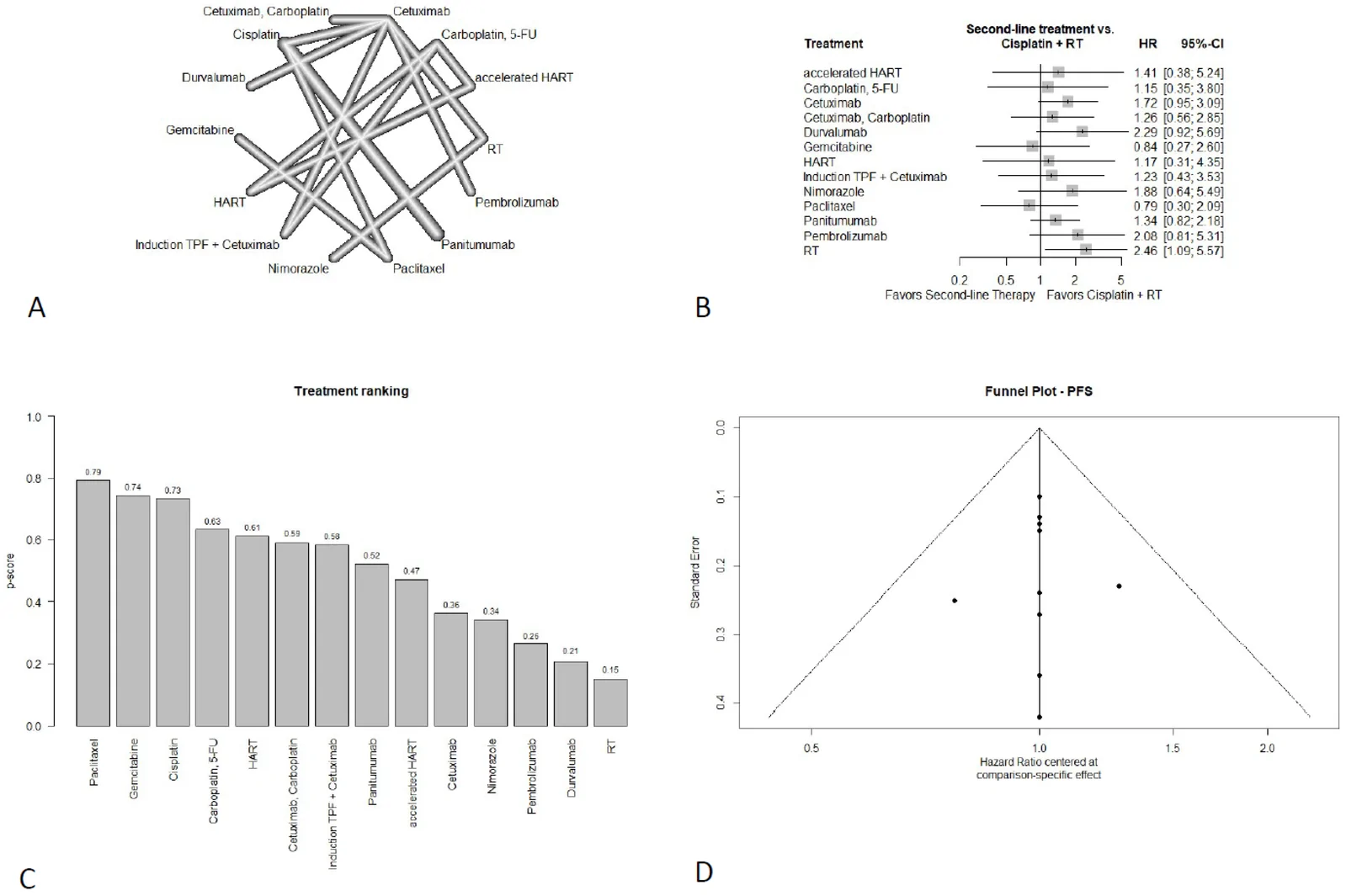
Results of PFS (**A**) netgraph, (**B**) forest plot, (**C**) treatment ranking, (**D**) funnel plot.

Thirteen studies with 4233 patients reported on PFS. The corresponding netgraph is shown in Figure 3A.

In terms of PFS, all alternative regimens failed to show a significantly superior result compared to the standard regimen of cisplatin plus RT (Figure 3B). Paclitaxel (HR = 0.79; 95%-CI: 0.30–2.09) and gemcitabine (HR = 0.84; 95%-CI: 0.27–2.60) showed a non-significant PFS improvement. Although the wide confidence interval (CI) of gemcitabine indicates substantial uncertainty regarding the true effect. Carboplatin + 5-FU (HR = 1.15; 95%-CI: 0.35–3.80), HART (HR = 1.17; 95%-CI: 0.31–4.35), cetuximab + carboplatin (HR = 1.26; 95%-CI: 0.56–2.85), induction therapy (docetaxel + cisplatin + 5-FU) plus cetuximab (HR = 1.23; 95%-CI: 0.43–3.53), panitumumab (HR = 1.34; 95%-CI: 0.82–2.18), accelerated HART (HR = 1.41; 95%-CI: 0.38–5.24), cetuximab (HR = 1.72; 95%-CI: 0.95–3.09), nimorazole (HR = 1.88; 95%-CI: 0.64–5.48), pembrolizumab (HR = 2.08; 95%-CI: 0.81–5.31), and durvalumab (HR = 2.29; 95%-CI: 0.92–5.69) each were associated with numerically worse PFS without statistical significance. cRT provided significantly worse PFS (HR = 2.46; 95%-CI: 1.09–5.57), thus being the least favorable option. The full treatment ranking can be seen in Figure 3C.

#### 3.4.3. Locoregional Control

For the endpoint of LC, two separate subnetworks were analyzed due to a missing connection. The first subnetwork consisted of four studies with 721 patients, and the second included four studies with 610 patients.

##### Subnetwork 1

**Figure 4:**
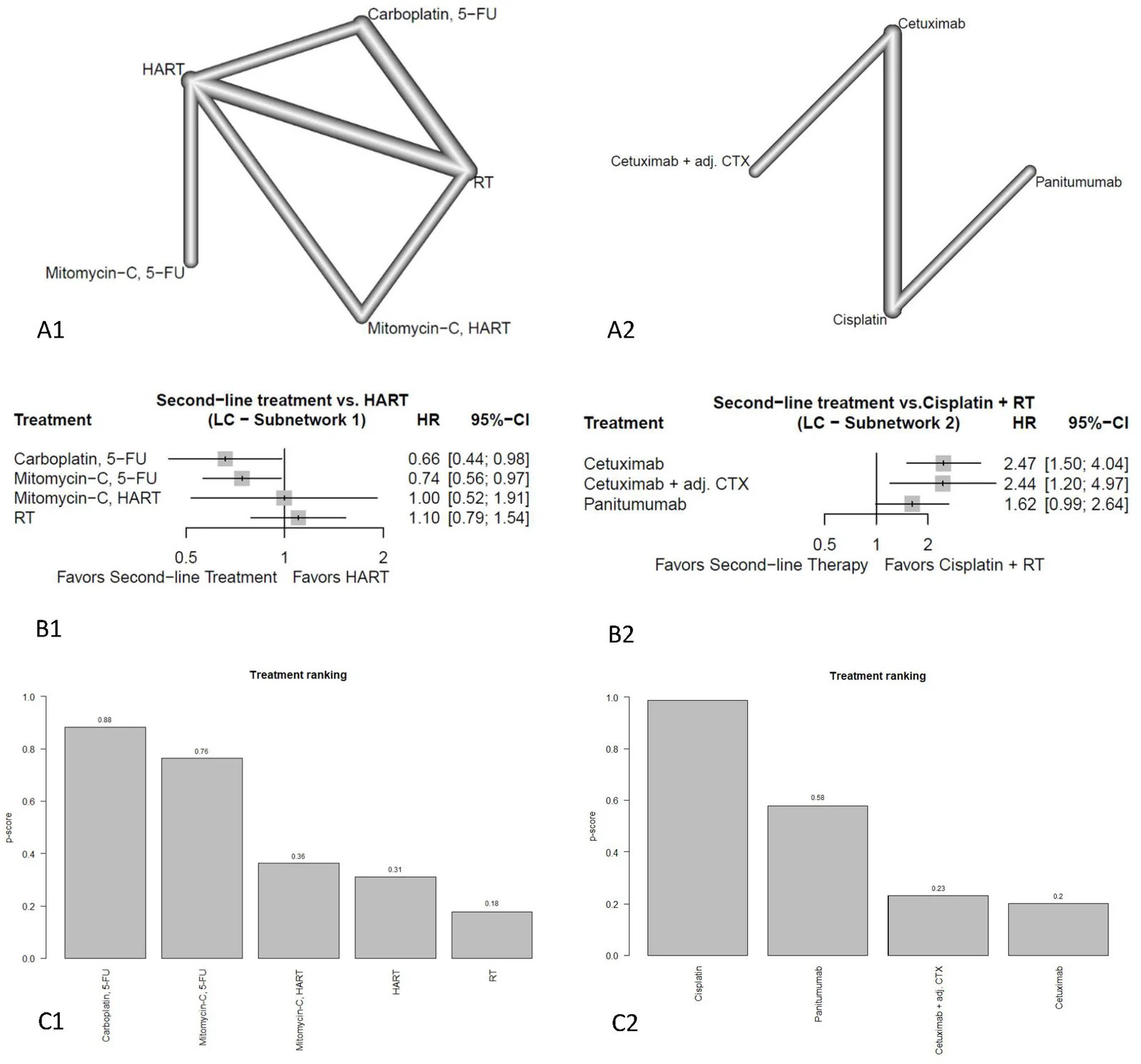
Results of LC (**A1**) netgraph subnetwork 1, (**A2**) netgraph subnetwork 2, (**B1**) forest plot subnetwork 1, (**B2**) forest plot subnetwork 2, (**C1**) treatment ranking subnetwork 1, (**C2**) treatment ranking subnetwork 2.

The netgraph of subnetwork 1 is shown in Figure 4(A1).

Compared to HART treatment with carboplatin + 5-FU (HR = 0.66; 95%-CI: 0.44–0.98) and mitomycin C + 5-FU (HR = 0.74; 95%-CI: 0.56–0.97) showed a significant improvement of LC (Figure 4(B1)). Mitomycin C plus HART showed comparable results (HR = 1.00; 95%-CI: 0.52–1.91) to HART alone, although the wide CI indicates substantial uncertainty regarding the true effect. cRT (HR = 1.10; 95%-CI: 0.79–1.54) showed numerically worse LC than HART. The full treatment ranking can be seen in Figure 4(C1).

##### Subnetwork 2

The netgraph of subnetwork 2 is shown in Figure 4(A2).

In terms of LC, all regimens failed to show improvement compared to the standard regimen of cisplatin plus RT (Figure 4(B2)). While panitumumab was numerically worse (HR = 1.62; 95%-CI: 0.99–2.64), both alternative regimens, concomitant cetuximab plus adjuvant cetuximab (HR = 2.44; 95%-CI: 1.20–4.97) and concomitant cetuximab only (HR = 2.47; 95%-CI: 1.50–4.04) showed significantly worse results. The full treatment ranking can be seen in Figure 4(C2).

### 3.5. Adverse Events

#### 3.5.1. Dysphagia

Six studies reported on dysphagia, but because of a missing connection to the network, the study of Rodríguez et al. [12] could not be compared further, so only five studies, including 805 patients, remained for analysis. The netgraph is shown in Supplementary Material S7A. Durvalumab (OR = 0.37; 95%-CI: 0.15–0.89) and cetuximab showed significantly less dysphagia (OR = 0.55; 95%-CI: 0.33–0.93) compared to the standard regimen of cisplatin plus RT, while both panitumumab (OR = 1.32; 95%-CI: 0.77–2.25) and paclitaxel (OR = 1.60; 95%-CI: 0.38–6.81) showed numerically worse results (Supplementary Material S7B). The full treatment ranking can be seen in Supplementary Material S7C.

#### 3.5.2. Acneiform Rash

Seven studies, including 2358 patients, reported on acneiform rash. The netgraph is shown in Supplementary Material S8A. Compared to the standard regimen of Cisplatin plus RT, all studies failed to show significantly superior results (Supplementary Material S8B). Pembrolizumab (OR = 2.18; 95%-CI: 0.09–52.90) and cRT (OR = 2.41; 95%-CI: 0.32–18.01) showed numerically worse results; however, their wide CI indicated substantial uncertainty regarding their true effect.

Cetuximab (OR = 52.29; 95%-CI: 12.81–213.50) and induction therapy (docetaxel + cisplatin + 5-FU) plus cetuximab (OR = 59.26; 95%-CI: 11.86–296.22) showed significantly worse results. Panitumumab (OR = 864.77; 95%-CI: 191.84–3898.23) was the least favorable treatment regimen and was associated with a highly significant increase in the risk of acneiform rash. The full treatment ranking can be seen in Supplementary Material S8C.

#### 3.5.3. Renal Impairment

Seven studies, including 1978 patients, reported on renal impairment occurring during treatment. The netgraph for the endpoint of renal impairment is shown in Supplementary Material S9A. Compared to the standard regimen of cisplatin plus RT, only treatment with cetuximab showed significantly less renal impairment (OR = 0.07; 95%-CI: 0.02–0.25), while panitumumab (OR = 0.32; 95%-CI: 0.06–1.60) and durvalumab (OR = 0.14; 95%-CI: 0.00–4.11) only showed a numerical reduction, with durvalumab also showing a notably wide confidence interval. Platinum-based combination therapy (OR = 0.91; 95%-CI: 0.04–23.20) demonstrated no statistically significant difference, with a notably wide confidence interval. Cetuximab + carboplatin (OR = 1.71; 95%-CI: 0.08–38.65) showed numerically worse results, see Supplementary Material S9B. The full treatment ranking can be seen in Supplementary Material S9C.

#### 3.5.4. Dermatitis

For dermatitis, two separate subnetworks were analyzed due to a missing connection. The first subnetwork consisted of 12 studies with 1948 patients, and the second included 5 studies with 1015 patients.

##### Subnetwork 1

The netgraph of subnetwork 1 is shown in Supplementary Material S9(A1). Compared to the standard regimen of Cisplatin plus RT, all alternative regimens failed to show a significant reduction in dermatitis incidence. Both panitumumab (OR = 0.43; 95%-CI: 0.11–1.67) and pembrolizumab (OR = 0.81; 95%-CI: 0.17–3.73) showed a numerically reduced incidence of dermatitis, while durvalumab (OR = 1.63; 95%-CI: 0.29–9.12), paclitaxel (OR = 2.12; 95%-CI: 0.26–17.51), concomitant cetuximab with adjuvant cetuximab (OR = 2.21; 95%-CI: 0.06–86.43), platinum-based therapy (OR = 5.65; 95%-CI: 0.09–370.52) and gemcitabine (OR = 3.92; 95%-CI: 0.34–44.99) showed a numerically higher incidence of dermatitis. Cetuximab (OR = 4.63; 95%-CI: 2.14–10.02) and cetuximab + carboplatin (OR = 6.30; 95%-CI: 1.15–34.54) showed a significantly higher incidence of treatment-related dermatitis Supplementary Material S10(B1). The full treatment ranking can be seen in Supplementary Material S10(C1).

##### Subnetwork 2

The netgraph of subnetwork 2 is shown in Supplementary Material S10(A2). Compared to HART, all studies failed to show a significant reduction in dermatitis incidence Supplementary Material S10(B2). Nimotuzumab (OR = 2.32; 95%-CI: 0.25–21.44), cRT (OR = 2.48; 95%-CI: 0.44–14.07), and carboplatin, 5-FU (OR = 4.44; 95%-CI: 0.84–23.47) showed numerically worse results, while induction therapy with TPF plus concurrent cetuximab (OR = 12.48; 95%-CI: 1.96–79.68) showed a significantly higher incidence of dermatitis. The full treatment ranking can be seen in Supplementary Material S10(C2).

#### 3.5.5. Weight Loss

Six studies reported on weight loss, but because of a missing connection to the network, the study of Fallai et al. [13] could not be compared further, so that five studies, including 1274 patients, remained for analysis. The netgraph is shown in Supplementary Material S11A. While panitumumab (OR = 0.15; 95%-CI: 0.03–0.66) and cetuximab (OR = 0.66; 95%-CI: 0.47–0.92) showed significantly less incidence of weight loss compared to the standard regimen of cisplatin plus radiotherapy, durvalumab (OR = 0.68; 95%-CI: 0.26–1.78) and platinum-based combination therapy showed an only numerically lower incidence of weight loss (OR = 0.8; 95%-CI: 0.01–43.17) Supplementary Material S11B. Platinum-based combination therapy also showed a notably wide confidence interval. The full treatment ranking can be seen in Supplementary Material S11C.

#### 3.5.6. Mucositis

Eighteen studies, including 3.836 patients, reported on the incidence of mucositis. The corresponding netgraph can be seen in Supplementary Material S12A. All alternative regimens failed to show a significant decrease in mucositis incidence compared to cisplatin plus RT Supplementary Material S12B. Pembrolizumab (OR = 0.57; 95%-CI: 0.07–4.55) showed a numerical decrease, while durvalumab (OR = 0.96; 95%-CI: 0.11–8.12), as well as panitumumab (OR = 1.00; 95%-CI: 0.36–2.81), provided comparable results to Cisplatin plus RT. RT (OR = 1.61; 95%-CI: 0.11–23.67), cetuximab (OR = 1.72; 95%-CI: 0.41–7.12), nimotuzumab (OR = 1.92; 95%-CI: 0.08–45.17), Induction TPF plus cetuximab (OR = 2.01; 95%-CI: 0.26–15.73), carboplatin + 5-FU (OR = 2.19; 95%-CI: 0.18–26.33), platinum-based therapy (OR = 2.10; 95%-CI: 0.03–174.93), carboplatin (OR = 2.30; 95%-CI: 0.10–54.80), paclitaxel (OR = 2.73; 95%-CI: 0.42–17.92), HART (OR = 3.22; 95%-CI: 0.22–46.12), gemcitabine (OR = 4.74; 95%-CI: 0.44–51.41), cetuximab plus adjuvant CTX (OR = 5.27; 95%-CI: 0.26–107.77), cetuximab + carboplatin (OR = 5.31; 95%-CI: 0.42–66.74), accelerated HART (OR = 5.21; 95%-CI: 0.31–86.43) and mitomycin C + HART (OR = 12.12; 95%-CI: 0.45–322.98) all showed a numerical increase in mucositis incidence. The full treatment ranking can be seen in Supplementary Material S12C.

Only the PFS and OS network, the first dermatitis subnetwork, and the mucositis network included at least 10 studies. Therefore, funnel plots were generated only for these networks. The funnel plot was evenly distributed for PFS and the first subnetwork of dermatitis. The funnel plot for mucositis also showed a symmetrical distribution, but also two scattered points outside the 95%-CI funnel. Those scattered points may be due to a small-study effect. The funnel diagram for OS showed a biased distribution with a scattered point in the middle of the funnel, indicating a possible publication bias, which may have also impacted the results. All funnel plots can be seen in Figure 2D and Figure 3D and Supplementary Material S13.

## 4. Discussion

Surgical resection followed by adjuvant CRT is the standard of care treatment for locally advanced HNSCC with a curative-intended approach. If functional unresectability or contraindications for surgery exist, definitive CRT is the treatment of choice. High-dose cisplatin and concurrent RT is the standard of care treatment in such cases [3]. However, toxicity and comorbidities restrict the applicability of cisplatin [4], the optimal treatment strategy for patients with locally advanced, non-metastatic HNSCC who are ineligible for cisplatin remains uncertain.

For the endpoint of OS, no regimen was superior to standard of care treatment with cisplatin. In case of cisplatin-ineligibility, both paclitaxel and gemcitabine showed numerically favorable but statistically non-significant OS estimates compared to cisplatin and therefore may represent potentially suitable non-cisplatin options in selected patients. The precision of these estimates is limited by wide confidence intervals, largely attributable to small sample sizes and a high proportion of indirect comparisons. Additionally, potential publication bias has to be considered, as evident in the funnel plot, so that these findings should be interpreted with caution.

None of the included agents showed a significant improvement in PFS compared to cisplatin, but paclitaxel and gemcitabine ranked ahead of cisplatin for PFS. However, for both substances, the wide CI leaves space for substantial uncertainty regarding their true effect, which should be considered when applying these results. It must also be mentioned that the network had a high proportion of indirect comparisons and studies with small sample sizes, leading to wide confidence intervals and limited precision and certainty of these findings. Therefore, these findings should not be interpreted as evidence of superiority. Rather, they may inform consideration of these regimens as potential non-cisplatin options in selected patients.

For the endpoint of LC carboplatin + 5-FU and mitomycin + 5-FU both showed significant improvement compared to HART in the first subnetwork, while no alternative treatment could improve LC in the second subnetwork compared to cisplatin. However, both subnetworks only included data from four studies, so the evidence base is limited.

In this NMA, none of the alternative concomitant systemic regimens significantly improved OS, PFS, or LC compared with cisplatin for patients with locally advanced HNSCC. Consequently, alternative chemoradiotherapy (CRT) regimens should be reserved for patients with clear contraindications to cisplatin. In this context, paclitaxel and gemcitabine may represent potentially suitable non-cisplatin options in selected patients, as both agents showed numerically favorable or comparable outcomes regarding OS and PFS without statistically significant differences relative to cisplatin. However, these findings remain limited by wide confidence intervals, small sample sizes, and a high proportion of indirect comparisons. Unfortunately, no comparative studies with paclitaxel versus gemcitabine CRT regimen exist, so a comparison between the two drugs is only indirectly possible.

Our findings are partially in line with current NCCN guidelines, where CRT with cisplatin is recommended as the standard of care, preferably delivered as high-dose treatment, alongside carboplatin + 5-FU [101]. However, neither paclitaxel nor gemcitabine is currently recommended as a systemic agent during CRT. A recently conducted NMA by Petrelli et al. [102] recommended cisplatin CRT as the standard of care, with concomitant carboplatin or docetaxel as alternatives for cisplatin-unfit patients. However, it should be mentioned that the differences between their results and ours might be due to the different statistical approaches used, as Petrelli et al. used a Bayesian framework with a fixed effects model, while our NMA was performed using a frequentist framework with a random effects model, thus leading to slightly different results due to the prior assumptions of the methods used [49,103]. Furthermore, Petrelli et al. employed different inclusion criteria, as adjuvant and neoadjuvant regimens, studies with only HPV-positive patients, experimental agents, and studies with anti-EGFR treatment were excluded. Additionally, our NMA ruled out studies that were published before 2005, which were included by Petrelli et al.

Given the prevalence of cisplatin ineligibility due to baseline comorbidities, the selection of alternative regimens requires a rigorous evaluation of their comparative adverse event (AE) profiles to ensure treatment tolerability. Compared to cisplatin, both cetuximab and durvalumab emerged as less-toxic alternatives with a significantly lower incidence of dysphagia. Cetuximab further demonstrated a significant reduction in treatment-related renal impairment and weight loss; panitumumab showed similar benefits regarding weight loss, though its impact on renal function was only numerically superior. In contrast, no regimen significantly reduced the incidence of acneiform rash or mucositis. The high incidence of acneiform rash is consistent with the pharmacological profiles of immune checkpoint inhibitors [104,105]. For dermatitis and mucositis, several agents (pembrolizumab, durvalumab, and panitumumab) showed numerical improvements, but results were statistically non-significant. The interpretation of these findings is constrained by small sample sizes (typically five studies per endpoint) and a high proportion of indirect comparisons, resulting in notably wide confidence intervals (CIs). Specifically, the mucositis analysis exhibited potential small-study effects or statistical outliers, as indicated by funnel plot asymmetry. Consequently, these results should be interpreted with caution. Overall, cetuximab and panitumumab demonstrated superior toxicity profiles compared to cisplatin, with cetuximab identified as the preferred alternative due to significant reductions in overall toxicity. These findings align with ESTRO guidelines [3], which maintain cisplatin-based CRT as the gold standard for locally advanced HNSCC while recommending cetuximab for cisplatin-ineligible patients. Although panitumumab showed comparable benefits in this network meta-analysis, it is not yet explicitly endorsed by ESTRO. However, panitumumab was not explicitly recommended by ESTRO guidelines, although both studies by Giralt et al. [28] and Siu et al. [38] were published at the time. Our results corroborate the results of previous meta-analyses [106] confirming cetuximab as a less-toxic alternative for the cisplatin-unfit population.

Some limitations of this NMA must be mentioned. For once, only studies published in English or German were included in this review. In consequence, most of the included studies were performed in North America and Europe, leading to an overrepresentation of these populations in our data. Therefore, our findings might not be generalizable to all populations [1,2]. In the absence of a standardized definition of cisplatin ineligibility, eligibility criteria vary substantially across studies, and well-conducted studies directly addressing this research question are scarce. Consequently, our study selection criteria had to navigate the tension between heterogeneity and a limited data basis, which presents an inherent limitation of data accuracy. Because several trials enrolled broader LA-HNSCC populations rather than exclusively cisplatin-ineligible patients, our findings should be interpreted as evidence on cisplatin-free RT-based regimens relevant to the cisplatin-ineligible setting, rather than as evidence generated solely in cisplatin-unfit populations. Resulting differences in frailty, comorbidities, and performance status between study populations may have introduced indirectness and residual intransitivity, potentially affecting both efficacy and toxicity comparisons. Additionally, since HPV status and HPV-stratified outcomes were inconsistently reported, imbalances in HPV prevalence across trials could not be adjusted for and may have affected indirect comparisons, particularly for survival endpoints. Also, only studies published in 2005 or later were included. This was done to minimize the heterogeneity of irradiation techniques and account for technical developments, such as the use of IMRT or 3D conformal RT, as well as improvements in supportive care, HPV testing and stratification, imaging, target delineation, and staging systems, which all have the potential to significantly affect oncologic outcomes and adverse events [107]. However, as demonstrated by a study from Peters et al. [108], publication after 2005 does not guarantee homogeneous RT quality. Differences in RT quality assurance and treatment planning could not be adjusted for, although such factors may affect oncologic outcomes in head and neck cancer. RT technique, dose, and fractionation were extracted, and altered fractionation schedules were modeled as separate treatment nodes where possible. However, because radiation delivery parameters were heterogeneously reported and unevenly distributed across comparisons, we could not perform a robust subgroup analysis or meta-regression, leaving residual RT-related heterogeneity as a limitation. The same limitation applies to possible subgroup analyses by HPV status and to subgroup-like treatment strategies, such as cetuximab plus adjuvant cetuximab and induction TPF plus cetuximab. As the nodes of these non-concurrent regimens were supported by few studies and were retained separately only because they represented clinically distinct interventions, their results should be interpreted with special caution. Late toxicity, quality of life, feeding tube dependence, and other functional outcomes could not be robustly analyzed because too few studies reported these endpoints using consistent definitions and time points. Additionally, excluding studies from before 2005 may have excluded earlier landmark CRT trials, potentially reducing network completeness and introducing selection bias.

Regimens containing induction or adjuvant systemic therapy were analyzed as separate nodes and not pooled with purely concurrent CRT regimens. Because these strategies represent distinct treatment paradigms and were supported by few studies, their estimates and rankings should be interpreted as exploratory. Furthermore, some HR were estimated from their Kaplan–Meier curves and OR from reported percentages, both methods allowing for rounding errors. Due to the selection of included studies, the network structure resulted in a high proportion of indirect comparisons and subnetworks with few patients, which are prone to confounding and also increase statistical inaccuracy through small-study effects [109]. Also, a high proportion of results showed a wide 95%-CI, so a substantial uncertainty regarding their true effect remains. Furthermore, several comparisons resulted in statistically non-significant results, which might be due to the selection of cisplatin as the reference, but more importantly, due to the high proportion of indirect comparisons. Due to these limitations, treatment rankings should be interpreted with caution. Therefore, rankings should not be considered a substitute for relative effect estimates or clinical judgment. Also, some studies had rather small sample sizes (e.g., Al-Saleh et al. [16], Essa et al. [22], Ezzat et al. [23]), which further adds to statistical dispersion and thus distortion of results. Also, the funnel plots of mucositis and OS hinted at a possible publication bias in the included studies, as well as possible bias through small-study effects. Although a frequentist random effects framework was used to account for between-study heterogeneity, the sparse network structure and high proportion of indirect comparisons limited the precision of several estimates. A Bayesian approach might have been an alternative, but results in such sparse networks may be sensitive to the choice of prior distributions.

## 5. Conclusions

Carboplatin + 5-FU and Mitomycin C + 5-FU improve LC compared to HART.

Compared to cisplatin, durvalumab and cetuximab are both associated with less dysphagia, while cetuximab is associated with less renal impairment compared to cisplatin. Panitumumab and cetuximab are associated with a decreased incidence of weight loss.

Overall, the findings of this analysis can show a trend in possible treatment options, but the evidence base is too limited for a definitive statement, and further research is necessary.

## 6. Highlights

- Network meta-analysis of non-cisplatin RT-based regimens relevant to cisplatin-ineligible LA HNSCC with RT.
- 28 RCTs (≈7000 patients) compared non-cisplatin concurrent systemic options.
- No regimen improved OS, PFS or LC versus cisplatin-based chemoradiotherapy.
- Carboplatin + 5-FU and mitomycin C + 5-FU improved LC versus HART.
- Durvalumab/cetuximab were associated with less dysphagia, and cetuximab with less renal toxicity and weight loss.

## Figures and Tables

**Table 1:** Inclusion and exclusion criteria based on PICO.

PICO	Inclusion Criteria	Exclusion Criteria
Patient	Patients with HNSCC undergoing definitive radiochemotherapy or radioimmunotherapy or radiochemoimmunotherapy	Patients with precancerous conditions or Carcinoma in situ Preclinical studies Nasopharyngeal carcinoma
Intervention	Concomitant radiochemotherapy or radioimmunotherapy or radiochemoimmunotherapy with active agents other than cisplatin	radiochemotherapy with cisplatin as an active agent in all treatment arms chemotherapy or immunotherapy or chemoimmunotherapy as only adjuvant/neoadjuvant/induction therapy palliative approach
Comparison	All possible control groups (active control, placebo, standard/guideline/usual care) Randomized study	Single-arm study No randomization
Outcome	Overall survival Progression/disease-free survival Toxicity and adverse events	
Others	Language: German and English Full publication Published 2005—today	Grey literature (conference articles, abstracts, letters, ongoing studies, unpublished literature…) Full text not available in German or English Published before 2005

**Table 2:** Characteristics of included studies.

The Basic Information of All Included Studies in the Network Meta-Analysis								
Author	Year	Pat. (n)	Age (y)	Fem (%)	CT Regimen	Dose of CT	RT Technique	RT Regimen (in Gy)
Al-Saleh et al. [16]	2019	40	27–72	12.5	pbCT vs. CTX	Cisplatin: 100 mg/m^2^; 3-weekly or 40 mg/m^2^ weekly Cetuximab: loading dose 400 mg/m^2^; 250 mg/m^2^ during radiation	IMRT	69.6 Gy/1.2 Gy twice daily
Argiris et al. [15]	2016	80	35–76	19.2	CTX, PMX vs. CTX, PMX, Beva + maintenance	Cetuximab: loading dose 400 mg/m^2^; 250 mg/m^2^ during radiation Pemetrexed: 500 mg/m^2^ on days 1, 22, 43 Bevacizumab: 15 mg/kg on days 1, 22, 43 + 6 months maintenance	RT	70–74 Gy/2 Gy
Bonner et al. [17]	2006	424	34–83	19.8	CTX vs. RT	Cetuximab: loading dose 400 mg/m^2^; 250 mg/m^2^ during radiation	RT	Once daily: 70 Gy/2 Gy Twice daily: 72.0–76.8 Gy/1.2 Gy
Bourhis et al. [18]	2012	840	34–75	13	CBDP, 5-FU vs. HART vs. Acc HART	RT: Carboplatin: 70 mg/m^2^ on days 1–4, 22–25, 43–46 5-FU: 600 mg/m^2^ on days 1–4, 22–25, 43–46 HART: Carboplatin: 70 mg/m^2^ on days 1–5, 29–33 5-FU: 600 mg/m^2^ on days 1–5, 29–33	RT	RT: 70 Gy/2 Gy HART: 70 Gy/2 Gy/fraction until 40 Gy, then 1.5 Gy/fraction twice daily RT 5 days per week acc HART: 64.8 Gy/1.8 Gy twice daily
Budach et al. [19,20]	2005	384	33–71	16.1	MitC, 5-FU vs. HART	5-FU: 600 mg/m^2^ days 1 to 5 Mitomycin-C: 10 mg/m^2^ on days 5 and 36	RT	RT: 30 Gy/2 Gy followed by 1.4 Gy twice daily to 70.6 Gy HART: 16 Gy/2 Gy followed by 1.4 Gy twice daily to 77.6 Gy
Chitapanarux et al. [21]	2013	85	20–77	24.7	RT vs. CBDP, 5-FU	Carboplatin: 70 mg/m^2^ on days 1, 22, 43 5-FU: 600 mg/m^2^ on days 1, 22, 43	RT	RT: 70 Gy 2 Gy/fraction until 40 Gy, then 1.8 Gy first RT/day and 1.2 Gy second RT/day RT 5 days per week CRT: 66 Gy/2 Gy 5 fractions per week
Essa et al. [22]	2010	41	Mean: 55.1/55.7	19.5	Pacli vs. CDDP	Paclitaxel: 30 mg/m^2^ weekly Cisplatin: 30 mg/m^2^ weekly	RT	66–70 Gy/2 Gy
Ezzat et al. [23]	2005	60	Mean: 53/54/49	75	RT vs. HART vs. MitC, HART	Mitomycin C: 15 mg/m^2^ at the end of 1st week	RT	68 Gy/2 Gy RT: 5 fractions per week HART: 6 fractions per week
Fallai et al. [13,24]	2006	192	Median: 56.1	11.5	RT vs. HART vs. CBDP,5-FU	Carboplatin: 75 mg/m^2^, days 1–4 5-FU: 1000 mg/m^2^, days 1–4	RT	RT: 66–70 Gy/2 Gy HART: 64–67.2 Gy 2 fractions daily with 1.6 Gy
Gebre-Medhin et al. [25]	2021	298	33–77	20	CDDP vs. CTX	Cisplatin: 40 mg/m^2^ Cetuximab: loading dose of 400 mg/m^2^, seven weekly doses of 250 mg/m^2^	RT	68 Gy/2 Gy
Geoffrois et al. [26]	2018	370	Median: 56/56.5	15/13	Induction TPF, CTX vs. CBDP, 5-FU	TPF: docetaxel 75 mg/m^2^ day 1 + cisplatin 75 mg/m^2^ day 1 + FU 750 mg/m^2^ days 1 to 5 Cetuximab: loading dose of 400 mg/m^2^, 250 mg/m^2^ during radiation Carboplatin: 70 mg/m^2^ daily 5-FU: 600 mg/m^2^ days 1 to 4	RT	70 Gy/2 Gy
Gillison et al. [27]	2019	849	33–83	10	CDDP vs. CTX	Cisplatin: 100 mg/m^2^ days 1 and 22 Cetuximab: loading dose of 400 mg/m^2^, 250 mg/m^2^ during radiation	IMRT	70 Gy/2 Gy
Giralt et al. [28]	2015	151	NG	19	Pani vs. CDDP	Cisplatin: 100 mg/m^2^ days 1 and 22 Panitumumab: 9.0 mg/kg days 1, 22, 43	IMRT/3D conformal RT	70–72 Gy/2–2.4 Gy
Halim et al. [29]	2012	216	20–70	39/43	Gem vs. Pacli	Gemcitabine: 100 mg/m^2^ weekly Paclitaxel: 20 mg/m^2^ weekly	RT	65 Gy/2 Gy
Magrini et al. [30]	2016	70	36–80	28.6	CDDP vs. CTX	Cisplatin: 40 mg/m^2^ Cetuximab: loading dose of 400 mg/m^2^, 250 mg/m^2^ during radiation	3D conformal RT, IMRT, IMRT with simultaneous boost, helical IMRT	70 Gy/2 Gy
Mehanna et al. [31]	2019	334	52–63	20	CDDP vs. CTX	Cisplatin: 100 mg/m^2^, days 1, 22, 43 Cetuximab: loading dose of 400 mg/m^2^, 250 mg/m^2^ during radiation	RT	70 Gy/2 Gy
Mell et al. [32]	2024	190	48–90	16	Durvalumab vs. CTX	Durvalumab: 1500 mg^2^ weeks before RT, every 4 weeks starting week 2 Cetuximab 400 mg/m^2^ 1 week before RT, 250 mg/m2 weekly	RT	70 Gy/2 Gy
Mercke et al. [33]	2023	152	Median: 59.8	24.6	Induction TPF, CTX vs. CTX	Induction CT 2 cycles Docetaxel: 75 mg/m^2^ 2 cycles Cisplatin: 75 mg/m^2^ 2 cycles 5-FU: 1000 mg/m^2^ cycles 21 days apart during RT Cetuximab: loading dose of 400 mg/m^2^, 250 mg/m^2^ during radiation	RT	68 Gy/2 Gy
Mesia et al. [34]	2012	91	42–80	14.3	CTX vs. CTX, adj. CTX	Cetuximab: loading dose of 400 mg/m^2^, 250 mg/m^2^ during radiation adj. CTX: 250 mg/m^2^ weekly over 12 weeks	RT	Concurrent: 70 Gy/2 Gy adjuvant: 69.9 Gy/1.8 Gy
Nagpal et al. [14]	2021	49	42–76	10.2	CBDP, Gef vs. CBDP, Erl	Carboplatin: AUC 2 Gefitinib: 250 mg OD Erlotinib: 250 mg OD	RT	66 Gy/2 Gy
Rischin et al. [35]	2021	189	Median: 57.4	10	CDDP vs. CTX	Cisplatin: 40 mg/m^2^ weekly Cetuximab: loading dose of 400 mg/m^2^, 250 mg/m^2^ during radiation	IMRT	70 Gy/2 Gy
Rodríguez et al. [12]	2010	105	Median: 59/65	22.6	Nimo vs. Placebo	Nimotuzumab: 200 mg administered 6× during treatment Placebo: 6× during treatment	RT	60–66 Gy/2 Gy
Ruo Redda et al. [36]	2010	157	Median: 60	10	RT vs. CBDP	Carboplatin 45 mg/m^2^ on days 1–5, at weeks 1, 3, 5, 7	RT	70 Gy/2 Gy
Semrau et al. [37]	2006	263	28–73	15	RT vs. CBDP, 5-FU	Carboplatin: 70 mg/m^2^ 5-FU: 600 mg/m^2^ days 1–5 and 29–33	RT	69.9 Gy/1.8 Gy
Siu et al. [38]	2017	315	Median: 56	16	CDDP vs. Pani	Cisplatin: 100 mg/m^2^ on days 1, 22, 43 Panatimumab: 9 mg/kg every 3 weeks, starting one week before RT (days 7, 15, 36)	RT	70 Gy/2 Gy
Tao et al. [39]	2018	406	36–70	16	CTX vs. CTX, CBDP	Cetuximab: loading dose of 400 mg/m^2^, 250 mg/m^2^ during radiation Carboplatin: 70 mg/m^2^; 3 cycles on days 1–4 5-FU: 600 mg/m^2^; 3 cycles on days 1–4	IMRT	70 Gy/2 Gy
Tao et al. [40]	2023	131	47–81	14.5	Pembro vs. CTX	Pembrolizumab: 200 mg on days 1, 22, 43 during RT Cetuximab: loading dose of 400 mg/m^2^ on day 8, 250 mg/m^2^ weekly during radiation	IMRT	69.96 Gy/2.12 Gy
Thompson et al. [41]	2023	338	45–84	22.5	Placebo vs. Nimorazole	Nimorazole: 1.2 g/m^2^ Placebo: 1.2 g/m^2^	IMRT	65 Gy/2.17 Gy

Acc HART, accelerated HART; adj CTX, adjuvant cetuximab; Beva, bevacizumab; CBDP, carboplatin; CDDP, cisplatin; CTX, cetuximab; Erl, erlotinib; Fem, female; Gef, gefitinib; Gem, gemcitabine; Induction TPF, induction docetaxel, 5-fluorouracil, cisplatin; MitC, mitomycin-C; Nimo, nimotuzumab; Pacli, paclitaxel; Pani, panitumumab; Pat., Patients; pbCT, platinum-based chemotherapy; Pembro, pembrolizumab; PMX, pemetrexed; n, number; y, years; CT, chemotherapy; RT, radiotherapy; Gy, grays; IMRT, intensity-modulated radiation therapy.

## Data Availability

Data is contained within the article or Supplementary Materials.

## Supplementary Materials

The following supporting information can be downloaded at: https://www.mdpi.com/article/10.3390/cancers18101599/s1, Material S1: The PROSPERO final protocol; Material S2: Search strings used in network meta-analysis; Material S3: PRISMA checklist; Material S4: Detailed characteristics of included studies with results for treatment outcome and toxicity [12,13,14,15,16,17,18,19,20,21,22,23,24,25,26,27,28,29,30,31,32,33,34,35,36,37,38,39,40,41]; Material S5: Table of studies excluded after full-text screening [50,51,52,53,54,55,56,57,58,59,60,61,62,63,64,65,66,67,68,69,70,71,72,73,74,75,76,77,78,79,80,81,82,83,84,85,86,87,88,89,90,91,92,93,94,95,96,97,98,99,100]; Material S6: Assessment of risk of bias according to “SIGN Methodology Checklist 2: Controlled Trials” and rating according to Oxford criteria; Material S7: Results for dysphagia; Material S8: Results for acneiform rash; Material S9: Results for renal impairment; Material S10: Results for dermatitis; Material S11: Results for weight loss; Material S12: Results for mucositis; Material S13: Results of funnel plots.

## Abbreviations

The following abbreviations are used in this manuscript:

5-FU	5-fluorouracil
95%-CI	95 percent confidence interval
AE	adverse events
CI	confidence interval
CRT	chemoradiotherapy
cRT	conventional radiotherapy without concomitant systemic treatment
CT	chemotherapy
HART	hyperfractionated accelerated radiotherapy
HNSCC	squamous cell carcinoma of the head and neck
HR	hazard ratio
IT	immunotherapy
LC	locoregional control
MeSH	Medical subject heading
NMA	network meta-analysis
NPC	nasopharyngeal carcinoma
OR	odds ratio
OS	overall survival
PFS	progression-free survival
PRISMA	Preferred Reporting Items for Systematic Reviews and Meta-Analyses
RCIT	radiochemoimmunotherapy
RCT	randomized controlled trial
RIT	radioimmunotherapy
RT	radiotherapy
SIGN	Scottish Intercollegiate Guidelines Network
WoS	Web of Science Core Collection
