# Correction: Schöbel et al. Non-Cisplatin Concurrent Systemic Therapy with Radiotherapy for Locally Advanced Head and Neck Squamous Cell Carcinoma: A Network Meta-Analysis of Randomized Clinical Trials. *Cancers* 2026, *18*, 1599

**DOI:** 10.3390/cancers18213466

**Published:** 2026-10-27

**Authors:** Katharina Sophie Schöbel, Anne-Josephin Schoele, Georg Wurschi, Klaus Pietschmann, Maximilian Römer

**Affiliations:** 1 Department of Radiotherapy and Radiation Oncology, Jena University Hospital, 07747 Jena, Germanyklaus.pietschmann@med.uni-jena.de (K.P.);; 2 Comprehensive Cancer Center Central Germany, Partner Site Jena, 07747 Jena, Germany

## Text Correction

There was an error in the original publication [1]. The total number of search results was reported to be 15,551, but the correct number is 15,553. A correction has been made to the Results section of the Abstract and Paragraph 1 of the Results section.

Results: From all (15,553) search results, 52 publications concerning 28 RCTs reporting on approximately 7000 patients were included. The therapeutic concepts in the experimental group included all combinations of RT with concurrent systemic therapy, excluding cisplatin-based regimens, and were compared with any control treatment. All studies had sufficient quality for inclusion. None of the systemic agents showed a significant improvement in OS, PFS, or LC compared to cisplatin. Carboplatin + 5-FU and mitomycin C + 5-FU improved LC compared to hyperfractionated accelerated radiotherapy (HART). Compared to cisplatin, durvalumab and cetuximab were associated with less dysphagia, cetuximab with less renal impairment, and panitumumab and cetuximab with less weight loss.

The systematic search revealed 15,553 results. An additional 15 studies were added by hand search. At first, duplicates were removed, leaving 13,197 studies. After screening the title and abstract, 99 studies remained, which were retrieved to complete full-text screening. Finally, 52 publications of 28 studies were included in this NMA, but only 26 could be analyzed, because two studies (Nagpal et al. [14] and Argiris et al. [15]) missed a connection to the network.

## Error in Figure

In the original publication [1], there was a mistake in Figure 1 as published. The number of search results was reported to be 15,551, but the correct number is 15,553. The corrected version of Figure 1 is presented below.


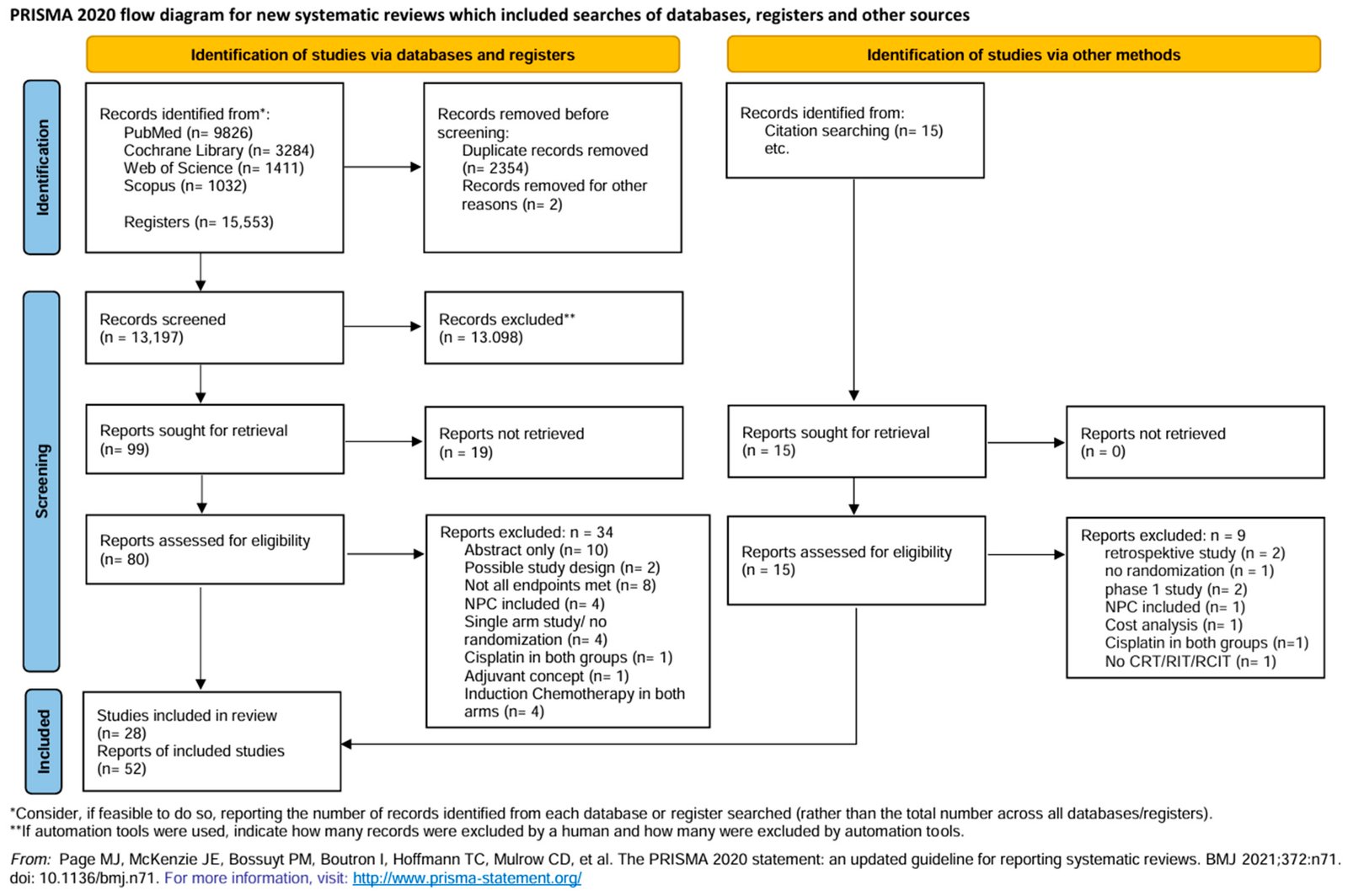


The authors state that the scientific conclusions are unaffected. This correction was approved by the Academic Editor. The original publication has also been updated.
