# Supplementary material for: Non-Cisplatin Concurrent Systemic Therapy with Radiotherapy for Locally Advanced Head and Neck Squamous Cell Carcinoma: A Network Meta-Analysis of Randomized Clinical Trials
Source: Cancers (Basel). 2026 May 14;18(10):1599. doi: 10.3390/cancers18101599 (PMC13204043; doi:10.3390/cancers18101599)
Supplement: Supplementary file 1 [file cancers-18-01599-s001.zip › cancers-4313239-supplementary/Supplementary material 1.pdf]

NIHR | National Institute for Health and Care Research

PROSPERO
International prospective register of systematic reviews

Assessment of efficacy and toxicity of second-line therapeutics used in chemoradiotherapy for cisplatin-inleigible patients with head and neck cancers - a network meta-analysis of randomized clinical trials

Katharina Sophie Schöbel, Anne-Josephin Schoele, Georg Wurschi, Klaus Pietschmann, Maximilian Römer

To enable PROSPERO to focus on COVID-19 submissions, this registration record has undergone basic automated checks for eligibility and is published exactly as submitted. PROSPERO has never provided peer review, and usual checking by the PROSPERO team does not endorse content. Therefore, automatically published records should be treated as any other PROSPERO registration. Further detail is provided here.

Citation 1 change
Katharina Sophie Schöbel, Anne-Josephin Schoele, Georg Wurschi, Klaus Pietschmann, Maximilian Römer. Assessment of efficacy and toxicity of second-line therapeutics used in chemoradiotherapy for cisplatin-inleigible patients with head and neck cancers - a network meta-analysis of randomized clinical trials. PROSPERO 2024 CRD42024578276. Available from https://www.crd.york.ac.uk/PROSPERO/view/CRD42024578276.

REVIEW TITLE AND BASIC DETAILS

Review title 1 change
Assessment of efficacy and toxicity of second-line therapeutics used in chemoradiotherapy for cisplatin-inleigible patients with head and neck cancers - a network meta-analysis of randomized clinical trials

Original language title 1 change
Vergleich der Wirksamkeit und Risiken von Radiochemotherapie mit second-line Therapeutika bei cisplatin-unfitten Patienten mit HNO-Tumoren - eine Netwerkmetaanalyse von randomisiert-kontrollierten Studien

Review objectives 1 change
To compare different concurrent second-line therapeutics for radiochemotherapy/

radioimmunotherapy for locally advanced SCCHN, used when patients are cisplatin-ineligible, this network meta-analysis compares overall survival, progression/disease free survival and toxicity and adverse events

Keywords
carboplatin, cetuximab, head and neck cancer, radiochemotherapy, squamous cell carcinoma, taxanes

SEARCHING AND SCREENING

Searches 1 change
- PubMed, Web of Science, Scopus, Cochrane Library- English and German- Literature published 2005 or later

Study design 1 change
Only randomized study types will be included.

Included
- Inclusion: RCT

Excluded
- Exclusion: single arm study, prospective/ retrospective study, study without randomization

Link to search strategy
A full search strategy is available in the full protocol as described in the *Availability of full protocol* section

ELIGIBILITY CRITERIA

Condition or domain being studied 1 change
Squamous Cell Carcinoma Of Head And Neck

Squamous cell carcinoma of the head and neck

Population 1 change

Included
- Inclusion: adult patients with SCCHN

Excluded
- Exclusion: nasopharyngeal carcinoma, carcinoma in situ, precancerous conditions, preclinical studies

Intervention(s) or exposure(s) 1 change

Included
Radiotherapy; Chemoradiotherapy

- Inclusion: Radiochemotherapy, radioimmunotherapy, radiochemoimmunotherapy with active agents other than cisplatin, curative approach

Excluded

- Exclusion: radiochemotherapy with only cisplatin as an active agent, palliative approach

Comparator(s) or control(s) 1 change

- Inclusion: All possible control groups (active control, placebo, standard/ guideline/ usual care)

Context 1 change

Aim of this network meta-analysis is to compare patients with LA-HNSCC treated with definitive chemoradiotherapy/immunoradiotherapy/chemoimmunoradiotherapy with active agents other than cisplatin to all possible control groups regarding overall survival, progression-free survival, locoregional control and adverse events.

OUTCOMES TO BE ANALYSED

Main outcomes 1 change

Main outcomes will be overall survival, progression free interval and locoregional control

Additional outcomes 1 change

Additional outcomes will be toxicity and adverse events

DATA COLLECTION PROCESS

Data extraction (selection and coding) 1 change

A PRISMA-chart will be created.All found studies will be imported into the data management programm EndNote 20.Selection of studies will be carried out by two people individually.Dublicates will be removed, titles and abstracts will be screened, in case of uncertainty the full text will be acquired.Results of screening will be discussed, any disagreements will be resolved by involving a third person. If data is unclear or missing authors can be contacted for further information.Data extraction will preferably carried out by two reviewers individually. - information about study design will be extracted, including methods of assessment and duration of the trial- population will be described using age, sex, population size and eligibility criteria of their study- intervention will be described using fractionation scheme, applied chemo/immuotherapy and dosage- control intervention will be described using fractionation scheme, applied chemo/immuotherapy and dosage- overall survival, progression/disease free interval, toxicity and adverse events will be noted

Risk of bias (quality) assessment 1 change

Risk of Bias (RoB) for the randomized trials will be assessed by two independent reviewers using the Scottish Intercollegiate Guidelines Network (SIGN) Methodology Checklist 2: Controlled Trials. In case of disagreement consensus will be made by discussion.Every source will be graded according to the Oxford Centre 2011 Levels of Evidence and a short explanation for each judgement will be noted.Consideration is given to possible conflict of interest, a pre-defined study protocol and power-analysis.Publication bias will be assessed through funnel plots for every endpoint including ≥10 studies.

PLANNED DATA SYNTHESIS

Strategy for data synthesis 1 change

A meta-analysis is carried out to compare cisplatin-free intervention with control group.Outcomes (Overall survival, Progression free interval, locoregional control) will be compared using HR. If HR are not reported, they will be estimated from their Kaplan-Meier curves using digitizeIt and Excel spreadsheet (version 3.0) developed by Matthew Sydes and Jayne Tierney.

Secondary endpoints (Toxicity and adverse events) will be compared using OR. If OR are not reported, they will be calculated from their reported percentages.

R Studio will be used for statistical analysis with the help of a professional statistician. Heterogenity will be analysed using I^2. A frequentist approach with a random effects model will be used for this network meta-analysis.

Analysis of subgroups or subsets

None

REVIEW AFFILIATION, FUNDING AND PEER REVIEW

Review team members 1 change

Ms Katharina Sophie Schöbel (review guarantor and contact) Friedrich-Schiller-University Jena. Germany.

No conflict of interest declared.

Anne-Josephin Schoele. Friedrich-Schiller-Universität Jena. Germany.

No conflict of interest declared.

Georg Wurschi. Universitätsklinikum Jena. Germany.

No conflict of interest declared.

Dr Klaus Pietschmann. Universitätsklinikum Jena. Germany.

No conflict of interest declared.

Dr Maximilian Römer. Universitätsklinikum Jena. Germany.

No conflict of interest declared.

Named contact

Ms Katharina Sophie Schöbel (katharina.sophie.schoebel@uni-jena.de). Friedrich-Schiller-University Jena. Germany.

Review affiliation

Friedrich-Schiller-University Jena

Funding source

none

TIMELINE OF THE REVIEW

Review timeline

Start date: 1 August 2024. End date: 1 January 2026.

Date of first submission to PROSPERO
14 August 2024

Date of registration in PROSPERO
27 August 2024

AVAILABILITY OF FULL PROTOCOL

Availability of full protocol
A full protocol has been written and uploaded to PROSPERO. The protocol may be accessed through this link https://www.crd.york.ac.uk/PROSPEROFILES/eed623f55106dd9496f08aebd0a4b0ec.pdf.

CURRENT REVIEW STAGE

Publication of review results
Results of the review will be published in English.

Journal publication
Not yet published in a journal but will be in future.

Stage of the review at this submission

Review stage	Started	Completed
Pilot work	✓	✓
Formal searching/study identification	✓	✓
Screening search results against inclusion criteria	✓	✓
Data extraction or receipt of IPD	✓	✓
Risk of bias/quality assessment	✓	✓
Data synthesis	✓	✓

Review status
The review is completed.

ADDITIONAL INFORMATION

Additional information 1 change
Many reviews focus on specific alternative treatment options and compare them to standard treatment with RT and concurrent cisplatin, but there is a lack of an overall analysis comparing all second-line therapeutics for radiochemotherapy in case a patient is cisplatin-ineligible.

PROSPERO version history 1 change

- Version 2.0, published 01 Feb 2026

- Version 1.2, published 01 Feb 2026
- Version 1.1, published 27 Aug 2024
- Version 1.0, published 27 Aug 2024

Review conflict of interest
None known

Country
Germany

Medical Subject Headings
Chemoradiotherapy; Cisplatin; Disease-Free Survival; Head and Neck Neoplasms; Humans; Radioimmunotherapy

Revision note 1 change
The list of team members of this network meta-analysis was updated. Methods for assessing risk of bias and data analysis were described more detailed.

Minor changes were made such as: correction of spelling errors, moving exclusion and inclusion criteria into their respective boxes for better readability.

Disclaimer
The content of this record displays the information provided by the review team. PROSPERO does not peer review registration records or endorse their content.

PROSPERO accepts and posts the information provided in good faith; responsibility for record content rests with the review team. The guarantor for this record has affirmed that the information provided is truthful and that they understand that deliberate provision of inaccurate information may be construed as scientific misconduct.

PROSPERO does not accept any liability for the content provided in this record or for its use. Readers use the information provided in this record at their own risk.

Any enquiries about the record should be referred to the named review contact
