# Supplementary material for: Non-Cisplatin Concurrent Systemic Therapy with Radiotherapy for Locally Advanced Head and Neck Squamous Cell Carcinoma: A Network Meta-Analysis of Randomized Clinical Trials
Source: Cancers (Basel). 2026 May 14;18(10):1599. doi: 10.3390/cancers18101599 (PMC13204043; doi:10.3390/cancers18101599)
Supplement: Supplementary file 1 [file cancers-18-01599-s001.zip › cancers-4313239-supplementary/Supplementary material 10.pdf]

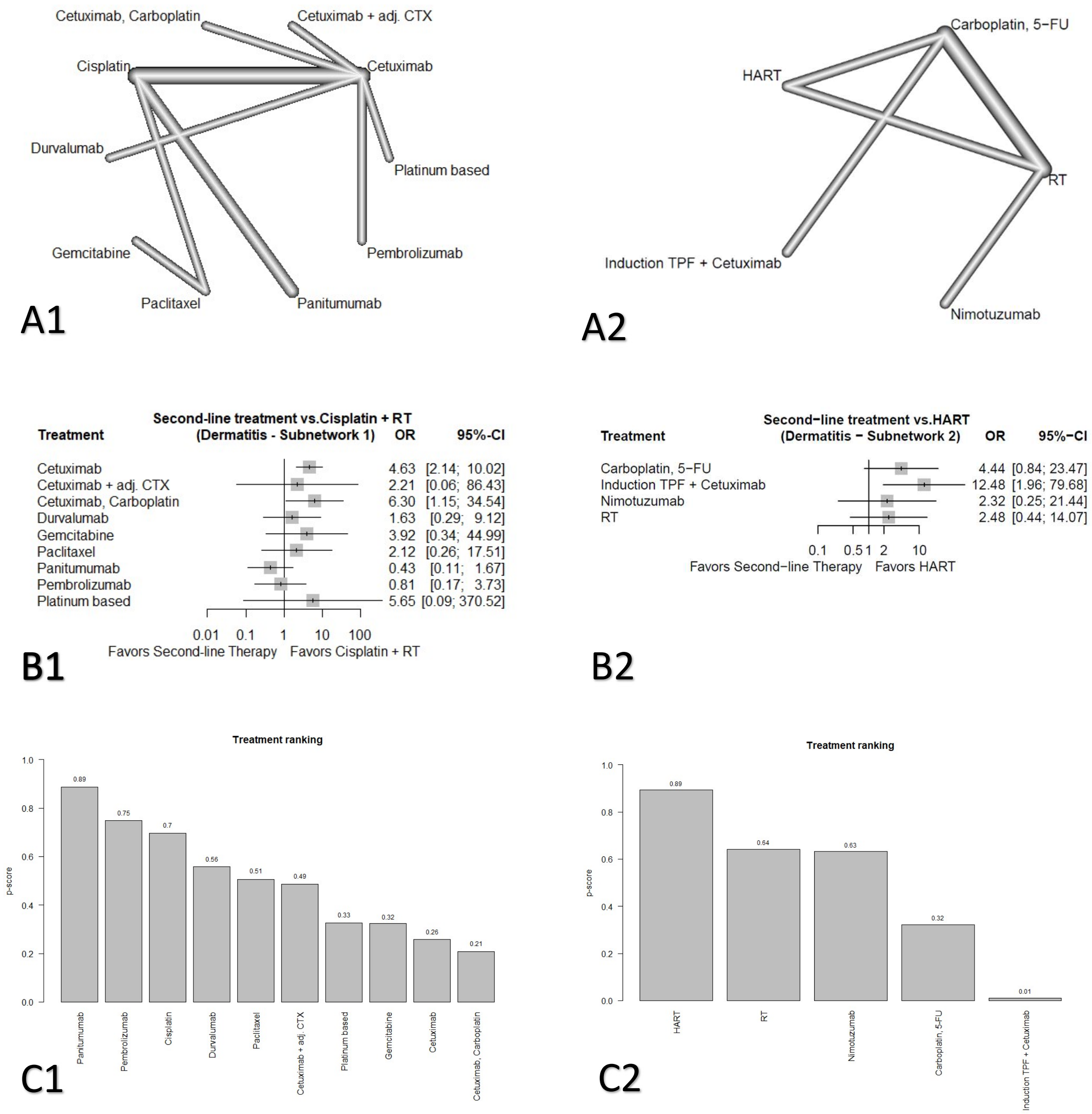


*Supplementary material 9: Results for dermatitis (**A1**) netgraph subnetwork 1, (**A2**) netgraph subnetwork 2, (**B1**) forest plot subnetwork 1 , (**B2**) forest plot subnetwork 2, (**C1**) treatment ranking subnetwork 1, (**C2**) treatment ranking subnetwork 2*
