# Supplementary material for: Non-Cisplatin Concurrent Systemic Therapy with Radiotherapy for Locally Advanced Head and Neck Squamous Cell Carcinoma: A Network Meta-Analysis of Randomized Clinical Trials
Source: Cancers (Basel). 2026 May 14;18(10):1599. doi: 10.3390/cancers18101599 (PMC13204043; doi:10.3390/cancers18101599)
Supplement: Supplementary file 1 [file cancers-18-01599-s001.zip › cancers-4313239-supplementary/Supplementary material 2.pdf]

*Supplementary material 2: Search strings used in network meta-analysis (**A**) Search string for Medline, (**B**) Search string for Web of Science, (**C**) Search string for Cochrane Library, (**D**) Search string for Scopus*

A) <u>Search string for Medline:</u>

(“laryngeal neoplasms”[Mesh] OR “pharyngeal neoplasms”[Mesh] OR “tracheal neoplasms”[Mesh] OR „squamous cell carcinoma of head and neck“[Mesh] OR „head and neck neoplasms“[Mesh] OR „SCCHN“[tw] OR „HNSC“[tw] OR „squamous cell head and neck tumor“[tiab:~0] OR „SCC of head and neck“[tiab:~0] OR „head and neck cancer of squamous origin“[tiab:~0] OR „pharyngeal cancer*“[tw] OR „LASCCHN“[tw] OR „oropharyngeal cancer*“[tw] OR „squamous carcinoma*“[tw] OR „craniocervical region squamous cell carcinoma“[tiab:~0] OR „cervical squamous cell carcinoma*“[tw] OR „squamous cell carcinoma* of head and neck“[tw] OR „somatic squamous cell carcinoma* head and neck“[tiab:~0] OR „head and neck epithelial cancer“[tiab:~0] OR „HNC“[tw] OR „oral and pharyngeal cancer*“[tw] OR „oropharynx cancer*“[tw] OR „oral cavity cancer*“[tw] OR „pharynx cancer*“[tw] OR „larynx cancer*“[tw] OR „malignant head and neck neoplasm*“[tw] OR „head and neck cancer NOS“[tiab:~0] OR „malignant head and neck tumor*“[tw] OR „malignant neoplasm* of head and neck“[tw] OR „malignant neoplasm of face head and neck“[tiab:~0] OR „malignant neoplasm* of the head and neck“[tw] OR „malignant tumor* of head and neck“[tw] OR „malignant tumor* of the head and neck“[tw] OR „head and neck tumor*“[tw] OR „craniofacial neoplasm*“[tw] OR „cervicofacial neoplasm*“[tw] OR „cervicofacial tumor*“[tw] OR „craniofacial tumor*“[tw] OR „cervicofacial cancer*“[tw] OR „orofacial neoplasm*“[tw] OR „head and neck malignan*“[tw] OR „craniofacial malignan*“[tw] OR „cervicofacial malignancy“[tiab:~0] OR „orofacial tumor*“[tw] OR „head and neck growth“[tiab:~0] OR „craniofacial growth“[tw] OR „cervicofacial growth“[tiab:~0] OR „orofacial cancer*“[tw] OR „orofacial malignan*“[tw] OR „orofacial growth“[tw]) AND (“radiochemotherap*”[tw] OR “radiation*”[tw] OR “adjuvant”[tw] OR “chemoradiotherap*”[tw] OR “neo-adjuvant”[tw] OR “fraction”[tw] OR “radiotherap*”[tw] OR „chemoradiotherapy“[Mesh] OR „CRT“[tw] OR „CRTx“[tw] OR „CT-RT“[tw] OR „RCT“[tw] OR „RCTx“[tw] OR „RT-CT“[tw] OR „chemoradiat*“[tw] OR „adjuvant chemoradi*“[tw] OR „adjuvant radi*“[tw] OR „gray“[tw] OR „Gy“[tw]) AND (((„cisplatin“[Mesh] OR „cisplatinum“[tw] OR „neoplatin“[tw] OR „cismaplat“[tw] OR „CDDP“[tw] OR „CPDC“[tw] OR „DDP“[tw]) AND („contraindications“[Mesh] OR „ineligib*“[tw] OR „unfit*“[tw] OR „contra?indicat*“[tw] OR „allerg*“[tw] OR „hypersensitiv*“[tw] OR „intoleran*“[tw] OR „vulnerab*“[tw] OR „oversensitiv*“[tw] OR „adverse condition*“[tw] OR „poor condition*“[tw] OR „unqualif*“[tw] OR „unsuitab*“[tw] OR „no* suitab*“[tiab:~0] OR „no* eligib*“[tiab:~0] OR „no* fit“[tw] OR „no* qualif*“[tiab:~0] OR „rule* out“[tw] OR „disqualif*“[tw] OR „inappropriate“[tw] OR „exclu*“[tw] OR „in?pt“[tw])) OR („carboplatin“[Mesh] OR „cetuximab“[Mesh] OR „paclitaxel“[Mesh] OR „docetaxel“[Mesh] OR „etoposide“[Mesh] OR „fluorouracil“[Mesh] OR „hydroxyurea“[Mesh] OR „cyclophosphamide“[Mesh] OR „doxorubicin“[Mesh] OR „vincristine“[Mesh] OR „CEV regimen“[Supplementary Concept] OR „platinum?based“[tw] OR „41575-94-4”[tw] OR “Platinum, diammine(1,1-cyclobutanedicarboxylato(2-))-, (SP-4-2)-“[tw] OR „Cétuximab“[tw] OR „Chimeric Anti-EGFR Monoclonal Antibody“[tw] OR „Chimeric MoAb C225“[tiab:~0] OR „Chimeric Monoclonal Antibody C225“[tw] OR „EGFR inhibitor“[tw] OR „targeted therap*“[tw] OR „immunotherap*“[tw] OR „immune checkpoint inhibitor*“[tw] OR „33069-62-4“[tw] OR „taxane?based“[tw] OR „taxane*“[tw] OR „taxol“[tw] OR „mitotic inhibit*“[tw] OR „abraxane“[tw] OR „onxol“[tw] OR „PTX“[tw] OR „taxotere“[tw] OR „trihydrate“[tw] OR „DTX“[tw] OR „DXL“[tw] OR „VP-16-213“[tw] OR

„celltop“[tw] OR „eposide“[tw] OR „VP-16“[tw] OR „5-Fluor-2,4(1H,3H)-pyrimidindion“[tw] OR „Carbamohydroxamic acid“[tw] OR „N-Carbamoylhydroxylamine“[tw] OR „N-Hydroxyurea“[tw] OR „Biosupressin“[tw] OR „Carbamohydroxamic Acid“[tw] OR „Carbamoyl Oxime“[tw] OR „Droxia“[tw] OR „HU“[tw] OR „Hidrix“[tw] OR „hydroxylurea“[tw] OR „Hydura“[tw] OR „Hydurea“[tw] OR „N-Carbamoylhydroxylamine“[tw] OR „NCI C04831“[tw] OR „NSC?32065“[tw] OR „Oxyurea“[tw] OR „SK 22591“[tw] OR „SQ 1089“[tw] OR „127-07-1“[tw] OR „CEV“[tw] OR „VEJ“[tw] OR „Cyclophosphamide-Etoposide-Vincristine“[tw] OR „ADR“[tw] OR „2 Dox“[tw] OR „hydroxydaunorubicin“[tw] OR „hydroxydaunomycin“[tw] OR „Cyclo“[tw] OR „CPA“[tw] OR „CPM“[tw] OR „CTX“[tw] OR „CYC“[tw] OR „CYT“[tw] OR „Neosar“[tw] OR „Cytoxan“[tw] OR „Procytox“[tw] OR „Citomid“[tw] OR „VCR“[tw] OR „22-Oxovincaleukoblastine“[tw]))

B) Search string for Web of Science:

(KP=(laryngeal neoplasm*) OR KP=(larynx neoplasm*) OR KP=(cancer* of larynx) OR KP=(larynx cancer*) OR KP=(laryngeal cancer*) OR KP=(cancer* of the larynx) OR KP=(pharyngeal neoplasm*) OR KP=(pharynx neoplasm*) OR KP=(cancer* of pharynx) OR KP=(pharynx cancer*) OR KP=(pharyngeal cancer*) OR KP=(cancer* of the pharynx) OR KP=(tracheal neoplasm*) OR KP=(neoplasm* of trachea) OR KP=(neoplasm* of the trachea) OR KP=(cancer* of trachea) OR KP=(tracheal cancer*) OR KP=(squamous cell carcinoma* of head and neck) OR KP=(head and neck squamous cell carcinoma*) OR KP=(squamous cell carcinoma* of the head and neck) OR KP=(HNSCC) OR KP=(squamous cell carcinoma* of the larynx) OR KP=(laryngeal squamous cell carcinoma*) OR KP=(squamous cell carcinoma* of larynx) OR KP=(squamous cell carcinoma of the nasal cavity) OR KP=(oral tongue squamous cell carcinoma*) OR KP=(hypopharyngeal squamous cell carcinoma*) OR KP=(oral squamous cell carcinoma*) OR KP=(oral cavity squamous cell carcinoma*) OR KP=(squamous cell carcinoma* of the mouth) OR KP=(oropharyngeal squamous cell carcinoma*) OR KP=(head and neck neoplasm*) OR KP=(cancer* of head and neck) OR KP=(cancer* of the head and neck) OR KP=(upper aerodigestive tract neoplasm*) OR KP=(UADT neoplasm*) OR KP=(head neoplasm*) OR KP=(neck neoplasm*) OR KP=(cancer* of head) OR KP=(head cancer*) OR KP=(cancer* of the head) OR KP=(cancer* of neck) OR KP=(neck cancer*) OR KP=(cancer* of the neck) OR KP=(SCCHN) OR KP=(HNSC) OR KP=(squamous cell head and neck tumor) OR KP=(SCC of head and neck) OR KP=(head and neck cancer of squamous origin) OR KP=(pharyngeal cancer*) OR KP=(LASCCHN) OR KP=(oropharyngeal cancer*) OR KP=(squamous carcinoma*) OR KP=(craniocervical region squamous cell carcinoma) OR KP=(cervical squamous cell carcinoma*) OR KP=(squamous cell carcinoma* of head and neck) OR KP=(somatic squamous cell carcinoma* head and neck) OR KP=(head and neck epithelial cancer) OR KP=(HNC) OR KP=(oral and pharyngeal cancer*) OR KP=(oropharynx cancer*) OR KP=(oral cavity cancer*) OR KP=(pharynx cancer*) OR KP=(larynx cancer*) OR KP=(malignant head and neck neoplasm*) OR KP=(head and neck cancer NOS) OR KP=(malignant head and neck tumor*) OR KP=(malignant neoplasm* of head and neck) OR KP=(malignant neoplasm of face head and neck) OR KP=(malignant neoplasm* of the head and neck) OR KP=(malignant tumor* of head and neck) OR KP=(malignant tumor* of the head and neck) OR KP=(head and neck tumor*) OR KP=(craniofacial neoplasm*) OR KP=(cervicofacial neoplasm*) OR KP=(cervicofacial tumor*) OR KP=(craniofacial tumor*) OR KP=(cervicofacial cancer*) OR KP=(orofacial neoplasm*) OR KP=(head and neck malignan*) OR KP=(craniofacial malignan*) OR KP=(cervicofacial malignancy) OR KP=(orofacial tumor*) OR KP=(head and neck growth) OR KP=(craniofacial growth) OR KP=(cervicofacial growth) OR KP=(orofacial cancer*) OR KP=(orofacial malignan*) OR KP=(orofacial growth)) AND

(KP=(radiochemotherap*) OR KP=(radiation*) OR KP=(adjuvant) OR KP=(chemoradiat*) OR KP=(chemoradiotherap*) OR KP=(neo-adjuvant) OR KP=(fraction) OR KP=(radiotherap*) OR KP=(CRT) OR KP=(CRTx) OR KP=(CT-RT) OR KP=(RCT) OR KP=(RCTx) OR KP=(RT-CT) OR KP=(chemoradiat*) OR KP=(adjuvant chemoradi*) OR KP=(adjuvant radi*) OR KP=(gray) OR KP=(Gy)) AND (((TS=(cisplatin) OR TS=(cis-Diamminedichloroplatinum(II)) OR TS=(Platinum Diamminodichloride) OR TS=(cis?Platinum) OR TS=(Dichlorodiammineplatinum) OR TS=(cis?Diamminedichloroplatinum) OR TS=(cis-Dichlorodiammineplatinum(II)) OR TS=(NSC-119875) OR TS=(Platino) OR TS=(Platinol) OR TS=(Biocisplatinum) OR TS=(Platidiam) OR TS=(cisplatinum) OR TS=(neoplatin) OR TS=(cismaplat) OR TS=(CDDP) OR TS=(CPDC) OR TS=(DDP)) AND (TS=(contraindication*) OR TS=(ineligib*) OR TS=(unfit*) OR TS=(contra?indicat*) OR TS=(allerg*) OR TS=(hypersensitiv*) OR TS=(intoleran*) OR TS=(vulnerab*) OR TS=(oversensitiv*) OR TS=(adverse condition*) OR TS=(poor condition*) OR TS=(unqualif*) OR TS=(unsuitab*) OR TS=(non suitab*) OR TS=(non eligib*) OR TS=(non fit) OR TS=(non qualif*) OR TS=(rule* out) OR TS=(disqualif*) OR TS=(inappropriate) OR TS=(exclu*) OR TS=(in?pt))) OR (AK=(carboplatin) OR AK=(cis-Diammine(cyclobutanedicarboxylato)platinum II) OR AK=(CBDCA) OR AK=(Paraplatin*) OR AK=(Platinwas) OR AK=(Ribocarbo) OR AK=(Carboplat) OR AK=(Neocarbo) OR AK=(Carbosin) OR AK=(Carbotec) OR AK=(Ercar) OR AK=(JM?8) OR AK=(JM8) OR AK=(Nealorin) OR AK=(NSC?241240) OR AK=(NSC241240) OR AK=(Blastocarb) OR AK=(cetuximab) OR AK=(Erbitux) OR AK=(IMC?C225) OR AK=(MAb C225) OR AK=(C225) OR AK=(paclitaxel) OR AK=(Anzatax) OR AK=(NSC?125973) OR AK=(NSC125973) OR AK=(Taxol) OR AK=(Taxol A) OR AK=(Bris Taxol) OR AK=(Paxene) OR AK=(Paclitaxel, (4 alpha)-Isomer) OR AK=(Praxel) OR AK=(7?epi?Taxol) OR AK=(Onxol) OR AK=(docetaxel) OR AK=(Docetaxol) OR AK=(Docetaxel Trihydrate) OR AK=(Docetaxel Hydrate) OR AK=(Taxoltere Metro) OR AK=(RP?56976) OR AK=(RP56976) OR AK=(Taxotere) OR AK=(Docetaxel Anhydrous) OR AK=(N-Debenzoyl-N-tert-butoxycarbonyl-10-deacetyltaxol) OR AK=(N Debenzoyl N tert butoxycarbonyl 10 deacetyltaxol) OR AK=(NSC 628503) OR AK=(etoposide) OR AK=(Eposide) OR AK=(Demethyl Epipodophyllotoxin Ethylidine Glucoside) OR AK=(Etoposide, (5a alpha,9 alpha)-Isomer) OR AK=(Etoposide, (5S)-Isomer) OR AK=(Etoposide, alpha-D-Glucopyranosyl Isomer) OR AK=(Etoposide, alpha D Glucopyranosyl Isomer) OR AK=(alpha-D-Glucopyranosyl Isomer Etoposide) OR AK=(Etoposido Ferrer Farma) OR AK=(Exitop) OR AK=(Lastet) OR AK=(NSC?141540) OR AK=(NSC141540) OR AK=(Onkoposid) OR AK=(Riboposid) OR AK=(Toposar) OR AK=(Etoposide Teva) OR AK=(Vepesid) OR AK=(VP?16?213) OR AK=(VP 16213) OR AK=(VP?16) OR AK=(VP16) OR AK=(Vépéside?Sandoz) OR AK=(Celltop) OR AK=(Etopos) OR AK=(Etomedac) OR AK=(Eposin) OR AK=(Etoposide, (5a alpha)-Isomer) OR AK=(Etoposide Pierre Fabre) OR AK=(fluorouracil) OR AK=(5FU) OR AK=(5?FU) OR AK=(5?Fluorouracil) OR AK=(Fluoruracil) OR AK=(Adrucil) OR AK=(Carac) OR AK=(Efudix) OR AK=(Fluoro?Uracile) OR AK=(Efudex) OR AK=(Fluoroplex) OR AK=(Flurodex) OR AK=(Fluorouracil?GRY) OR AK=(Fluorouracile Dakota) OR AK=(Fluorouracilo Ferrer Far) OR AK=(Fluracedyl) OR AK=(Haemato?FU) OR AK=(Neofluor) OR AK=(Onkofluor) OR AK=(Ribofluor) OR AK=(5?Fluorouracil?Biosyn) OR AK=(hydroxyurea) OR AK=(Hydroxycarbamid) OR AK=(Oncocarbide) OR AK=(Hydrea) OR AK=(cyclophosphamid*) OR AK=(Sendoxan) OR AK=(B?518) OR AK=(B518) OR AK=(cytophosphan*) OR AK=(Cytoxan) OR AK=(Endoxan) OR AK=(Neosar) OR AK=(NSC?26271) OR AK=(NSC26271) OR AK=(Procytox) OR AK=(Cyclophosphane) OR AK=(doxorubicin) OR AK=(Farmiblastina) OR AK=(Ribodoxo) OR AK=(Rubex) OR AK=(Adriamycin) OR AK=(Adriblastin*) OR AK=(Adriablastin*) OR AK=(Adrimedac) OR AK=(DOXO?cell) OR AK=(Urokit Doxo?cell) OR AK=(Doxolem) OR AK=(Doxorubicin Hydrochloride) OR AK=(Doxotec) OR AK=(Myocet) OR AK=(Onkodox) OR AK=(vincristin*) OR AK=(Leurocristin*) OR AK=(cellcristin) OR AK=(Citomid) OR AK=(Oncovin*) OR AK=(Onkocristin) OR AK=(Vincasar) OR AK=(Vintec) OR AK=(Vincrisul) OR AK=(VEC

regimen) OR AK=(CEV regimen) OR AK=(VEJ regimen) OR AK=(platinum?based) OR AK=(41575?94?4) OR AK=(C?tuximab) OR AK=(Chimeric Anti-EGFR Monoclonal Antibody) OR AK=(Chimeric MoAb C225) OR AK=(Chimeric Monoclonal Antibody C225) OR AK=(EGFR inhibitor) OR AK=(targeted therap*) OR AK=(immunotherap*) OR AK=(immune checkpoint inhibitor*) OR AK=(33069?62?4) OR AK=(taxane?based) OR AK=(taxane*) OR AK=(taxol) OR AK=(mitotic inhibit*) OR AK=(abraxane) OR AK=(onxol) OR AK=(PTX) OR AK=(taxotere) OR AK=(trihydrate) OR AK=(DTX) OR AK=(DXL) OR AK=(VP?16?213) OR AK=(celltop) OR AK=(eposide) OR AK=(VP-16) OR AK=(Carbamohydroxamic acid) OR AK=(N-Carbamoylhydroxylamine) OR AK=(N-Hydroxyurea) OR AK=(Biosupressin) OR AK=(Carbamohydroxamic Acid) OR AK=(Carbamoyl Oxime) OR AK=(Droxia) OR AK=(HU) OR AK=(Hidrix) OR AK=(hydroxylurea) OR AK=(Hydura) OR AK=(Hydurea) OR AK=(N-Carbamoylhydroxylamine) OR AK=(NCI C04831) OR AK=(NSC?32065) OR AK=(Oxyurea) OR AK=(SK 22591) OR AK=(SQ 1089) OR AK=(127?07?1) OR AK=(CEV) OR AK=(VEJ) OR AK=(Cyclophosphamide-Etoposide-Vincristine) OR AK=(ADR) OR AK=(2 Dox) OR AK=(hydroxydaunorubicin) OR AK=(hydroxydaunomycin) OR AK=(Cyclo) OR AK=(CPA) OR AK=(CPM) OR AK=(CTX) OR AK=(CYC) OR AK=(CYT) OR AK=(Neosar) OR AK=(Cytoxan) OR AK=(Procytox) OR AK=(Citomid) OR AK=(VCR) OR AK=(22?Oxovincaleukoblastine)))

C) Search string for Cochrane Library:

([mh laryngeal neoplasms] OR [mh pharyngeal neoplasms] OR [mh tracheal neoplasms] OR [mh squamous cell carcinoma of head and neck] OR [mh head and neck neoplasms] OR (SCCHN) OR (HNSC) OR (squamous cell head and neck tumor) OR (SCC of head and neck) OR (head and neck cancer of squamous origin) OR (pharyngeal cancer*) OR (LASCCHN) OR (oropharyngeal cancer*) OR (squamous carcinoma*) OR (craniocervical region squamous cell carcinoma) OR (cervical squamous cell carcinoma*) OR (squamous cell carcinoma* of head and neck) OR (somatic squamous cell carcinoma* head and neck) OR (head and neck epithelial cancer) OR (HNC) OR (oral and pharyngeal cancer*) OR (oropharynx cancer*) OR (oral cavity cancer*) OR (pharynx cancer*) OR (larynx cancer*) OR (malignant head and neck neoplasm*) OR (head and neck cancer NOS) OR (malignant head and neck tumor*) OR (malignant neoplasm* of head and neck) OR (malignant neoplasm of face head and neck) OR (malignant neoplasm* of the head and neck) OR (malignant tumor* of head and neck) OR (malignant tumor* of the head and neck) OR (head and neck tumor*) OR (craniofacial neoplasm*) OR (cervicofacial neoplasm*) OR (cervicofacial tumor*) OR (craniofacial tumor*) OR (cervicofacial cancer*) OR (orofacial neoplasm*) OR (head and neck malignan*) OR (craniofacial malignan*) OR (cervicofacial malignancy) OR (orofacial tumor*) OR (head and neck growth) OR (craniofacial growth) OR (cervicofacial growth) OR (orofacial cancer*) OR (orofacial malignan*) OR (orofacial growth)) AND ([mh chemoradiotherapy] OR (radiochemotherap*) OR (radiation*) OR (adjuvant) OR (chemoradiat*) OR (chemoradiotherap*) OR (neo-adjuvant) OR (fraction) OR (radiotherap*) OR (CRT) OR (CRTx) OR (CT-RT) OR (RCT) OR (RCTx) OR (RT-CT) OR (chemoradiat*) OR (adjuvant chemoradi*) OR (adjuvant radi*) OR (gray) OR (Gy)) AND ((([mh cisplatin] OR (cisplatinum) OR (neoplatin) OR (cismaplat) OR (CDDP) OR (CPDC) OR (DDP)) AND ([mh contraindications] OR (ineligib*) OR (unfit*) OR (contra?indicat*) OR (allerg*) OR (hypersensitiv*) OR (intoleran*) OR (vulnerab*) OR (oversensitiv*) OR (adverse condition*) OR (poor condition*) OR (unqualif*) OR (unsuitab*) OR (no* suitab*) OR (no* eligib*) OR (no* fit) OR (no* qualif*) OR (rule* out) OR (disqualif*) OR (inappropriate) OR (exclu*) OR (in?pt))) OR ([mh carboplatin] OR [mh cetuximab] OR [mh paclitaxel] OR [mh docetaxel] OR [mh etoposide] OR [mh flourouracil] OR [mh hydroxyurea] OR [mh cyclophosphamide] OR [mh

doxorubicin] OR [mh vincristine] OR (platinum?based) OR (41575?94?4) OR (C?tuximab) OR (Chimeric Anti-EGFR Monoclonal Antibody) OR (Chimeric MoAb C225) OR (Chimeric Monoclonal Antibody C225) OR (EGFR inhibitor) OR (targeted therap*) OR (immunotherap*) OR (immune checkpoint inhibitor*) OR (33069?62?4) OR (taxane?based) OR (taxane*) OR (taxol) OR (mitotic inhibit*) OR (abraxane) OR (onxol) OR (PTX) OR (taxotere) OR (trihydrate) OR (DTX) OR (DXL) OR (VP?16?213) OR (celltop) OR (eposide) OR (VP-16) OR (Carbamohydroxamic acid) OR (N-Carbamoylhydroxylamine) OR (N-Hydroxyurea) OR (Biosupressin) OR (Carbamohydroxamic Acid) OR (Carbamoyl Oxime) OR (Droxia) OR (HU) OR (Hidrix) OR (hydroxylurea) OR (Hydura) OR (Hydurea) OR (N-Carbamoylhydroxylamine) OR (NCI C04831) OR (NSC?32065) OR (Oxyurea) OR (SK 22591) OR (SQ 1089) OR (127?07?1) OR (CEV) OR (VEJ) OR (Cyclophosphamide-Etoposide-Vincristine) OR (ADR) OR (2 Dox) OR (hydroxydaunorubicin) OR (hydroxydaunomycin) OR (Cyclo) OR (CPA) OR (CPM) OR (CTX) OR (CYC) OR (CYT) OR (Neosar) OR (Cytoxan) OR (Procytox) OR (Citomid) OR (VCR) OR (22?Oxovincaleukoblastine)))

D) <u>Search string for Scopus:</u>

(AUTHKEY(laryngeal neoplasm*) OR AUTHKEY(larynx neoplasm*) OR AUTHKEY(cancer* of larynx) OR AUTHKEY(larynx cancer*) OR AUTHKEY(laryngeal cancer*) OR AUTHKEY(cancer* of the larynx) OR AUTHKEY(pharyngeal neoplasm*) OR AUTHKEY(pharynx neoplasm*) OR AUTHKEY(cancer* of pharynx) OR AUTHKEY(pharynx cancer*) OR AUTHKEY(pharyngeal cancer*) OR AUTHKEY(cancer* of the pharynx) OR AUTHKEY(tracheal neoplasm*) OR AUTHKEY(neoplasm* of trachea) OR AUTHKEY(neoplasm* of the trachea) OR AUTHKEY(cancer* of trachea) OR AUTHKEY(tracheal cancer*) OR AUTHKEY(squamous cell carcinoma* of head and neck) OR AUTHKEY(head and neck squamous cell carcinoma*) OR AUTHKEY(squamous cell carcinoma* of the head and neck) OR AUTHKEY(HNSCC) OR AUTHKEY(squamous cell carcinoma* of the larynx) OR AUTHKEY(laryngeal squamous cell carcinoma*) OR AUTHKEY(squamous cell carcinoma* of larynx) OR AUTHKEY(squamous cell carcinoma of the nasal cavity) OR AUTHKEY(oral tongue squamous cell carcinoma*) OR AUTHKEY(hypopharyngeal squamous cell carcinoma*) OR AUTHKEY(oral squamous cell carcinoma*) OR AUTHKEY(oral cavity squamous cell carcinoma*) OR AUTHKEY(squamous cell carcinoma* of the mouth) OR AUTHKEY(oropharyngeal squamous cell carcinoma*) OR AUTHKEY(head and neck neoplasm*) OR AUTHKEY(cancer* of head and neck) OR AUTHKEY(cancer* of the head and neck) OR AUTHKEY(upper aerodigestive tract neoplasm*) OR AUTHKEY(UADT neoplasm*) OR AUTHKEY(head neoplasm*) OR AUTHKEY(neck neoplasm*) OR AUTHKEY(cancer* of head) OR AUTHKEY(head cancer*) OR AUTHKEY(cancer* of the head) OR AUTHKEY(cancer* of neck) OR AUTHKEY(neck cancer*) OR AUTHKEY(cancer* of the neck) OR AUTHKEY(SCCHN) OR AUTHKEY(HNSC) OR AUTHKEY(squamous cell head and neck tumor) OR AUTHKEY(SCC of head and neck) OR AUTHKEY(head and neck cancer of squamous origin) OR AUTHKEY(pharyngeal cancer*) OR AUTHKEY(LASCCHN) OR AUTHKEY(oropharyngeal cancer*) OR AUTHKEY(squamous carcinoma*) OR AUTHKEY(craniocervical region squamous cell carcinoma) OR AUTHKEY(cervical squamous cell carcinoma*) OR AUTHKEY(squamous cell carcinoma* of head and neck) OR AUTHKEY(somatic squamous cell carcinoma* head and neck) OR AUTHKEY(head and neck epithelial cancer) OR AUTHKEY(HNC) OR AUTHKEY(oral and pharyngeal cancer*) OR AUTHKEY(oropharynx cancer*) OR AUTHKEY(oral cavity cancer*) OR AUTHKEY(pharynx cancer*) OR AUTHKEY(larynx cancer*) OR AUTHKEY(malignant head and neck neoplasm*) OR AUTHKEY(head and neck cancer NOS) OR AUTHKEY(malignant head and neck tumor*) OR AUTHKEY(malignant neoplasm* of head and neck) OR AUTHKEY(malignant neoplasm of face head and neck) OR AUTHKEY(malignant neoplasm* of the head and neck) OR AUTHKEY(malignant tumor* of head and neck) OR AUTHKEY(malignant tumor* of the head and neck) OR AUTHKEY(head and neck tumor*) OR AUTHKEY(craniofacial neoplasm*) OR AUTHKEY(cervicofacial neoplasm*) OR AUTHKEY(cervicofacial tumor*) OR AUTHKEY(craniofacial

tumor*) OR AUTHKEY(cervicofacial cancer*) OR AUTHKEY(orofacial neoplasm*) OR AUTHKEY(head and neck malignan*) OR AUTHKEY(craniofacial malignan*) OR AUTHKEY(cervicofacial malignancy) OR AUTHKEY(orofacial tumor*) OR AUTHKEY(head and neck growth) OR AUTHKEY(craniofacial growth) OR AUTHKEY(cervicofacial growth) OR AUTHKEY(orofacial cancer*) OR AUTHKEY(orofacial malignan*) OR AUTHKEY(orofacial growth)) AND (AUTHKEY(radiochemotherap*) OR AUTHKEY(radiation*) OR AUTHKEY(adjuvant) OR AUTHKEY(chemoradiat*) OR AUTHKEY(chemoradiotherap*) OR AUTHKEY(neo-adjuvant) OR AUTHKEY(fraction) OR AUTHKEY(radiotherap*) OR AUTHKEY(CRT) OR AUTHKEY(CRTx) OR AUTHKEY(CT-RT) OR AUTHKEY(RCT) OR AUTHKEY(RCTx) OR AUTHKEY(RT-CT) OR AUTHKEY(chemoradiat*) OR AUTHKEY(adjuvant chemoradi*) OR AUTHKEY(adjuvant radi*) OR AUTHKEY(gray) OR AUTHKEY(Gy)) AND (((TITLE-ABS(cisplatin) OR TITLE-ABS(Platinum Diamminodichloride) OR TITLE-ABS(cis?Platinum) OR TITLE-ABS(Dichlorodiammineplatinum) OR TITLE-ABS(cis?Diamminedichloroplatinum) OR TITLE-ABS(NSC-119875) OR TITLE-ABS(Platino) OR TITLE-ABS(Platinol) OR TITLE-ABS(Biocisplatinum) OR TITLE-ABS(Platidiam) OR TITLE-ABS(cisplatinum) OR TITLE-ABS(neoplatin) OR TITLE-ABS(cismaplat) OR TITLE-ABS(CDDP) OR TITLE-ABS(CPDC) OR TITLE-ABS(DDP)) AND (TITLE-ABS(contraindication*) OR TITLE-ABS(ineligib*) OR TITLE-ABS(unfit*) OR TITLE-ABS(contra?indicat*) OR TITLE-ABS(allerg*) OR TITLE-ABS(hypersensitiv*) OR TITLE-ABS(intoleran*) OR TITLE-ABS(vulnerab*) OR TITLE-ABS(oversensitiv*) OR TITLE-ABS(adverse condition*) OR TITLE-ABS(poor condition*) OR TITLE-ABS(unqualif*) OR TITLE-ABS(unsuitab*) OR TITLE-ABS(non suitab*) OR TITLE-ABS(non eligib*) OR TITLE-ABS(non fit) OR TITLE-ABS(non qualif*) OR TITLE-ABS(rule* out) OR TITLE-ABS(disqualif*) OR TITLE-ABS(inappropriate) OR TITLE-ABS(exclu*) OR TITLE-ABS(in?pt))) OR (AUTHKEY(carboplatin) OR AUTHKEY(CBDCA) OR AUTHKEY(Paraplatin*) OR AUTHKEY(Platinwas) OR AUTHKEY(Ribocarbo) OR AUTHKEY(Carboplat) OR AUTHKEY(Neocarbo) OR AUTHKEY(Carbosin) OR AUTHKEY(Carbotec) OR AUTHKEY(Ercar) OR AUTHKEY(JM?8) OR AUTHKEY(JM8) OR AUTHKEY(Nealorin) OR AUTHKEY(NSC?241240) OR AUTHKEY(NSC241240) OR AUTHKEY(Blastocarb) OR AUTHKEY(cetuximab) OR AUTHKEY(Erbitux) OR AUTHKEY(IMC?C225) OR AUTHKEY(MAb C225) OR AUTHKEY(C225) OR AUTHKEY(paclitaxel) OR AUTHKEY(Anzatax) OR AUTHKEY(NSC?125973) OR AUTHKEY(NSC125973) OR AUTHKEY(Taxol) OR AUTHKEY(Taxol A) OR AUTHKEY(Bris Taxol) OR AUTHKEY(Paxene) OR AUTHKEY(Praxel) OR AUTHKEY(7?epi?Taxol) OR AUTHKEY(Onxol) OR AUTHKEY(docetaxel) OR AUTHKEY(Docetaxol) OR AUTHKEY(Docetaxel Trihydrate) OR AUTHKEY(Docetaxel Hydrate) OR AUTHKEY(Taxoltere Metro) OR AUTHKEY(RP?56976) OR AUTHKEY(RP56976) OR AUTHKEY(Taxotere) OR AUTHKEY(Docetaxel Anhydrous) OR AUTHKEY(N-Debenzoyl-N-tert-butoxycarbonyl-10-deacetyltaxol) OR AUTHKEY(N Debenzoyl N tert butoxycarbonyl 10 deacetyltaxol) OR AUTHKEY(NSC 628503) OR AUTHKEY(etoposide) OR AUTHKEY(Eposide) OR AUTHKEY(Demethyl Epipodophyllotoxin Ethylidine Glucoside) OR AUTHKEY(Etoposide, alpha-D-Glucopyranosyl Isomer) OR AUTHKEY(Etoposide, alpha D Glucopyranosyl Isomer) OR AUTHKEY(alpha-D-Glucopyranosyl Isomer Etoposide) OR AUTHKEY(Etoposido Ferrer Farma) OR AUTHKEY(Exitop) OR AUTHKEY(Lastet) OR AUTHKEY(NSC?141540) OR AUTHKEY(NSC141540) OR AUTHKEY(Onkoposid) OR AUTHKEY(Riboposid) OR AUTHKEY(Toposar) OR AUTHKEY(Etoposide Teva) OR AUTHKEY(Vepesid) OR AUTHKEY(VP?16?213) OR AUTHKEY(VP 16213) OR AUTHKEY(VP?16) OR AUTHKEY(VP16) OR AUTHKEY(Vépéside?Sandoz) OR AUTHKEY(Celltop) OR AUTHKEY(Etopos) OR AUTHKEY(Etomedac) OR AUTHKEY(Eposin) OR AUTHKEY(Etoposide Pierre Fabre) OR AUTHKEY(fluorouracil) OR AUTHKEY(5FU) OR AUTHKEY(5?FU) OR AUTHKEY(5?Fluorouracil) OR AUTHKEY(Fluoruracil) OR AUTHKEY(Adrucil) OR AUTHKEY(Carac) OR AUTHKEY(Efudix) OR AUTHKEY(Fluoro?Uracile) OR AUTHKEY(Efudex) OR AUTHKEY(Fluoroplex) OR AUTHKEY(Flurodex) OR AUTHKEY(Fluorouracil?GRY) OR AUTHKEY(Fluorouracile Dakota) OR AUTHKEY(Fluorouracilo Ferrer Far) OR AUTHKEY(Fluracedyl) OR AUTHKEY(Haemato?FU) OR AUTHKEY(Neofluor) OR AUTHKEY(Onkofluor) OR AUTHKEY(Ribofluor) OR AUTHKEY(5?Fluorouracil?Biosyn) OR AUTHKEY(hydroxyurea) OR AUTHKEY(Hydroxycarbamid) OR AUTHKEY(Oncocarbide) OR AUTHKEY(Hydrea) OR AUTHKEY(cyclophosphamid*) OR AUTHKEY(Sendoxan) OR AUTHKEY(B?518) OR AUTHKEY(B518) OR AUTHKEY(cytophosphan*) OR AUTHKEY(Cytoxan) OR AUTHKEY(Endoxan) OR AUTHKEY(Neosar) OR AUTHKEY(NSC?26271) OR AUTHKEY(NSC26271) OR AUTHKEY(Procytox) OR AUTHKEY(Cyclophosphane) OR AUTHKEY(doxorubicin) OR AUTHKEY(Farmiblastina) OR AUTHKEY(Ribodoxo) OR AUTHKEY(Rubex) OR AUTHKEY(Adriamycin) OR AUTHKEY(Adriblastin*) OR AUTHKEY(Adriablastin*) OR

AUTHKEY(Adrimedac) OR AUTHKEY(DOXO?cell) OR AUTHKEY(Urokit Doxo?cell) OR AUTHKEY(Doxolem) OR AUTHKEY(Doxorubicin Hydrochloride) OR AUTHKEY(Doxotec) OR AUTHKEY(Myocet) OR AUTHKEY(Onkodox) OR AUTHKEY(vincristin*) OR AUTHKEY(Leurocristin*) OR AUTHKEY(cellcristin) OR AUTHKEY(Citomid) OR AUTHKEY(Oncovin*) OR AUTHKEY(Onkocristin) OR AUTHKEY(Vincasar) OR AUTHKEY(Vintec) OR AUTHKEY(Vincrisul) OR AUTHKEY(VEC regimen) OR AUTHKEY(CEV regimen) OR AUTHKEY(VEJ regimen) OR AUTHKEY(platinum?based) OR AUTHKEY(41575?94?4) OR AUTHKEY(C?tuximab) OR AUTHKEY(Chimeric Anti-EGFR Monoclonal Antibody) OR AUTHKEY(Chimeric MoAb C225) OR AUTHKEY(Chimeric Monoclonal Antibody C225) OR AUTHKEY(EGFR inhibitor) OR AUTHKEY(targeted therap*) OR AUTHKEY(immunotherap*) OR AUTHKEY(immune checkpoint inhibitor*) OR AUTHKEY(33069?62?4) OR AUTHKEY(taxane?based) OR AUTHKEY(taxane*) OR AUTHKEY(taxol) OR AUTHKEY(mitotic inhibit*) OR AUTHKEY(abraxane) OR AUTHKEY(onxol) OR AUTHKEY(PTX) OR AUTHKEY(taxotere) OR AUTHKEY(trihydrate) OR AUTHKEY(DTX) OR AUTHKEY(DXL) OR AUTHKEY(VP?16?213) OR AUTHKEY(celltop) OR AUTHKEY(eposide) OR AUTHKEY(VP-16) OR AUTHKEY(Carbamohydroxamic acid) OR AUTHKEY(N-Carbamoylhydroxylamine) OR AUTHKEY(N-Hydroxyurea) OR AUTHKEY(Biosupressin) OR AUTHKEY(Carbamohydroxamic Acid) OR AUTHKEY(Carbamoyl Oxime) OR AUTHKEY(Droxia) OR AUTHKEY(HU) OR AUTHKEY(Hidrix) OR AUTHKEY(hydroxylurea) OR AUTHKEY(Hydura) OR AUTHKEY(Hydurea) OR AUTHKEY(N-Carbamoylhydroxylamine) OR AUTHKEY(NCI C04831) OR AUTHKEY(NSC?32065) OR AUTHKEY(Oxyurea) OR AUTHKEY(SK 22591) OR AUTHKEY(SQ 1089) OR AUTHKEY(127?07?1) OR AUTHKEY(CEV) OR AUTHKEY(VEJ) OR AUTHKEY(Cyclophosphamide-Etoposide-Vincristine) OR AUTHKEY(ADR) OR AUTHKEY(2 Dox) OR AUTHKEY(hydroxydaunorubicin) OR AUTHKEY(hydroxydaunomycin) OR AUTHKEY(Cyclo) OR AUTHKEY(CPA) OR AUTHKEY(CPM) OR AUTHKEY(CTX) OR AUTHKEY(CYC) OR AUTHKEY(CYT) OR AUTHKEY(Neosar) OR AUTHKEY(Cytoxan) OR AUTHKEY(Procytox) OR AUTHKEY(Citomid) OR AUTHKEY(VCR) OR AUTHKEY(22?Oxovincaleukoblastine)))
