# Supplementary material for: Non-Cisplatin Concurrent Systemic Therapy with Radiotherapy for Locally Advanced Head and Neck Squamous Cell Carcinoma: A Network Meta-Analysis of Randomized Clinical Trials
Source: Cancers (Basel). 2026 May 14;18(10):1599. doi: 10.3390/cancers18101599 (PMC13204043; doi:10.3390/cancers18101599)
Supplement: Supplementary file 1 [file cancers-18-01599-s001.zip › cancers-4313239-supplementary/Supplementary material 4.pdf]

Supplementary material 3: Detailed characteristics of included studies with results for treatment outcome and toxicity

Authors/ study name	Journal & Year	Study population (total)	Tumor characteristics/ Histology	Compared interventions	Total dose/dose per fraction (Gy)	Dose of chemotherapy	Observation period (years)	Main reported Outcomes:	Relevant clinical parameters and/or side effects
**Al-Saleh et al. [1]**	Gulf J Oncolog, 2019	N= 40<br><br>Median age: 51 (27-72)<br><br>12,5% female	SCCHN of<br>**oropharynx** 27,5%<br>**hypopharynx** 12,5%<br>**oral cavity** 20%<br>**larynx** 35%<br>**nasal sinuses** 2,5%<br>**undetermined** 2,5%<br><br>**Stage IIA - IV**<br>Stage IIA: 2,5%<br>Stage III: 32,5%<br>Stage IV: 62,5%<br>Stage x: 2,5%	Arm A:<br>N= 22<br>Platinum based chemotherapy + IMRT<br><br>Arm B:<br>N= 18<br>Cetuximab + IMRT	**IMRT**<br><br>**Primary tumor:** 69.6 Gy / 1.2 Gy twice daily<br><br>**High-risk lymphatics:** 1.2 Gy, twice daily<br><br>**Boost:** 6 Gy/ 1.2 Gy	**Cisplatin:** 100 mg/m^2<br>3-weekly or 40 mg/ m^2 weekly<br><br>**Cetuximab:** loading dose 400 mg/ m^2; 250 mg/m^2 during radiation	Max. 5 years<br><br>Median: N/A	Primary endpoints:<br>**LC:**<br>A: 27,3% vs. B: 38,9%<br>**2-year DFS:**<br>A: 56,5% vs. B: 77,3%<br><br>Secondary endpoints:<br>**2-year OS:**<br>A: 80,7% vs. B: 57,3%<br>**acute and late AE:**<br>Hematologic: A= 100% vs. B= 100%<br>Nausea/Vomiting: A= 100% vs. B= 100%<br>Renal Toxicity: A= 27,3% vs. B= 0%<br>Skin Toxicity: A= 100% vs. B= 100%<br>Mucositis: A= 100% vs. B= 100%<br>Xerostomia: A= 100% vs. B= 100%<br>Weight loss: A= 100% vs. B= 100%	300 patients were planned for recruiting but it was stopped after negative reports of DeEscalate and RTOG 10.16
**Argiris et al. [2] NCT00703976**	Ann Oncol, 2016	N = 80 randomized/ 78 treated<br><br>*(2 were found ineligible and withdrawn prior to treatment)*<br><br>Median age: 56 (35-76)<br><br>19,2% female	SCCHN of<br>**oropharynx** 84,6%<br>**larynx** 15,4%<br><br>**Stage III / IVB**<br>Stage III: 20,5%<br>Stage IV: 79,5%	Arm A:<br>N= 37<br>Cetuximab + Pemetrexed + RT<br><br>Arm B:<br>N= 41<br>Cetuximab + Pemetrexed + Bevacizumab + RT + maintenance Bevacizumab 6 mo	70-74 Gy / 2 Gy	**Cetuximab:** loading dose 400 mg/ m^2; 250 mg/m^2 during radiation<br><br>**Pemetrexed:**<br>500 mg/m^2 on days 1, 22, 43<br><br>**Bevacizumab:**<br>15 mg/kg on days 1, 22, 43<br>+ 6 mo maintenance	Max. 5 years<br><br>Median follow-up: 32 mo among 58 censored patients	Primary endpoint:<br>**2-year PFS**<br>A= 79% *(90% CI 0.69–0.92) vs.* B= 75% *(90% CI 0.64–0.88)*<br><br>Secondary endpoints:<br>**Locoregional/distant PFS (no. patients)**<br>A= 8 (3 locoregional, 4 distant, 1 both) vs. B= 11 (3 locoregional, 4 distant, 4 both, 1 death)<br>**2-year OS**<br>A= 96% vs. B= 86%	Predominantly oropharyngeal patients, increased rate of hemorrhage with bevacizumab

								**Toxicity**<br>Mucositis 51% vs. 41%<br>Dysphagia 27% vs. 29%<br>Hemorrhage 0% vs. 7%<br>**General QoL**<br>Baseline 59 patients<br>*M= 85.06, SD= 13.41*<br>3 mo post-RT 50 patients *M= 83.72, SD= 15.36; t(42) = 1.30; p= 0.20*<br>1-year post-RT 35 patients *M= 90.53, SD= 12.65; t(27) =−2.78, p= 0.010*<br>**Head and neck specific QoL**<br>Baseline 59 patients<br>*M= 26.75, SD = 7.2*<br>3 mo post-RT 50 patients *M= 20.27, SD = 6.49; t(43) = 6.54, p < 0.0001*<br>1-year post-RT 35 patients *M= 23.28, SD = 5.93; t(28) = 2.13, p = 0.042*	
**Bonner et al. [3] NCT00004227**	N Engl J Med, 2006	N = 424<br><br>Median age RT: 58 (35-83)<br><br>Median age CRT: 56 (34-81)<br><br>19,8% female	SCCHN of **oropharynx** 59,7%<br>**Hypopharynx** 14,8%<br>**Larynx** 25,5%<br><br>**Stage III / IV**<br>Stage III: 25,2%<br>Stage IV: 74,8%	<u>Arm A:</u><br>N= 211<br>Cetuximab + RT<br><br><u>Arm B:</u><br>N= 213<br>RT	**Once daily:** 70 Gy / 2 Gy<br><br>**Twice daily:** 72.0-76.8 Gy / 1,2Gy<br><br>**Concomitant boost:** 54 Gy / 1,8 Gy and<br><br>Boost ad 72 Gy:<br><br>18Gy / 1,5 Gy delivered as a second daily fraction during the last 12d of treatment	**Cetuximab:** loading dose 400 mg/ m^2; 250 mg/m^2 during radiation	Max. 5 years<br><br>Median follow-up: 54 mo	<u>Primary endpoint:</u><br>**Median LC**<br>A= 24,4 mo vs. B= 14,9 mo<br>**2-year LC**<br>A= 50% vs. B= 41%; *HR: 0.68 (95% CI: 0.52–0.89) p= 0.005*<br><br><u>Secondary endpoints:</u><br>**Median OS**<br>A= 49,0 mo vs. B= 29,3 mo; *HR: 0.74 (95% CI: 0.57–0.97) p= 0.03*<br>**3-year OS**<br>A= 55% vs. B= 45%<br>**Median PFS**	

								A= 17,1 mo vs. B= 12,4 mo; *HR: 0.70 (95% CI: 0.54–0.90) p= 0.006*<br>**2-year PFS**<br>A= 46% vs. B= 37%<br>**RR**<br>A= 74% vs. B= 64%; *OR= 0.57 (95% CI: 0,36-0,90) p= 0,02*<br>**Safety**<br>Acneiform rash: 17% vs. 1% p< 0,001<br>Infusion reaction: 3% vs. 0% p= 0,01<br>Anemia: 1% vs. 6% p= 0,006	
**Bourhis et al. [4] GORTEC 99-02 NCT00828386**	Lancet, 2012	N = 840<br><br>Mean age Arm A: 56,1 (39-74)<br><br>Mean age Arm B: 56,9 (37-75)<br><br>Mean age Arm C: 56,5 (34-74)<br><br>13% female	SCCHN of **oropharynx** 66%<br>**Hypopharynx** 16,8%<br>**oral cavity** 10,2%<br>**larynx** 6,3%<br>**no primary** 0,7%<br><br>**Stage III / IV**<br>Stage T0: 0,8%<br>Stage T1: 0,1%<br>Stage T2: 8,6%<br>Stage T3: 34,9%<br>Stage T4: 55,6%	<u>Arm A:</u><br>N= 279<br>Carboplatin + 5-FU + RT<br><br><u>Arm B:</u><br>N= 280<br>Accelerated CRT<br><br><u>Arm C:</u><br>N= 281<br>Very accelerated RT	<u>Conventional RT</u>: 70 Gy / 2 Gy<br>5 fractions per week<br><br><u>Acclerated RT:</u><br>70 Gy<br>2 Gy /fraction until 40 Gy, then 1,5 Gy /fraction twice daily<br>RT 5 days per week<br><br><u>Very accelerated RT:</u><br>64,8 Gy / 1,8 Gy twice daily<br>RT 5 days per week	<u>Conventional CRT:</u><br>Carboplatin: 70mg/m^2 on days 1-4, 22-25, 43-46<br><br>5-FU: 600mg/m^2 on days 1-4, 22-25, 43-46<br><br><u>Accelerated CRT:</u><br>Carboplatin: 70mg/m^2 on days 1-5, 29-33<br><br>5-FU: 600mg/m^2 on days 1-5, 29-33	Median follow-up 5,2 years	<u>Primary endpoint:</u><br>**3-year PFS**<br>A= 37,6% vs. B= 34,1% vs. C= 32,2%<br>*HR (B vs. A) 1,02; p=0,88*<br>*HR (B vs. C) 0,83; p=0,06*<br>*HR (A vs. C) 0,82; p=0,041*<br><br><u>Secondary endpoints:</u><br>**3-year locoregional progression**<br>A= 41,7% vs. B= 45,4% vs. C= 49,9%<br>*HR (B vs. A) 0,97; p=0,81*<br>*HR (B vs. C) 0,76; p=0,033*<br>*HR (A vs. C) 0,77; p=0,045*<br>**3-year distant metastases**<br>A= 25,3% vs. B= 34% vs. C= 28,1%<br>*HR (B vs. A) 1,26; p=0,18*<br>*HR (B vs. C) 1,16; p=0,38*<br>*HR (A vs. C) 0,96; p=0,82*<br>**3-year OS**<br>A= 42,6% vs. B= 39,4% vs. C= 36,5%<br>*HR (B vs. A) 1,05; p=0,6*	No benefit from combining accelerated RT with concomitant CT compared to conventional CRT<br><br>Different cycles of CRT between CRT and accelerated CRT<br><br>Very accelerated RT had worst outcome of all treatment arms<br><br>High rate of early deaths - patients in poor condition

								HR (B vs. C) 0,87; p=0,169<br>*HR (A vs. C) 0,81; p=0,04*<br>**toxicity**<br>Feeding tube 60% vs. 64% vs. 70%; *p= 0,045*<br>Mucositis<br>Grade 0-2 31% vs. 24% vs. 16%<br>Grade 3-4 69% vs. 76% vs. 84%; *p= 0,016*	
**Budach et al. [5]; [6]<br>ARO 95-06**	J Clin Oncol, 2005<br><br>Int J Radiat Oncol Biol Phys, 2015	N = 384<br><br>Median age: 55 (33-71)<br><br>16,1% female	SCCHN of<br>**Oropharynx** 59,4%<br>**Hypopharynx** 32,3%<br>**oral cavity** 8,3%<br><br>**Stage III / IV**<br>Stage III: 6%<br>Stage IV: 94%	Arm A:<br>N= 190<br>Mitomycin-C + 5-FU + RT<br><br>Arm B:<br>N= 194<br>HART	**Arm A:**<br>30 Gy/ 2 Gy followed by 1.4 Gy twice daily to 70.6 Gy<br><br>**Arm B:**<br>16 Gy/ 2 Gy followed by 1.4 Gy twice daily to 77.6 Gy (HART)	5-FU: 600 mg/m^2 days 1 to 5<br><br>Mitomycin-C: 10 mg/m^2 on days 5 and 36	Max. 16 years<br><br>Median follow up: 8.7 years	Primary endpoint:<br>**5-year LC**<br>A= 49% vs. B= 35%<br>**10-year LC**<br>A= 38% vs. B= 26%; *p= 0,002*<br>**Median LC (years)**<br>A= 4 (95% CI: 0.97-7.0) vs. B= 1,2 (95% CI: 0.86-1.6)<br><br>Secondary endpoints:<br>**5-year OS**<br>A= 27% vs. B= 20%<br>**10-year OS**<br>A= 10% vs. B= 9%; *p=0,049*<br>**Median OS (years)**<br>A= 1.9 (95% CI: 1.4-2.4) vs. B= 1.3 (95% CI: 1.0-1.6)<br>**5-year PFS**<br>A= 30% vs. B= 25%<br>**10-year PFS**<br>A= 25% vs. B= 18%; *p= 0,033*<br>**Median PFS (years)**<br>A= 1.42 (95% CI: 0.99-1.8) vs. B= 0.92 (95% CI: 0.72-1.1)<br>**5-year cancer specific survival**<br>A= 41% vs. B= 33%<br>**10-year cancer specific survival**	Limited data on long-term late morbidity

								A= 39% vs. B= 30%; *p= 0,042*<br>**Median cancer specific survival (years)**<br>A= 2.7 (95% CI: 1.7-3.7) vs. B= 1.7 (95% CI: 1.4-2.1)<br>**5-year freedom from distant metastasis**<br>A= 54% vs. B= 55%<br>**10-year freedom from distant metastasis**<br>A= 52% vs. B= 48%; *p= 0,64*	
**Chitapanarux et al. [7]**	J Radiat Res, 2013	N = 85<br><br>Median age Arm A: 58,5 (28-70)<br><br>Median age Arm B: 63,5 (40-77)<br><br>24,7% female	SCCHN of **oropharynx** 16,5% **Hypopharynx** 4,7% **oral cavity** 30,6% **larynx** 48,2%<br><br>**Stage III / IV**<br>Stage III: 41,1%<br>Stage IV: 58,9%	<u>Arm A:</u><br>N= 37<br>RT<br><br><u>Arm B:</u><br>N= 48<br>Carboplatin + 5-FU + RT	**Arm A:**<br>70 Gy<br>2 Gy /fraction until 40 Gy, then 1,8 Gy first RT/day and 1,2 Gy second RT/day<br>RT 5 days per week<br><br>**Arm B:**<br>66 Gy / 2 Gy<br>5 fractions per week	Carboplatin 70 mg/m^2 on days 1, 22, 43<br><br>5-FU 600 mg/m^2 on days 1, 22, 43	Median follow-up 43 mo	<u>Primary endpoint:</u><br>**5-year LC**<br>55% vs. 69,6%; *p= 0,18*<br><br><u>Secondary endpoints:</u><br>**5-year OS**<br>63,5% vs. 76,1%; *p= 0,05*<br>**Compliance**<br>Median treatment time: 49 days vs. 55,5 days<br>Radiation interruption >3d: 40,5% vs. 60,4%<br>**Acute toxicity** (Grade 3/4)<br>Mucositis 67,6% vs. 41,7%; *p= 0,01*<br>Skin 16,2% vs. 25%; *p=0,33*<br>Anemia 0% vs. 2,1%; *p=0,25*<br>Leukopenia 0% vs. 18,8%; *p= 0,00*<br>Thrombocytopenia 0% vs. 6,3%; *p= 0,3*<br>**Late toxicity** (Grade 2-4)<br>Skin 76% vs. 76,1%; *p=0,97*<br>Subcutaneous 60% vs. 26,1%; *p= 0,01*<br>Mucous membrane 84% vs. 73,9%; *p= 0,35*	Small sample size<br><br>Different median age in treatment arms<br><br>Lower compliance in Arm B than in Arm A

								Salivary glands 60% vs. 54,3%; *p= 0,89*	
**Essa et al. [8]**	J Egypt Natl Canc Inst, 2010	N = 52 enrolled/ 41 eligible<br><br>Mean age Group 1: 55,1<br><br>Mean age Group 2: 55,7<br><br>19,5% female	SCCHN of<br>**Oropharynx** 9,8%<br>**Hypopharynx** 36,6%<br>**Supraglottic** 34,1%<br>**Glottic** 19,5%<br><br>**Stage III / IV**<br>Stage III: 29,3%<br>Stage IV: 70,7%	<u>Arm A:</u><br>N= 21<br>Paclitaxel + RT<br><br><u>Arm B:</u><br>N= 20<br>Cisplatin + RT	66-70 Gy / 2 Gy	Paclitaxel: 30mg/m^2 weekly<br><br>Cisplatin: 30mg/m^2 weekly	Max. 2.5 years<br><br>Median follow up: 15/16.5 mo	<u>Primary endpoint:</u><br>**RR**<br>Complete response 57,1% vs. 50%<br>Partial response 28,6% vs. 25%<br>Overall response 85,7% vs. 75%<br>No response 14,3% vs. 25%<br>*p= 0,3*<br><br><u>Secondary endpoints:</u><br>**Median PFS**<br>A= 26 mo vs. B= 22 mo<br>**1-year PFS**<br>A= 80,7% vs. B= 64%<br>**2-year PFS**<br>A= 52,2% vs. B= 41,1%<br>*p= 0,5*<br>**Median OS**<br>A= 27 mo vs. B= 25 mo<br>**1-year OS**<br>A= 80,7% vs. B= 64,6%<br>**2-year OS**<br>A= 58,4% vs. B= 46%; *p= 0,41*<br>**Toxicity**<br>Skin G1&2 81% vs. 90%<br>Skin G3&4 19% vs. 10%<br>*p= 0,6*<br>Mucositis G1&2 52,4% vs. 75%<br>Mucositis G3&4 47,6% vs. 25%<br>*p= 0,2*<br>Dysphagia G1&2 71,4% vs. 80%<br>Dysphagia G3&4 28,6% vs. 20%	Lots of stage IV disease patients

								p= 0,6	
**Ezzat et al. [9]**	J Egypt Natl Canc Inst, 2005	N = 60<br><br>Mean age Arm A: 53<br><br>Mean age Arm B: 54<br><br>Mean age Arm C: 49<br><br>75% female	SCCHN of **oropharynx** 23% **Hypopharynx** 23% **oral cavity** 25% **larynx** 29%<br><br>**Stage III / IV**<br>Stage III: 40%<br>Stage IV: 60%	Arm A:<br>N= 20<br>RT<br><br>Arm B:<br>N= 20<br>Accelerated RT<br><br>Arm C:<br>N= 20<br>Mitomycin C + accelerated RT	Arm A:<br>68 Gy / 2 Gy<br>5 fractions per week<br><br>Arm B + C:<br>68 Gy / 2 Gy<br>6 fractions per week	Mitomycin C: 15mg/m^2 at the end of 1st week	Median follow-up 10,5 mo (6-42 mo)	Endpoints:<br>**RR**<br>20% vs. 45% vs. 35%<br>**Median RR-time:** 7 mo<br>**Distant metastases**<br>5% vs. 15% vs. 10%; *p=0,2*<br>**2-year OS**<br>23% vs. 20% vs. 28%; *p< 0,19*<br>**2-year LC**<br>10% vs. 25% vs. 30%; *p< 0,27*<br>**AE**<br>Mucositis 55% vs. 70% vs. 90%; *p< 0,04*<br>Hematological toxicity 10% vs. 5% vs. 40%; *p=0,04*	Small sample size<br><br>Addition of MMC improved OS<br><br>Accelerated RT and/or MMC did not increase toxicity
**Fallai et al. [10], [11]**	Tumori, 2006	N = 192<br><br>Median age: 56,1<br><br>11,5% female	SCCHN of **oropharynx**<br><br>**Stage TNM**<br>T1: 3,1%<br>T2: 12,5%<br>T3: 52,6%<br>T4: 26,6%<br>Tx: 5,2%	Arm A:<br>N= 63<br>RT<br><br>Arm B:<br>N= 65<br>HART<br><br>Arm C:<br>N= 64<br>Carboplatin + 5-FU + RT	**Arms A&C:** 66-70 Gy/ 2 Gy<br><br>**Arm B:** 64-67.2 Gy 2 fractions daily with 1.6 Gy, after reaching 38.4 Gy 2-week split than resume regimen up to complete dose with previous bi-daily fraction of 1.6 Gy	Carboplatin: 75 mg/m^2, days 1-4<br><br>5-FU: 1,000 mg/m^2, days 1-4	Median follow-up 8,35 years (4.8-10.2 years)	Endpoints:<br>**Median OS**<br>A= 556 d vs. B= 445 d vs. C= 571 d<br>**5-year OS**<br>A= 21% vs. B= 20% vs. C= 40%; *p= 0,4*<br>**5-year LC**<br>A= 22% vs. B= 26% vs. C= 43%; *p= 0,2*<br>**AE**<br>Acute<br>Weight loss 60,3% vs. 64,6% vs. 62,5%<br>Skin G3 3,7% vs. 3,7% vs. 16%<br>Mucosa G3 12,9% vs. 40,3% vs. 44%<br>Ear G3 0% vs. 3,8% vs. 0%	Limited number of patients per arm

								Salivary glands G3 05 vs. 1,9% vs. 0%<br>Pharynx G3 11,1% vs. 23% vs. 24%<br>Larynx G3 0% vs. 0% vs. 2%<br><u>Late</u><br>Skin 0% vs. 0% vs. 2.5%<br>Subcutaneus tissue 0% vs. 0% vs. 5,1%<br>Mucosa 0% vs. 2,7% vs. 5,1%<br>Salivary glands 5,7% vs. 10,8% vs. 7,7%<br>**5-year relapse-free survival**<br>A= 15% vs. B= 17% vs. C= 36%; *p= 0,17*<br>**5-year event-free survival**<br>A= 13% vs. B= 16,5% vs. C= 31%; *p= 0,17*	
**Gebre-Medhin et al. [12]**<br>**ARTSCAN III**<br>**NCT01969877**	J Clin Oncol, 2021	N = 298<br><br>Median age: 61 (33-77)<br><br>20% female	SCCHN of<br>**Oropharynx** 83,2%<br>**Oral cavity** 5%<br>**Hypopharynx** 5,4%<br>**Larynx** 6,4%<br><br>**Stage III / IV**<br>Stage III: 10%<br>Stage IV: 90%	<u>Arm A:</u><br>N= 149<br>Cisplatin + RT<br><br><u>Arm B:</u><br>N= 149<br>Cetuximab + RT	**Primary tumor:** 68 Gy/ 2 Gy<br><br>**Lymph node MTS:** 54.4 Gy / 1.6 Gy	Cisplatin: 40 mg/m^2<br><br>Cetuximab: loading dose of 400 mg/m^2, seven weekly doses of 250 mg/m^2	Max. 5 years<br><br>Median follow-up Group A: 38 mo<br><br>Median follow-up group B: 39 mo	<u>Primary endpoint:</u><br>**3-year OS**<br>A= 88% vs. B= 78%; *HR: 1.63 (95% CI: 0.93 - 2.86); p= 0,075*<br><br><u>Secondary endpoints:</u><br>**3-year LC**<br>A= 9% vs. B= 23%; *HR: 2.49 (95% CI: 1.33 - 4.66) p= 0,0036*<br>**AE**<br><u>Acute AE</u><br>Dysphagia 32% vs. 21%; *p= 0,033*<br>Mucositis 60% vs. 72%; *p= 0,035*<br>Skin 8% vs. 22%; *p= 0,001*<br>Acneiform rash 0% vs. 21%; *p<0,001*<br>Nausea 28% vs. 11%; *p= 0,001*	Patient inclusion ended prematurely, OS did not reach significance

								Vomiting 5% vs. 0%; *p= 0,015*<br>Kidney injury 13% vs. 1%; *p<0,001*<br>Neutropenia 11% vs. 1%; *p<0,001*<br>Tinnitus 10% vs. 1%; *p= 0,002*<br>Late AE<br>Pain 10% vs. 19%; *p= 0,046*<br>Oral mucosa status G3/4 0% vs. 4%; *p= 0,023*<br>Taste alteration 15% vs. 5%; *p= 0,013*<br>Hearing impairment 8% vs. 2%; *p= 0,022*<br>**Local control**<br>HR 0,77 (95% CI: 0,35 - 1,67); *p= 0,5*<br>**3-year pattern of failure**<br>distant failure 6% vs. 9%; *p= 0,52*<br>Event free survival 85% vs. 67%; *p= 0,0054*	
**Geoffrois et al. [13] GORTEC 2007-02**	J Clin Oncol, 2018	N = 370<br><br>Median age Group 1: 56<br><br>Median age Group 2: 56.5<br><br>15% female Group 1<br><br>13% female Group 2	SCCHN of **Oropharynx** 63,2% **Hypopharynx** 19,2% **oral cavity** 11,6% **larynx** 5,6%<br><br>**Stage III / IV**<br>Stage T2: 16,4%<br>Stage T3: 34,2%<br>Stage T4: 49,4%	Arm A:<br>N= 186<br>Neoadjuvant TPF + Cetuximab + RT<br><br>Arm B:<br>N= 184<br>Carboplatin + 5-FU + RT	70 Gy/ 2 Gy<br><br>5 days per week	TPF: docetaxel 75 mg/m^2 day 1 + cisplatin 75 mg/m^2 day 1 + 5-FU 750 mg/m^2 days 1 to 5<br><br>Cetuximab: loading dose of 400 mg/m^2, 250 mg/m^2 during radiation<br><br>Carboplatin: 70mg/m^2 daily<br><br>5-FU: 600 mg/m^2 days 1 to 4	Median follow-up Group A: 2,8 years<br><br>Median follow-up Group B: 2,6 years	Primary endpoint:<br>**PFS** HR of 0.93; *p= 0.58*<br><br>Secondary endpoints:<br>**OS** HR of 1.12; *p= 0.39*<br>**Locoregional failure** HR of 0.98; *p= 0.9*<br>**distant failure** HR of 0.54; *p= 0.05*<br>**acute and late toxicity**<br>Fever 9% vs. 0.6%; *p= 0.001*<br>Neutropenia 26% vs. 6%; *p= 0.001*<br>Febrile neutropenia 17% vs. 0%; *p= 0.001*<br>Mucositis 48% vs. 50%; *p= 0,7*<br>Skin 53% vs. 29%; *p= 0.001*	Primary endpoint not met, similar results for both groups (except distant metastases)<br>High TPF-related number of deaths

								Deaths 6,6% vs. 0,6%; *p= 0,0016*	
**Gillison et al. [14]**<br>**RTOG 1016**	Lancet, 2019	N = 987 enrolled/ 849 randomized<br><br>Median age: 58 (33-83)<br><br>10% female	SCCHN of **oropharynx**<br>Tonsilla fossa/tonsil 50%<br>Base of tongue 44%<br>Oropharynx 4%<br>Pharyngeal oropharynx 2%<br>Soft palate <1%<br>Vallecula <1%<br><br>**Stage III / IV**<br>Stage III: 7%<br>Stage IV: 93%	<u>Arm A:</u><br>N= 424<br>Cisplatin + IMRT<br><br><u>Arm B:</u><br>N= 425<br>Cetuximab + IMRT	Accelerated IMRT<br><br>70 Gy / 2 Gy<br><br>6 fractions per week; once a week 2 fractions a day min. 6h apart	Cisplatin: 100 mg/m^2 days 1 and 22<br><br>Cetuximab: loading dose of 400 mg/m^2, 250 mg/m^2 during radiation	Median follow-up 4,5 years	<u>Primary endpoint:</u><br>**5-year OS**<br>A= 84,6% vs. B= 77,9%; *HR 1,4; p= 0,5056*<br><br><u>Secondary endpoints:</u><br>**PFS** HR 1,72; *p=0,0002*<br>**5-year PFS**<br>A= 78,4% vs. B= 67,3%<br>**LC** HR 2,05; *p=0,0005*<br>**5-year locoregional failure**<br>A= 9,9% vs. B= 17,3%<br>**distant metastasis** HR 1,49; *p=0,09*<br>A= 8,6% vs. B= 11,7%<br>**second primary tumors** HR 0,99 (95% CI: 0,61-1,58); *p= 0,95*<br>**AE**<br>Anaemia 2,8% vs. 0%; *p=0,0009*<br>Hearing (acute) 3% vs. 0,3%; *p= 0,0032*<br>Nausea 19,1% vs. 8,1%; *p<0,0001*<br>Vomiting 12,1% vs. 4,1%; *p<0,0001*<br>Neutrophil count decreased 15,3% vs. 0,5%; *p<0,0001*<br>White blood cells decreased 12,1% vs. 0%; *p<0,0001*<br>Anorexia 22,4% vs. 15,5%; *p= 0,0144*<br>Dehydratation 15,3% vs. 6,1%; *p<0,0001*<br>Hyponatremia 5,3% vs. 1%; *p=0,0008*	Greater survival benefit for cetuximab for HPV pos. patients<br><br>High, intermediate and low risk patients included

								Acute kidney injury 3,3% vs. 0,3%; *p=0,0017*<br>Acneiform rash 0,3% vs. 9,4%; *p<0,0001*<br>Hearing (late) 6,3% vs. 2,1%; *p=0,006*<br>**Feeding tube placement**<br>End of treatment: 61,5% vs. 57,3%<br>1-year: 9,2% vs. 8,4%<br>*p= 0,79*<br>**Dental health**<br>Before treatment: 71% vs. 75% / 20,9 native teeth vs. 21,4 native teeth<br>1-year: 87% vs. 84% / 1,05 teeth lost vs. 1,64 teeth lost<br>**QoL (Swallowing)**<br>End of treatment: 48% vs. 47,4%; *p= 0,86*<br>1-year: 2,5% vs. 7,6%; *p= 0,0382*	
**Giralt et al. [15]<br>CONCERT-2<br>NCT00547157**	Lancet Oncol, 2015	N = 152 enrolled / 151 treated<br><br>Median age: N/A<br><br>83,4% > 65 years<br>16,6% < 65 years<br><br>19% female	SCCHN of **oropharynx** 48,3%<br>**Hypopharynx** 15,2%<br>**oral cavity** 11,9%<br>**larynx** 24,6%<br><br>**Stage III / IVB**<br>T1: 6,6%<br>T2: 17,2%<br>T3: 40,4%<br>T4: 35,8%	<u>Arm A:</u><br>N= 90<br>Panitumumab + RT<br><br><u>Arm B:</u><br>N= 61<br>Cisplatin + RT	70–72 Gy / 2-2.4 Gy<br><br>5 days per week, one or two fractions per day<br><br>IMRT or 3D conformal RT	Cisplatin: 100 mg/m^2 days 1 and 22<br><br>Panitumumab: 9.0 mg/kg days 1, 22, 43	Max. 4 years<br><br>Median follow-up (Arm A) 107,5 weeks<br><br>Median follow-up (Arm B) 123 weeks	<u>Primary endpoint:</u><br>**LC at 2 years**<br>A= 51% vs. B= 61%<br>**Locoregional failure**<br>A= 53% vs. B= 38%<br>**Never had LC**<br>A= 30% vs. B= 23%<br>**LC** HR 1,61 (95% CI: 0,98–2,66); *p=0,06*<br><br><u>Secondary endpoints:</u><br>**2-year PFS**<br>A= 41% vs. B= 62%<br>**PFS** HR 1,73 (95% CI: 1,07–2,81); *p= 0,03*<br>**2-year OS**<br>A= 63% vs. B= 71%<br>**OS** HR 1,59 (95% CI: 0,91–2,79); *p= 0,1*<br>**Patients treated according protocol**	2-year LC panitumumab with 51% inferior to 61% cisplatin<br><br>More toxic related deaths in panitumumab group<br><br>Low HPV pos. patient number (16%)

								Median relative dose intensity: 100% vs. 99%<br>Treatment interruptions (>10d): 7% vs. 0%<br>Median panitumumab delay: 3d (arm A)<br>Median chemotherapy delay: 4d (arm B)<br>Median duration of RT interruption: 2d vs. 2d<br>Nodal surgery: 14% vs. 23%<br>**AE**<br>Skin 2% vs. 0%<br>Neutropenia 0% vs. 16%<br>Febrile neutropenia 0% vs. 8%<br>Mucosal inflammation 42% vs. 40%<br>Dysphagia 40% vs. 32%<br>AE leading to discontinuation of treatment 11% vs. 3%<br>Deaths 6% vs. 3%	
**Halim et al. [16]**	Med Oncol, 2012	N = 216<br><br>Median age Group A: 52 (20-70)<br><br>Median age Group B: 49 (23-65)<br><br>39% female Group A<br><br>43% female Group B	SCCHN of **oropharynx** 29,6% **Hypopharynx** 32,9% **oral cavity** 8,3% **larynx** 8,3% **paranasal sinuses** 20,9%<br><br>**Stage III / IV**<br>Stage T3: 48,6%<br>Stage T4: 51,4%	<u>Arm A:</u><br>N= 110<br>Gemcitabine + RT<br><br><u>Arm B:</u><br>N= 106<br>Paclitaxel + RT	65 Gy / 2 Gy<br><br>5 days per week	Gemcitabine: 100 mg/m^2 weekly<br><br>Paclitaxel: 20 mg/m^2 weekly	Max. 5 years<br><br>Median follow-up 22 mo	<u>Endpoints:</u><br>**Toxicity**<br>Neutropenia (Grade 3) 1,8% vs. 2,8%<br>Anemia (Grade 3) 1,8% vs. 1,9%<br>Mucositis (Grade 3) 36% vs. 24%; *p= 0,04*<br>Dermatitis (Grade 3) 24% vs. 13%; *p= 0,049*<br>Enteral/Parenteral Feeding 29% vs. 17%; *p= 0,01*<br>**RR**<br>Partial/complete response 78% vs. 89%; *p= 0,038*<br>**2-year PFS**<br>A= 54% vs. B= 64%<br>**3-year PFS**<br>A= 39% vs. B= 48%<br>*p<0,05*	Paclitaxel with better results than Gemcitabine in efficacy and less toxicity

								**2-year OS**<br>A= 56% vs. B= 67%<br>**3-year OS**<br>A= 45% vs. B= 49%<br>*p<0,05*	
**Magrini et al. [17]**	J Clin Oncol, 2016	N = 70<br><br>Median age Group A: 67,5 (36-77)<br><br>Median age Group B: 61 (44-80)<br><br>28,6% female	SCCHN of<br>**oropharynx** 47,1%<br>**Hypopharynx** 20%<br>**oral cavity** 14,3%<br>**supraglottic larynx** 18,6%<br><br>**Stage III / IVB**<br>Stage III: 20%<br>Stage IVA: 65,7%<br>Stage IVB: 14,3%	Arm A:<br>N= 35<br>Cisplatin + RT<br><br>Arm B:<br>N= 35<br>Cetuximab + RT	3D conformal RT, IMRT, IMRT with simultaneous boost, helical IMRT<br><br>70 Gy / 2 Gy	Cisplatin: 40 mg/m^2<br><br>Cetuximab: loading dose of 400 mg/m^2, 250 mg/m^2 during radiation	Median follow-up Group A 41 mo<br><br>Median follow-up Group B 17 mo	Primary endpoint:<br>**Compliance**<br>Treatment break longer 10 days 0% vs. 11,4%; *p= 0,05*<br>**Toxicity**<br>Acute AE<br>Skin 21% vs. 44%; *p= 0,039*<br>Hemoglobin 12% vs. 3%*; p= 0,001*<br>Platelets 12% vs. 3%; *p=0,003*<br>Kidney injury 12% vs. 0%*; p=0,033*<br>GI toxicity 18% vs. 6%; *p= 0,036*<br>Late AE<br>Xerostomia 14,8% vs. 4,2%<br>Fibrosis 18,5% vs. 20,8%<br>Mucosal atrophy 18,5% vs. 29,2%<br>Weight loss (>10%) 18,2% vs. 18,2%<br>Weight loss any grade 72,7% vs. 68,2%<br><br>Secondary endpoints:<br>**2-year LC**<br>78% vs. 53%<br>**5-year LC**<br>67% vs. 48%<br>**5-year metastases free survival**<br>83% vs. 97%<br>**2-year CSS**<br>82% vs. 65%<br>**5-year CSS**<br>70% vs. 59%	Recruitment stopped early because of slow accrual

								**2-year OS**<br>97% vs. 75%<br>**5-year OS**<br>61% vs. 52%	
**Mehanna et al. [18]**<br>**De-ESCALaTE HPV**<br>**ISRCTN33522080**	Lancet, 2019	N = 348 registered/ 334 randomized<br><br>Median age: 57 (52-63)<br><br>20% female	SCCHN of **oropharynx**<br><br>**Stage III / IV**<br>Stage T1/T2: 65%<br>Stage T3/T4: 35%<br>Stage T4 only: 17%	Arm A:<br>N= 166<br>Cisplatin + RT<br><br>Arm B:<br>N= 168<br>Cetuximab + RT	70 Gy / 2 Gy	Cisplatin: 100 mg/m^2, days 1, 22, 43<br><br>Cetuximab: loading dose of 400 mg/m^2, 250 mg/m^2 during radiation	Median follow-up 25,9 mo	Primary endpoint:<br>**Acute and late toxicity**<br>number of events 4,8 (95% CI: 4,2–5,4) vs. 4,8 (95% CI: 4,2–5,4); *p= 0,98*<br>Overall toxicity 29,2 (95% CI: 27,3–31,0) vs. 30,1 (95% CI: 28,3–31,9); *p= 0,49*<br>Acute toxicity G3 or more 4,43 (95% CI: 3,88–4,97) vs. 4,35 (95% CI: 3,84–4,86); *p= 0,84*<br>Late toxicity G3 or more 0,41 (95% CI: 0,29–0,54) vs. 0,48 (95% CI: 0,30–0,67); *p= 0,49*<br><br>Secondary endpoints:<br>**2-year OS**<br>A= 97,5% vs. B= 89,4%; *HR 5,0 (95% CI: 1,7–14,7); p= 0,0012*<br>**2-year Recurrence rate**<br>A= 6% vs. B= 16,1%; *HR 3,4 (1,6–7,2); p=0,0007*<br>**Locoregional recurrence**<br>3% vs. 9%; *p= 0,0092*<br>**distant metastases** 3% vs. 9%; *p=0,0092*<br>**QoL**<br>mean difference at 24 mo of 1,51 points in favour of cisplatin; *p=0,9976*<br>**Swallowing**<br>mean difference at 24 mo of 6,90 points in	No treatment benefit in terms of reduced toxicity for cetuximab, while having worse tumor control

								favour of cisplatin;<br>*p=0,1279*	
**Mell et al. [19]**	Lancet Oncol, 2024	N = 190 enrolled/ 186 randomized<br><br>Median age: 72 (48-90)<br><br>16% female	SCCHN of **oropharynx 57.5%**<br>**Hypopharynx 11.3%**<br>**oral cavity 5.4%**<br>**larynx 25.8%**<br><br>**Stage**<br>Stage T0: 3.7%<br>Stage T1: 9.7%<br>Stage T2: 29%<br>Stage T3: 33.3%<br>Stage T4: 14%<br>Stage T4a: 9.1%<br>Stage T4b: 1.2%	<u>Arm A:</u><br>N= 123<br>Durvalumab<br><br><u>Arm B:</u><br>N= 63<br>Cetuximab	70 Gy / 2 Gy<br>5 fractions per week	Durvalumab 1500mg 2 weeks before RT, every 4 weeks starting week 2<br><br>Cetuximab 400mg/m^2 1 week before RT, 250mg/m^2 weekly	Median follow-up 2.3 years	<u>Primary endpoint:</u><br>**PFS** HR = 1.33 (95% CI: 0.84-2.12)<br><br><u>Secondary endpoints:</u><br>**OS** HR = 1.3 (95% CI: 0.74-2.28)<br>**Locoregional-failure** HR = 1.71 (95% CI: 0.89-2.38)<br>**Distant metastasis** HR = 0.76 (95% CI: 0.32-1.77)<br>**Competing mortality** HR 1.41 (95% CI: 0.62-3.18)<br><br>***<u>AEs</u>***<br>**Dysphagia** 22% vs. 30%<br>**Renal** 2% vs. 0%<br>**Dermatitis** 5% vs. 13%<br>**Weight loss** 12% vs. 8%<br>**Mucositis** 11% vs. 18%	Because of low efficacy and high toxicity study closed early and did not proceed to phase 3 as intended
**Mercke et al. [20]**<br>**ACCROBAT study**	Cancers, 2023	N = 152<br><br>Median age: 59,8<br><br>24,6% female	SCCHN of **oropharynx tonsil** 63,3%<br>**oropharynx base of tongue** 36,7%<br><br>**Stage III / IV**<br>Stage T1: 22,7%<br>Stage T2: 45,3%<br>Stage T3: 18%<br>Stage T4: 14%	<u>Arm A:</u><br>N= 77<br>Induction TPF + Cetuximab +RT<br><br><u>Arm B:</u><br>N= 75<br>Cetuximab + RT	68 Gy / 2 Gy<br>5-6 fractions per week, 5 days per week	<u>Induction TPF</u><br>2 cycles Docetaxel: 75mg/m^2<br>2 cycles Cisplatin: 75mg/m^2<br>Cisplatin and docetaxel with 5-FU: 1000mg/m^2<br>21 days apart<br><br><u>CRT</u><br>Cetuximab: loading dose of 400 mg/m^2, 250 mg/m^2 during radiation	Median follow-up: 39,3 mo	<u>Primary endpoint:</u><br>**2-year PFS**<br>A= 84.2% (95% CI: 76.4–92.8)<br>B= 78.4% (95% CI: 69.5–88.3)<br>*HR 1.39, 95% CI 0.69–2.79; p=0.40*<br><br><u>Secondary endpoints:</u><br>**2-year OS**<br>A= 93,4% vs. B= 90,5%<br>**2-year LC**<br>A= 96% vs. B= 97,3%<br>**RR**<br><u>T site</u><br>Complete response 38%<br>Partial response 44%<br>Stable disease 15%	

								Progressive disease 3%<br>N site<br>Complete response 40%<br>Partial response 36%<br>Stable disease 20%<br>Progressive disease 4%<br>**Pattern of failure**<br>Distant failure HR 2.40 (95% CI: 0.74–7.79); *p=0.1*<br>Locoregional failure HR 2.40 (95% CI: 0.74–7.79); *p=0.5*<br>**Toxicity**<br>Mucositis 64,9% vs. 61,3%<br>Acneiform rash 22,1% vs. 20%<br>Osteoradionecrosis 0% vs. 1,3%	
**Mesia et al. [21]**	Ann Oncol, 2012	N = 91<br><br>Median age group A: 60 (42-75)<br><br>Median age group B: 61 (45-80)<br><br>14,3 % female	SCCHN of **oropharynx:**<br>Tonsil 49,5%<br>base of tongue 33%<br>lateral/posterior wall 11%<br>soft palate 6,5%<br><br>**Stage III / IVB**<br>Stage III: 27,5%<br>Stage IV: 56%<br>Stage IVB: 16,5%	Arm A:<br>N= 45<br>Cetuximab + RT<br><br>Arm B:<br>N= 46<br>Cetuximab + RT + adjuvant Cetuximab	Control group: 70 Gy/ 2 Gy<br><br>Adjuvant Group:<br>69,9 Gy / 1,8 Gy<br><br>Boost: 1,5 Gy/day (last 13 treatment days)<br><br>5 fractions per week	Cetuximab: loading dose of 400 mg/m^2, 250 mg/m^2 during radiation<br><br>250mg/m^2 weekly over 12 weeks (adjuvant treatment)	Median follow-up 44,6 mo	Primary endpoint:<br>**1-year LC**<br>59% vs. 47%; *p= 0,25*<br><br>Secondary endpoints:<br>**RR** *at 12 weeks after treatment*<br>Overall response 85% vs. 96%; *p= 0,073*<br>**2-year LC**<br>44% vs. 44%<br>**3-year LC**<br>38% vs. 37%<br>**Median-DFS**<br>35,3 mo vs. 41 mo; *p=0,94*<br>**Event free survival**<br>18,4 mo vs. 23,7 mo; *p= 0,48*<br>**Median-OS**<br>33,6 mo vs. 39,9 mo; *p= 0,44*<br>**Toxicity (Grade 4)**<br>Mucositis 2% vs. 7%<br>Skin 2% vs. 0%	Feasible treatment option, no survival benefit over definitive treatment<br><br>adjuvant group had a bit more infections during adjuvant cetuximab

								Radiation dermatitis 2% vs. 0%	
**Nagpal et al. [22]**	Clin Cancer Investig J, 2021	N = 50 randomized/ 49 treated<br><br>Mean age: 56 (42-76)<br><br>10,2% female	SCCHN of **oropharynx %** **Hypopharynx %** **oral cavity %** **larynx %**<br><br>**Stage III / IV**<br>Stage III: 92%<br>Stage IV: 8%	Arm A:<br>N= 24<br>Carboplatin + Gefitinib + RT<br><br>Arm B:<br>N= 25<br>Carboplatin + Erlotinib + RT	66 Gy / 2 Gy<br>5 fractions per week	Carboplatin: AUC 2<br><br>Gefitinib: 250 mg OD<br><br>Erlotinib: 250 mg OD	Median follow-up 2 years (17-31 mo)	Primary endpoint:<br>**Complete RR**<br>At 3 mo post-RT<br>70,8% vs. 48%; *p= 0,07*<br>1-year post-RT<br>41,6% vs. 28%; *p= 0,18*<br>3 mo residual disease<br>4,1% vs. 33,1%<br>6 mo residual disease<br>25% vs. 9%<br>**DFS**<br>1-year DFS<br>41,6% vs. 29,1%<br>2-year DFS<br>33,3% vs. 25%<br><br>Secondary endpoints:<br>**2-year OS**<br>54,1% vs. 58,3%; *p= 0,38*<br>**Toxicity**<br>Deaths 0% vs. 8%<br>Skin (Grade 3) 21% vs. 37,5%<br>Mucositis 87,5% vs. 91,6%	
**Rischin et al. [23] TROG 12.01**	Int J Radiat Oncol Biol Phys, 2021	N = 189<br><br>Median age: 57,4<br><br>10% female	SCCHN of **oropharynx**<br>base of tongue 35%<br>posterior pharyngeal 1%<br>right tonsil, right lateral pharyngeal wall & right vallecula 1%<br>tonsil 62%<br>tonsil & base of tongue 2%<br><br>**Stage III / IV**<br>Stage III: 8%	Arm A:<br>N= 96<br>Cisplatin + IMRT<br><br>Arm B:<br>N= 93<br>Cetuximab + IMRT	70 Gy / 2 Gy<br><br>5 fractions per week	Cisplatin: 40 mg/m^2 weekly<br><br>Cetuximab: loading dose of 400 mg/m^2, 250 mg/m^2 during radiation	Median follow-up 4,1 years	Primary endpoint:<br>**Symptom severity**<br>difference in AUC Cetuximab vs. Cisplatin 0,05; *p= 0,66*<br>Modified symptom severity *p= 0,97*<br>Symptom interference score *p= 0,071*<br>Mucositis symptoms *p=0,91*<br>Common symptoms *p=0,92*<br><br>Secondary endpoints:	CTX similar symptom severity while less overall efficacy<br>No difference in OS, due to low-risk population<br>Slight imbalance between arms (CTX > CDDP) regarding pack years/patient

			Stage IV: 92%					**3-year failure free survival**<br>93% vs. 80%; *HR 3,0; p=0,015*<br>**3-year OS**<br>98% vs. 96%; *HR 2,3; p=0,32*<br>**3-year LC**<br>98% vs. 92%; *HR 3,4; p=0,11*<br>**3-year distant failure free survival**<br>97% vs. 88%; *HR 4,1; p= 0,018*<br>**AE**<br>Radiation dermatitis, acneiform rash -> more in CTX (T-score 3,82)<br>Febrile neutropenia, emesis, dry mouth, fatigue -> more in CDDP (T-score 4,35)<br>*p= 0,108*	
**Rodriguez et al. [24]**	Cancer Biol Ther, 2010	N = 106 randomized/ 105 treated<br><br>Median age Group A: 59<br>Median age Group B: 65<br><br>22,6 % female	SCCHN of<br>**Tonsil** 38,6%<br>**Base of tongue** 18,9%<br>**Alveolar ridge** 0,9%<br>**Retromolar trigone** 9,4%<br>**Hypopharynx** 0,9%<br>**Larynx** 0,9%<br>**Anterior tongue** 5,7%<br>**Oral mucosa** 2,8%<br>**Soft palate** 10,4%<br>**Hard palate** 3,8%<br>**Pharyngeal wall** 1,9%<br>**Maxillary sinus** 1,9%<br>**Floor of mouth** 3,9%<br><br>**Stage III / IV**<br>Stage III: 39,6%	<u>Arm A:</u><br>N= 54<br>Nimotuzumab + RT<br><br><u>Arm B:</u><br>N= 51<br>Placebo + RT	60-66 Gy/ 2 Gy<br><br>5 days per week	Nimotuzumab 200mg administered 6x during treatment<br><br>Placebo administered 6x during treatment	Median follow-up 45,2 mo	<u>Primary endpoint:</u><br>**Complete RR**<br>A vs. B: 59,5% vs. 34,2%<br>*p= 0,028*<br><br><u>Secondary endpoints:</u><br>**Immunogenicity**<br>Positive idiotypic response 10,7% vs. 4,2%<br>**Median OS**<br>12,5mo vs. 9,47mo<br>**Mean OS**<br>22,71mo vs. 17,71mo<br>**AE/ safety**<br><u>Overall:</u> 70,37% vs. 57,69%<br><u>Drug related (group A):</u> 31,48%<br><u>Mucositis:</u> 20,1% vs. 16,8%<br><u>Dry mouth:</u> 17% vs. 23%<br><u>Dry radio dermatitis:</u> 10,3% vs. 12,1%	Slightly delayed survival benefit for nimotuzumab group

			Stage IV: 60,4%					<u>Odynophagia:</u> 8% vs. 11,3%	
**Ruo Redda et al. [25]**	Tumori, 2010	N = 164 randomized/ 157 treated<br><br>Median age: 60<br><br>10% female	SCCHN of<br>**oropharynx** 56%<br>**Hypopharynx** 10%<br>**oral cavity** 19%<br>**larynx** 15%<br><br>**Stage III / IV**<br>Stage III: 22,8%<br>Stage IV: 77,2%	<u>Arm A:</u><br>N= 77<br>RT<br><br><u>Arm B:</u><br>N= 80<br>Carboplatin + RT	70 Gy / 2 Gy<br><br>5 fractions per week	Carboplatin 45 mg/m^2 on days 1-5, at weeks 1,3,5,7	Max. 10 years<br><br>Median follow-up 26,2 mo<br><br>Median observation period for surviving patients 154,3 mo	<u>Primary endpoint:</u><br>**Locoregional recurrence free survival**<br>**3-year:** 15% vs. 21,7%<br>**5-year:** 10,7% vs. 15,1%<br>**10-year:** 10,7% vs. 15,1%<br>*p= 0,11*<br><br><u>Secondary endpoints:</u><br>**DFS**<br>**3-year:** 9% vs. 16%<br>**5-year:** 5,5% vs. 6,8%<br>**10-year:** 5,5% vs. 6,8%<br>*p= 0,09*<br>**OS**<br>**3-year:** 11,1% vs. 28,9%<br>**5-year:** 6,9% vs. 9%<br>**10-year:** 6,9% vs. 5,5%<br>*p= 0,02*<br>**RR**<br>CR 23,5% vs. 46,5%; *p= 0,015*<br>PR 62,5% vs. 42,5%<br>PD 13% vs. 11%<br>**Toxicity**<br>Leukopenia: 0% vs. 9,5%<br>Anemia: 0% vs. 4%<br>Mucositis: 9,5% vs. 13%; *p= 0,2*	Carboplatin could not improve outcome
**Semrau et al. [26]**	Int J Radiat Oncol Biol Phys, 2006	N = 263<br><br>Median age: 57 (28-73)<br><br>15% female	SCCHN of<br>**oropharynx** 74%<br>**Hypopharynx** 26%<br><br>**Stage III / IV**<br>Stage III: 4%<br>Stage IV: 96%	<u>Arm A:</u><br>N= 127<br>RT<br><br><u>Arm B:</u><br>N= 113<br>Carboplatin + 5-FU + RT	69,9 Gy / 1,8 Gy<br><br>5 fractions per week<br><br><u>Concomitant boost concept:</u><br>1,5 Gy at least 6h after first dose in the last 2,5 weeks of treatment	Carboplatin: 70 mg/m^2<br><br>5-FU: 600 mg/m^2 days 1-5 and 29-33	Median follow-up 57,3 mo	<u>Endpoints:</u><br>**5-year LC**<br>12,6% vs. 22,7%; *p= 0,01*<br>**5-year OS**<br>15,8% vs. 25,6%; *p=0,016*<br>**Distant metastases**<br>0,7% vs. 3,5% still alive<br>22,8% vs. 18,6% overall<br>**AE**	High number of patients still dependent on feeding tube 5 years post-treatment/ till death<br><br>Patients with Hypopharynx

								Xerostomia 90,6% vs. 87,6%<br>Sense of taste 81,9% vs. 78,8%<br>Lymph edema 79,55 vs. 72,6%<br>Skin induration 68,5% vs. 62,8%<br>Skin pigmentation 66,1% vs. 61,1%<br>Skin fibrosis 25,2% vs. 31%<br>Hearing impairment 11,8% vs. 11,5%<br>Skin ulcers 7,9% vs. 7,1%<br>Osteoradionecrosis 5,5% vs. 8,8%	SCCHN did not benefit from CRT
**Siu et al. [27] HN.6**	JAMA Oncol, 2017	N = 320 randomized/ 315 treated<br><br>Median age: 56<br><br>16% female	SCCHN of **oropharynx** 81% **Hypopharynx** 6% **oral cavity** 2% **larynx** 11%<br><br>**Stage III / IV**<br>Stage T1-3: 83%<br>Stage T4: 17%	Arm A:<br>N= 156<br>Cisplatin + RT<br><br>Arm B:<br>N= 159<br>Panitumumab + RT	**Arm A:** 70 Gy / 2 Gy 5 fractions per week<br><br>**Arm B:** 70 Gy / 2 Gy 6 fractions per week (one day twice)	Cisplatin 100mg/m^2 on days 1, 22, 43<br><br>Panatimumab 9mg/kg every 3 weeks, starting one week before RT (days -7, 15, 36)	Median follow-up 46 mo	Primary endpoint:<br>**2-year PFS**<br>73% vs. 76%; *HR 0,95; p= 0,83*<br><br>Secondary endpoints:<br>**2-year OS**<br>85% vs. 88%; *HR 0,89; p= 0,66*<br>**2-year local recurrence**<br>4,5% vs. 7%; *HR 2; p= 0,2*<br>**2-year distant recurrence**<br>10,2% vs. 10,1%; *HR 1,01; p= 0,97*<br>**QoL**<br>-1,7 vs. -4,81; *p= 0,19*<br>**AE**<br>Acne 1% vs. 92%<br>Dehydration 29% vs. 24%<br>Dermatitis 97% vs. 90%<br>Dysphagia 92% vs. 94%<br>Fatigue 79% vs. 77%<br>Hearing loss 49% vs. 16%<br>Mucositis 77% vs. 75%<br>Nausea 87% vs. 59%	

								Pain (throat, pharynx, larynx) 52% vs. 54%<br>Renal failure 4% vs. 1%<br>Tinnitus 63% vs. 11%<br>Vomiting 56% vs. 40%<br>Weight loss 99% vs. 92%<br>Hemoglobin decrease 98% vs. 73%<br>Febrile neutropenia 4% vs. 0%<br>Neutropenia 67% vs. 13%<br>Platelet decrease 49% vs. 15%<br>Hypokalemia 35% vs. 26%<br>Hypomagnesemia 40% vs. 41%<br>Hyponatremia 75% vs. 43%<br>Feeding tube dependent (6 mo) 6% vs. 13%	
**Tao et al. [28] GORTEC 2007-01**	J Clin Oncol, 2018	N = 406<br><br>Mean age: 57 (36-70)<br><br>16% female	SCCHN of<br>**oropharynx** 65%<br>**Hypopharynx** 15%<br>**oral cavity** 12%<br>**larynx** 8%<br><br>**Stage III / IV**<br>Stage T0-T2: 21%<br>Stage T3: 28%<br>Stage T4: 30%	Arm A:<br>N= 202<br>Cetuximab + RT<br><br>Arm B:<br>N= 204<br>Cetuximab + Carboplatin + RT	70 Gy / 2 Gy<br>5 fractions per week<br><br>IMRT	Cetuximab: loading dose of 400 mg/m^2, 250 mg/m^2 during radiation<br><br>3 cycles Carboplatin 70 mg/m^2 on days 1-4<br><br>3 cycles of 5-FU 600 mg/m^2 on days 1-4	Median follow-up Arm A: 4,6 years<br><br>Median follow-up Arm B: 4,4 years	Primary endpoint:<br>**3-year PFS**<br>A= 40.5% vs. B= 52.3%; *HR 0.73; p= 0,015*<br>**Median PFS**<br>A= 22,4 mo vs. B= 37,9 mo<br><br>Secondary endpoints:<br>**Toxicity**<br>Radiodermatitis 96% vs. 97%<br>Skin reaction outside RT-field 13% vs. 12%<br>Mucositis 97% vs. 99%<br>WBC count 0% vs. 47%<br>Renal function 0% vs. 6%<br>Liver enzyme increase 18% vs. 33%<br>**3-year OS**<br>54,9% vs. 60,8%; *HR 0,8; p= 0,11*<br>**Median OS**	PFS increased significant with regimen of Arm B

								44,5 mo vs. 53,4 mo<br>**Locoregional failure**<br>38,8% vs. 21,6%; *HR 0,54; p< 0,001*<br>**Distant failure**<br>HR 1,19; *p= 0,5*<br>**Death without earlier progression**<br>HR 1,11; *p= 0,71*	
**Tao et al. [29] GORTEC 2015-01 PembroRad**	Ann Oncol, 2023	N = 133 randomized/ 131 treated<br><br>Median age Arm A: 65 (48-79)<br><br>Median age Arm B: 67 (47-81)<br><br>14,5% female	SCCHN of **oropharynx** 60,3% **Hypopharynx** 22,1% **oral cavity** 6,9% **larynx** 10,7%<br><br>**Stage III / IVB**<br>Stage III: 25,2%<br>Stage IVA: 55,7%<br>Stage IVB: 19,1%	<u>Arm A:</u><br>N= 66 Pembrolizumab + RT<br><br><u>Arm B:</u><br>N= 65 Cetuximab + RT	69,96 Gy / 2,12 Gy<br><br>5 fractions per week<br><br>IMRT	Pembrolizumab: 200mg on days 1, 22, 43 during RT<br><br>Cetuximab: loading dose of 400 mg/m^2 on day 8, 250 mg/m^2 weekly during radiation	Median follow-up Arm A: 25,6 mo<br><br>Median follow-up Arm B: 25,8 mo	<u>Primary endpoint:</u><br>**15-month LC**<br>60% vs. 59%; *p= 0,91*<br><br><u>Secondary endpoints:</u><br>**2-year PFS**<br>42,4% vs. 39,9%; *HR 0,83; p= 0,49*<br>**2-year Locoregional progression**<br>36,4% vs. 32,3%; *HR 1,04; p= 0,91*<br>**2-year Distant metastases**<br>12,1% vs. 15,4%; *HR 0,72; p= 0,5*<br>**2-year Deaths without cancer progression**<br>9,1% vs. 12,3%; *HR 0,8; p= 0,68*<br>**2-year OS**<br>61,7% vs. 55,3%; *HR 0,73 (95% CI: 0,42-1,26); p= 0,25*<br>**AE**<br><u>Grade 3 or worse:</u> 74% vs. 92%<br>Mucositis 32% vs. 58%<br>Radiodermatitis 20% vs. 59%<br>Rash 0% vs. 15%<br>Nutritional support 55% vs. 63%<br>Dysthyreodism 17% vs. 6%	No differences for oncological endpoints

**Thompson et al. [30]**<br>**NIMRAD**<br>**NCT01950689**	Int J Radiat Oncol Biol Phys, 2023	N = 338<br><br>Median age: 73 (45-84)<br><br>22,5% female	SCCHN of **oropharynx** 61,2%<br>**Hypopharynx** 15,1%<br>**larynx** 23,7%<br><br>**Stage II / IVB**<br>Stage II: 4,4%<br>Stage III: 30,5%<br>Stage IVA: 58,6%<br>Stage IVB: 6,5%	Arm A:<br>N= 170<br>Placebo + RT<br><br>Arm B:<br>N= 168<br>Nimorazole + RT	65 Gy / 2,17 Gy<br><br>5 fractions per week<br><br>IMRT	Nimorazole: 1,2 g/m^2<br><br>Placebo: 1,2 g/m^2	Median follow-up 3,1 years	Primary endpoint:<br>**Freedom from locoregional progression (FFLRP)**<br>HR 0,76; *p= 0,25*<br>**1-year FFLRP**<br>77% vs. 83%<br>**3-year FFLRP**<br>72% vs. 77%<br><br>Secondary endpoints:<br>**OS**<br>HR 0,92; *p= 0,66*<br>**1-year OS**<br>80% vs. 82%<br>**3-year OS**<br>60% vs. 60%<br>**CSS**<br>HR 0,92; *p= 0,72*<br>**DFS**<br>HR 1; *p= 0,98*<br>**AE**<br>Nausea Grade 1+2: 42,4% vs. 56.5%<br>Nausea Grade 3+4: 5,9% vs. 10.1%; *p< 0,05*<br>Grade 1+2 hoarseness 6,5% vs. 8,3%; *p< 0,05*	Nimorazole did not improve LC or survival significantly, but had worse AE

N (total) = 7.000 patients enrolled/ 6.814 patients treated

Abbreviations: *AE = adverse events; CRT = chemoradiotherapy; CSS = Cancer specific survival; DFS = disease free survival; FFLRP = Freedom from locoregional progression; LC = locoregional control; mo = months; OS = overall survival; QoL = Quality of life; RR = response rate; RT = radiotherapy*

1. Al-Saleh, K., et al., *Phase II/III Randomized Controlled Trial of Concomitant Hyperfractionated Radiotherapy plus Cetuximab (Anti-EGFR Antibody) or Chemotherapy in Locally Advanced Head and Neck Cancer.* Gulf journal of oncology, 2019. **1**(30): p. 6-12.
2. Argiris, A., et al., *Phase II randomized trial of radiation therapy, cetuximab, and pemetrexed with or without bevacizumab in patients with locally advanced head and neck cancer.* Annals of oncology : official journal of the european society for medical oncology, 2016. **27**(8): p. 1594-1600.
3. Bonner, J.A., et al., *Radiotherapy plus cetuximab for squamous-cell carcinoma of the head and neck.* New England journal of medicine, 2006. **354**(6): p. 567-578.

4. Bourhis, J., et al., *Concomitant chemoradiotherapy versus acceleration of radiotherapy with or without concomitant chemotherapy in locally advanced head and neck carcinoma (GORTEC 99-02): an open-label phase 3 randomised trial.* (1474-5488 (Electronic)).
5. Budach, V., et al., *Hyperfractionated accelerated chemoradiation with concurrent fluorouracil-mitomycin is more effective than dose-escalated hyperfractionated accelerated radiation therapy alone in locally advanced head and neck cancer: final results of the radiotherapy cooperative clinical trials group of the German Cancer Society 95-06 Prospective Randomized Trial.* Journal of clinical oncology, 2005. **23**(6): p. 1125-1135.
6. Budach, V., et al., *Hyperfractionated accelerated radiation therapy (HART) of 70.6 Gy with concurrent 5-FU/Mitomycin C is superior to HART of 77.6 Gy alone in locally advanced head and neck cancer: long-term results of the ARO 95-06 randomized phase III trial.* International journal of radiation oncology, biology, physics, 2015. **91**(5): p. 916-924.
7. Chitapanarux, I., et al., *Randomized phase III trial of concurrent chemoradiotherapy vs accelerated hyperfractionation radiotherapy in locally advanced head and neck cancer.* 2013(1349-9157 (Electronic)).
8. Essa, H.H. and M. Azzam, *Concurrent chemoradiation in locally advanced head and neck cancers: a comparative study of weekly Paclitaxel versus Cisplatin-based regimen.* J Egypt Natl Canc Inst, 2010. **22**(3): p. 165-73.
9. Ezzat, M., et al., *A randomized study of accelerated fractionation radiotherapy with and without mitomycin C in the treatment of locally advanced head and neck cancer.* 2005(1110-0362 (Print)).
10. Fallai, C., et al., *Long-term results of conventional radiotherapy versus accelerated hyperfractionated radiotherapy versus concomitant radiotherapy and chemotherapy in locoregionally advanced carcinoma of the oropharynx.* Tumori, 2006. **92**(1): p. 41-54.
11. Olmi, P., et al., *Locoregionally advanced carcinoma of the oropharynx: conventional radiotherapy vs. accelerated hyperfractionated radiotherapy vs. concomitant radiotherapy and chemotherapy--a multicenter randomized trial.* (0360-3016 (Print)).
12. Gebre-Medhin, M., et al., *ARTSCAN III: a Randomized Phase III Study Comparing Chemoradiotherapy With Cisplatin Versus Cetuximab in Patients With Locoregionally Advanced Head and Neck Squamous Cell Cancer.* Journal of clinical oncology, 2021. **39**(1): p. 38-47.
13. Geoffrois, L., et al., *Induction chemotherapy followed by cetuximab radiotherapy is not superior to concurrent chemoradiotherapy for head and neck carcinomas: results of the GORTEC 2007-02 Phase III Randomized Trial.* Journal of clinical oncology, 2018. **36**(31): p. 3077-3083.
14. Gillison, M.L., et al., *Radiotherapy plus cetuximab or cisplatin in human papillomavirus-positive oropharyngeal cancer (NRG Oncology RTOG 1016): a randomised, multicentre, non-inferiority trial.* Lancet (london, england), 2019. **393**(10166): p. 40-50.
15. Giralt, J., et al., *Panitumumab plus radiotherapy versus chemoradiotherapy in patients with unresected, locally advanced squamous-cell carcinoma of the head and neck (CONCERT-2): a randomised, controlled, open-label phase 2 trial.* (1474-5488 (Electronic)).
16. Halim, A.A.F., et al., *Concomitant chemoradiotherapy using low-dose weekly gemcitabine versus low-dose weekly paclitaxel in locally advanced head and neck squamous cell carcinoma: A phase III study.* Medical Oncology, 2012. **29**(1): p. 279-284.
17. Magrini, S.M., et al., *Cetuximab and Radiotherapy Versus Cisplatin and Radiotherapy for Locally Advanced Head and Neck Cancer: a Randomized Phase II Trial.* Journal of clinical oncology, 2016. **34**(5): p. 427-435.
18. Mehanna, H., et al., *Radiotherapy plus cisplatin or cetuximab in low-risk human papillomavirus-positive oropharyngeal cancer (De-ESCALaTE HPV): an open-label randomised controlled phase 3 trial.* Lancet (london, england), 2019. **393**(10166): p. 51-60.

19. Mell, L.K., et al., *Radiotherapy with cetuximab or durvalumab for locoregionally advanced head and neck cancer in patients with a contraindication to cisplatin (NRG-HN004): an open-label, multicentre, parallel-group, randomised, phase 2/3 trial.* Lancet Oncol, 2024. **25**(12): p. 1576-1588.
20. Mercke, C., et al., *Radiotherapy-Dose Escalated for Large Volume Primary Tumors-And Cetuximab with or without Induction Chemotherapy for HPV Associated Squamous Cell Carcinoma of the Head and Neck-A Randomized Phase II Trial.* Cancers, 2023. **15**(9): p. 13.
21. Mesía, R., et al., *Adjuvant therapy with cetuximab for locally advanced squamous cell carcinoma of the oropharynx: results from a randomized, phase II prospective trial.* Annals of oncology : official journal of the european society for medical oncology, 2013. **24**(2): p. 448-453.
22. Nagpal, P., et al., *Comparison of concurrent chemoradiation with daily gefitinib versus daily erlotinib in locally advanced oropharyngeal cancers.* Clinical cancer investigation journal, 2021. **10**(4): p. 203-208.
23. Rischin, D., et al., *Randomized trial of radiation therapy with weekly cisplatin or cetuximab in low-risk hpv-associated oropharyngeal cancer (TROG 12.01) - a trans-Tasman radiation oncology group study.* International journal of radiation oncology biology physics, 2021. **111**(4): p. 876-886.
24. Rodríguez, M.O., et al., *Nimotuzumab plus radiotherapy for unresectable squamous-cell carcinoma of the head and neck.* Cancer biology & therapy, 2010. **9**(5): p. 343-349.
25. Ruo Redda, M.G., et al., *Radiotherapy alone or with concomitant daily low-dose carboplatin in locally advanced, unresectable head and neck cancer: definitive results of a phase III study with a follow-up period of up to ten years.* Tumori, 2010. **96**(2): p. 246-253.
26. Semrau, R., et al., *Efficacy of intensified hyperfractionated and accelerated radiotherapy and concurrent chemotherapy with carboplatin and 5-fluorouracil: updated results of a randomized multicentric trial in advanced head-and-neck cancer.* Int J Radiat Oncol Biol Phys, 2006. **64**(5): p. 1308-16.
27. Siu, L.L., et al., *Effect of Standard Radiotherapy With Cisplatin vs Accelerated Radiotherapy With Panitumumab in Locoregionally Advanced Squamous Cell Head and Neck Carcinoma: A Randomized Clinical Trial.* 2017(2374-2445 (Electronic)).
28. Tao, Y., et al., *Improved outcome by adding concurrent chemotherapy to cetuximab and radiotherapy for locally advanced head and neck carcinomas: results of the GORTEC 2007-01 Phase III Randomized Trial.* Journal of clinical oncology, 2018. **36**(31): p. 3084-3090.
29. Tao, Y., et al., *Pembrolizumab versus cetuximab concurrent with radiotherapy in patients with locally advanced squamous cell carcinoma of head and neck unfit for cisplatin (GORTEC 2015-01 PembroRad): a multicenter, randomized, phase II trial.* Annals of oncology : official journal of the european society for medical oncology, 2023. **34**(1): p. 101-110.
30. Thompson, D.J., et al., *A Randomised Placebo-controlled Trial of Synchronous NIMorazole Versus RADiotherapy Alone in Patients With Locally Advanced Head and Neck Squamous Cell Carcinoma Not Suitable for Synchronous Chemotherapy or Cetuximab).* Clinicaltrials.gov [www.clinicaltrials.gov], 2013.
