# Supplementary material for: Non-Cisplatin Concurrent Systemic Therapy with Radiotherapy for Locally Advanced Head and Neck Squamous Cell Carcinoma: A Network Meta-Analysis of Randomized Clinical Trials
Source: Cancers (Basel). 2026 May 14;18(10):1599. doi: 10.3390/cancers18101599 (PMC13204043; doi:10.3390/cancers18101599)
Supplement: Supplementary file 1 [file cancers-18-01599-s001.zip › cancers-4313239-supplementary/Supplementary material 5.pdf]

Supplementary material 4: Table of studies excluded after full-text screening

**Excluded studies**	**Reason for exclusion**
Heukelom et al. [1]	Excluded since only the abstract was retrievable
Tao et al. [2-4]	Excluded since only safety phase of trial published and not all endpoints met
Tao et al. [5]	Pooled analysis, one trial did not match inclusion criteria, second included separately
Bourhis et al. [6]	Excluded since only the abstract was published
Jensen et al. [7]	Excluded because it was a single arm study and therefore did not meet inclusion criteria
Zhang et al. [8]	Excluded because article discusses possible study design, therefore did not meet inclusion criteria
He et al. [9]	Excluded since only the abstract was published
McBride et al. [10]	Excluded because patients with HNSCC and NPC were included in study
Lei et al. [11]	Excluded since only the abstract was published
Venkateshulu et al. [12, 13]	Excluded since it did not report overall survival and therefore did not meet inclusion criteria
Vias et al. [14]	Excluded since it did not report overall survival and therefore did not meet inclusion criteria
Tamaskovics et al. [15]	Excluded since only the abstract was retrievable
Bourhis et al. [16]	Excluded since article discusses possible study design and therefore did not meet inclusion criteria
Yom et al. [17]	Excluded since only the abstract was retrievable
Mell et al. [18]	Excluded since only the abstract was retrievable
Patil et al. [19]	Excluded because adjuvant concept was partially used and therefore did not meet inclusion criteria
Reddy et al. [20]	Excluded because no randomization was used in the first step between CRT/RT

Ghi et al. [21]	Phase 1 study
Babu et al. [22]	Excluded since only the abstract was retrievable
Liu et al. [23]	Excluded because study is ongoing, no results yet
EU Clinical Trials Register [24]	Excluded because study was terminated due to toxicity and no results posted
EU Clinical Trials Register [25]	Excluded because no results were retrievable
EU Clinical Trials Register [26]	Excluded because no results were retrievable
Haddad et al. [27]	Excluded because no results were retrievable
Iranian Registry of Clinical Trials [28]	Excluded because study is ongoing, no results yet
Klinghammer et al. [29]	Excluded because no results were retrievable
Skinner et al. [30]	Excluded because no results were retrievable
Wilke et al. [31]	Excluded because study is ongoing, no results yet
Groupe Oncologie Radiotherapie Tete et Cou [32]	Excluded because study is ongoing, no results yet
Mell et al. [33]	Excluded because study is ongoing, no results yet
Institut Claudius Regaud [34]	Excluded because study is ongoing, no results yet
Harari et al. [35]	Excluded because study is ongoing, no results yet
Johnson & Johnson Enterprise Innovation Inc. [36]	Excluded because study is ongoing, no results yet
Sher et al. [37]	Excluded because study is ongoing, no results yet
Hu et al. [38]	Excluded because study is retrospective and therefore did not meet inclusion criteria
Tang et al. [39]	Excluded because study used no randomization and therefore did not meet inclusion criteria

Baselga et al. [40]	Excluded because study used no control group and therefore did not meet inclusion criteria
Pignon et al. [41]	Excluded because meta-analysis compared neoadjuvant, concurrent and adjuvant concept, while using cisplatin in all three regimens and therefore did not meet inclusion criteria
Chauhan et al. [42]	Phase 1 study
Guan et al. [43]	Excluded because NPC included and therefore did not match inclusion criteria
Caroline et al. [44]	Cost analysis did not match inclusion criteria
Levy et al. [45]	Retrospective study
Hamed et al. [46]	Excluded because patients with HNSCC and NPC were included in study
Jain et al. [47]	Excluded because study used no randomization and therefore did not meet inclusion criteria
Salama et al. [48]	Excluded because patients with HNSCC and NPC were included in study
Tobias et al. [49]	Excluded because patients with HNSCC and NPC were included in study
Janssens et al. [50]	Excluded because no chemoradiotherapy/ immunoradiotherapy/ chemoimmunoradiotherapy was applied and therefore did not meet inclusion criteria
Ghi et al. [51]	Excluded because study used induction chemotherapy in both treatment arms and therefore did not meet inclusion criteria

1. Heukelom, J., et al., *Adaptive and innovative Radiation Treatment FOR improving Cancer treatment outcomE (ARTFORCE); a randomized controlled phase II trial for individualized treatment of head and neck cancer.* BMC cancer, 2013. **13**: p. 84.
2. Tao, Y., et al., *Avelumab–cetuximab–radiotherapy versus standards of care in locally advanced squamous-cell carcinoma of the head and neck: The safety phase of a randomised phase III trial GORTEC 2017-01 (REACH).* European Journal of Cancer, 2020. **141**: p. 21-29.
3. Tao, Y., et al., *Avelumab-cetuximab-radiotherapy versus standards of care (SoC) in patients (pts) with locally advanced squamous cell carcinoma of head and neck (LA-SCCHN): safety phase of randomized trial GORTEC 2017-01 (REACH).* Annals of oncology, 2019. **30**: p. v454.

4. Tao, Y., et al., *Avelumab-cetuximab-radiotherapy (RT) versus standards of care (SoC) in locally advanced squamous cell carcinoma of the head and neck (SCCHN): safety phase of the randomized trial GORTEC 2017-01 (REACH).* Journal of clinical oncology, 2018. **36**(15).
5. Tao, Y., et al., *Very accelerated radiotherapy or concurrent chemoradiotherapy for N3 head and neck squamous cell carcinoma: pooled analysis of two GORTEC randomized trials.* Oral oncology, 2017. **71**: p. 61-66.
6. Bourhis, J., et al., *LBA35 Avelumab-cetuximab-radiotherapy versus standards of care in patients with locally advanced squamous cell carcinoma of head and neck (LA-SCCHN): randomized phase III GORTEC-REACH trial.* Annals of oncology, 2021. **32**(Suppl 5): p. S1310.
7. Jensen, A.D., et al., *Radiochemoimmunotherapy with intensity-modulated concomitant boost: interim analysis of the REACH trial.* Radiation Oncology, 2012. **7**: p. 9.
8. Zhang, Q.E., et al., *Randomized phase II/III confirmatory treatment selection design with a change of survival end points: statistical design of Radiation Therapy Oncology Group 1216.* Head & neck, 2019. **41**(1): p. 37-45.
9. He, X., et al., *Raltitrexed versus 5-fluorouracil with cisplatin and concurrent radiotherapy (CCRT) for locally advanced head and neck squamous cell carcinoma (LA-HNSCC): a randomized controlled multi-centered trial.* Annals of oncology, 2017. **25**(Suppl 5): p. v372-v394.
10. McBride, S.M., et al., *A phase II randomized trial of nivolumab with stereotactic body radiotherapy (SBRT) versus nivolumab alone in metastatic (M1) head and neck squamous cell carcinoma (HNSCC).* Journal of clinical oncology, 2018. **36**(15).
11. Lei, Q., et al., *CAMRESBRT: randomized Phase II Trial of Camrelizumab with Stereotactic Body Radiotherapy vs. Camrelizumab Alone in Recurrent or Metastatic Head and Neck Squamous Cell Carcinoma.* International journal of radiation oncology biology physics, 2022. **114**(3): p. e282.
12. Venkateshulu, S. and B.R.K. Kumar, *A Study Comparing Acute Toxicities of Cetuximab and Cisplatin in Patients Undergoing Definitive Chemoradiation With Intensity-Modulated Radiotherapy for Locally Advanced Carcinoma Head and Neck.* Cureus Journal of Medical Science, 2021. **13**(7): p. 9.
13. Venkateshulu, S. and B.R. Kiran Kumar, *Intensity modulated radiotherapy with cisplatin or cetuximab in patients undergoing chemoradiation for squamous cell carcinoma head and neck.* Onkologia i radioterapia, 2021. **15**(7): p. 7-11.
14. Vias, P. and M. Gupta, *PO-133 CONCURRENT CHEMORADIATION IN HEAD AND NECK CANCERS COMPARING WEEKLY CISPLATIN VS ORAL HYDROXYUREA.* Radiotherapy and oncology, 2019. **141**: p. S57.
15. Tamaskovics, B.F., et al., *944TiP Randomized phase II study of immune stimulation with pembrolizumab and radiotherapy of recurrent and/or metastatic head and neck squamous cell carcinoma : the IMPORTANCE trial.* Annals of oncology, 2023. **34**: p. S593.
16. Bourhis, J., et al., *TrilynX: a phase 3 trial of xevinapant and concurrent chemoradiation for locally advanced head and neck cancer.* Journal of clinical oncology, 2021. **39**(15, Suppl).
17. Yom, S.S., et al., *NANORAY-312: a Phase III Pivotal Study of NBTXR3 Activated by Investigator's Choice of Radiotherapy Alone or Radiotherapy in Combination with Cetuximab for Platinum-Based Chemotherapy-Ineligible Elderly Patients with Locally Advanced HNSCC.* International journal of radiation oncology biology physics, 2022. **114**(3): p. e313.
18. Mell, L.K., et al., *Radiotherapy with Durvalumab vs. Cetuximab in Patients with Locoregionally Advanced Head and Neck Cancer and a Contraindication to Cisplatin: phase II Results of NRG-HN004.* International journal of radiation oncology biology physics, 2022. **114**(5): p. 1058.
19. Patil, V.M., et al., *Results of Phase III Randomized Trial for Use of Docetaxel as a Radiosensitizer in Patients With Head and Neck Cancer, Unsuitable for Cisplatin-Based Chemoradiation.* Journal of clinical oncology, 2023. **41**(13): p. 2350-2361.
20. Krishnamurthyreddy, B., et al., *A phase IIb 4-arm open-label randomized study to assess the safety and efficacy of h-R3 monoclonal antibody against EGFR in combination with*

chemoradiation therapy or radiation therapy in patients with advanced (stage III or IVA) inoperable head and neck cancer. Journal of clinical oncology, 2009. **27**(15, Suppl 1): p. 6041.
21. Ghi, M., et al., *Cetuximab/Radiation Therapy (CET+RT) Versus Concomitant Chemoradiation Therapy (cCHT+RT) With or Without Induction Docetaxel/Cisplatin/5Fluorouracil (TPF) in Locally Advanced Head-and-Neck Squamous Cell Carcinoma (LASCCHN) - Preliminary Results on Toxicity of a Randomized, 2x2 Factorial, Phase II-III Study (NCT01086826).* International journal of radiation oncology biology physics, 2012(3): p. S153-S153.
22. Babu, K.G., et al., *An open-label, randomized, study of h-R3mAb (nimotuzumab) in patients with advanced (stage III or IVa) squamous cell carcinoma of head and neck (SCCHN): four-year survival results from a phase IIb study.* Journal of clinical oncology, 2010. **28**(15, Suppl 1): p. Abstract 5530.
23. Liu, L., *Clinical study of PD-1 inhibitors combined with concurrent radiotherapy and chemotherapy in the treatment of locally advanced head and neck squamous cell carcinoma: a prospective, randomized, paralle controlled study.* https://trialsearch.who.int/Trial2.aspx?TrialID=ChiCTR2100046011, 2021.
24. EU Clinical Trials Register, *Randomized Phase IV Trial to Compare Cetuximab with Concomitant Radiation Therapy with Concomitant Mitomycin-C and 5-FU with Radiation Therapy for Locally Advanced Squamous Cell Carcinomas of The Head and Neck.* https://trialsearch.who.int/Trial2.aspx?TrialID=EUCTR2013-001296-20-AT, 2013.
25. EU Clinical Trials Register, *Open label randomized phase II, multicentre, pilot study to evaluate safety and efficacy of the combination of cetuximab and concomitant-boost accelerated radiotherapy followed or not by a complementary treatment with cetuximab in patients with locally advanced oropharynx squamous cell carcinoma.* EUCTR [www.clinicaltrialsregister.eu], 2005.
26. EU Clinical Trials Register, *A Phase 3 Study of Nivolumab or Nivolumab plus Cisplatin, in Combination with Radiotherapy in Patients with Advanced Cancer of the Head and Neck.* https://trialsearch.who.int/Trial2.aspx?TrialID=EUCTR2017-002676-87-PL, 2018.
27. Haddad, R., et al., *CheckMate 9TM: phase 3 study of nivolumab + radiotherapy (RT) vs cetuximab + RT in cisplatin-ineligible patients with intermediate-/high-risk locally advanced squamous cell carcinoma of the head/neck.* Journal for immunotherapy of cancer, 2018. **6**(Suppl 1).
28. Iranian Registry of Clinical Trials, *Evaluation the efficacy and safety of Cetuximab bio-similar compared to Erbitux in the patients with head and neck squamous cell carcinoma.* https://trialsearch.who.int/Trial2.aspx?TrialID=IRCT20190112042328N1, 2019.
29. Klinghammer, K.F., et al., *DURTRERAD: a phase II openlabel study evaluating feasibility and efficacy of durvalumab (D) and durvalumab and tremelimumab (DT) in combination with radiotherapy (RT) in non-resectable locally advanced HPV-negative HNSCC-Results of the preplanned feasibility interim analysis.* Journal of clinical oncology, 2020. **38**(15).
30. Skinner, H., *Randomized Phase II Trial of Stereotactic Body Radiation Therapy (SBRT) With Cetuximab +/- Docetaxel Followed by Adjuvant Cetuximab +/- Docetaxel in Recurrent, Previously-Irradiated Squamous Cell Carcinoma of the Head and Neck (SCCHN).* Clinicaltrials.gov [www.clinicaltrials.gov], 2013.
31. Wilke, C., *A Randomized, Phase II Study of Definitive Radiotherapy With Concurrent Cisplatin vs Docetaxel-cetuximab in Locally Advanced Head and Neck Squamous Cell Carcinoma: an ERCC1 Biomarker Enrichment and Interaction Design.* Clinicaltrials.gov [www.clinicaltrials.gov], 2014.
32. Groupe Oncologie Radiotherapie Tete et Cou, *Randomized trial of avelumab-cetuximab-radiotherapy versus SoCs in LA SCCHN (REACH).* https://clinicaltrials.gov/show/NCT02999087, 2016.
33. Mell, L., *Chemoradiation vs Immunotherapy and Radiation for Head and Neck Cancer.* https://clinicaltrials.gov/show/NCT03383094, 2017.

34. Institut Claudius Regaud, *Cisplatin or ImmunoTHerapy in Association With Definitive Radiotherapy in HPV-related oropharyngEal Squamous Cell Carcinoma: a Randomized Phase II Trial.* https://clinicaltrials.gov/show/NCT03623646, 2018.
35. Harari, P., *Testing Docetaxel-Cetuximab or the Addition of an Immunotherapy Drug, Atezolizumab, to the Usual Chemotherapy and Radiation Therapy in High-risk Head and Neck Cancer.* https://clinicaltrials.gov/show/NCT04411121, 2020.
36. Johnson & Johnson Enterprise Innovation Inc., *NBTXR3 With or Without Cetuximab in LA-HNSCC.* https://clinicaltrials.gov/show/NCT04892173, 2021.
37. Sher, D., *Combining Radiation Therapy With Immunotherapy for the Treatment of Metastatic Squamous Cell Carcinoma of the Head and Neck.* https://clinicaltrials.gov/show/NCT05721755, 2023.
38. Hu, M.H., et al., *Cisplatin-based chemotherapy versus cetuximab in concurrent chemoradiotherapy for locally advanced head and neck cancer treatment.* Biomed Res Int, 2014. **2014**: p. 904341.
39. Tang, C., et al., *Concurrent cetuximab versus platinum-based chemoradiation for the definitive treatment of locoregionally advanced head and neck cancer.* Head Neck, 2015. **37**(3): p. 386-92.
40. Baselga, J., et al., *Phase II multicenter study of the antiepidermal growth factor receptor monoclonal antibody cetuximab in combination with platinum-based chemotherapy in patients with platinum-refractory metastatic and/or recurrent squamous cell carcinoma of the head and neck.* 2005(0732-183X (Print)).
41. Pignon, J.P., et al., *Meta-analysis of chemotherapy in head and neck cancer (MACH-NC): an update on 93 randomised trials and 17,346 patients.* 2009(1879-0887 (Electronic)).
42. Chauhan, A., et al., *Gemcitabine concurrent with radiation therapy for locally advanced head and neck carcinomas.* 2008(1729-0503 (Electronic)).
43. Guan, J., et al., *A meta-analysis comparing cisplatin-based to carboplatin-based chemotherapy in moderate to advanced squamous cell carcinoma of head and neck (SCCHN).* 2016(1949-2553 (Electronic)).
44. Caroline, B., et al., *Cost analysis of cetuximab (Erbitux) plus radiotherapy (ERT) versus concomitant cisplatin plus radiotherapy (CRT) within an NHS oncology unit (single institution): a pilot study.* British journal of radiology, 2016. **89**(1068) (no pagination).
45. Levy, A., et al., *Concurrent use of cisplatin or cetuximab with definitive radiotherapy for locally advanced head and neck squamous cell carcinomas.* Strahlenther Onkol, 2014. **190**(9): p. 823-31.
46. Hamed, R.H. and E. Elzahaf, *Low dose weekly paclitaxel versus low dose weekly cisplatin with concomitant radiation in locally advanced head and neck cancers.* Journal of Cancer Science and Therapy, 2011. **3**(7): p. 168-172.
47. Jain, R.K., et al., *A comparative study of low dose weekly paclitaxel versus cisplatin with concurrent radiation in the treatment of locally advanced head and neck cancers.* Indian J Cancer, 2009. **46**(1): p. 50-3.
48. Salama, J.K., et al., *A randomized phase II study of 5-fluorouracil, hydroxyurea, and twice-daily radiotherapy compared with bevacizumab plus 5-fluorouracil, hydroxyurea, and twice-daily radiotherapy for intermediate-stage and T4N0-1 head and neck cancers.* Annals of oncology : official journal of the european society for medical oncology, 2011. **22**(10): p. 2304-2309.
49. Tobias, J.S., et al., *Chemoradiotherapy for locally advanced head and neck cancer: 10-year follow-up of the UK Head and Neck (UKHAN1) trial.* The lancet. Oncology, 2010. **11**(1): p. 66-74.
50. Janssens, G.O., et al., *Accelerated radiotherapy with carbogen and nicotinamide for laryngeal cancer: results of a phase III randomized trial.* 2012(1527-7755 (Electronic)).
51. Ghi, M.G., et al., *Cetuximab/radiotherapy (CET+RT) versus concomitant chemoradiotherapy (cCHT+RT) with or without induction docetaxel/cisplatin/5-fluorouracil (TPF) in locally advanced head and neck squamous cell carcinoma (LASCCHN): preliminary results on toxicity*

of a randomized, 2x2 factorial, phase II-III study (NCT01086826). Journal of clinical oncology, 2012. **30**(15).
