# Supplementary material for: Non-Cisplatin Concurrent Systemic Therapy with Radiotherapy for Locally Advanced Head and Neck Squamous Cell Carcinoma: A Network Meta-Analysis of Randomized Clinical Trials
Source: Cancers (Basel). 2026 May 14;18(10):1599. doi: 10.3390/cancers18101599 (PMC13204043; doi:10.3390/cancers18101599)
Supplement: Supplementary file 1 [file cancers-18-01599-s001.zip › cancers-4313239-supplementary/Supplementary material 6.pdf]

Supplementary material 5: Assessment of risk of bias according to "SIGN Methodology Checklist 2: Controlled Trials" and rating according to Oxford criteria

Author and Year	Section 1: internal validity										Section 2: overall assessment of the study				Oxford level
	1.1	1.2	1.3	1.4	1.5	1.6	1.7	1.8	1.9	1.10	2.1	2.2	2.3	2.4	
Al-Saleh et al.; 2019	Yes	Can't say	No	No	Yes	Yes	Yes	9% from arm A had a cross-over to arm B and were excluded from treatment outcome analysis, but still included in toxicity profile comparison	No	Does not apply	Acceptable (+)	Though study uses small sample size (was planned for more patients) studies with similar intervention but bigger sample size reported comparable results	Yes	Cetuximab-RT had worse outcome than cisplatin-RT. Hyperfractionation could not compensate this effect. It was no intention to treat analysis for oncologic outcomes conducted, although drop-out/cross-over rate was low. Randomization was mentioned, but not specified which method was used.	1b
Argiris et al.; 2016	Yes	Yes	No	No	Yes	Yes	Yes	2,5% were found ineligible to inclusion criteria after randomization and excluded prior to treatment	Yes	Yes	High quality (++)	Though study has limited sample size, other studies with different control groups reported comparable results for bevacizumab arm	Yes	Bevacizumab had similar survival compared to control arm of the study, while having worse adverse events. Study had no drop-outs, results between academic and community centers were comparable.	1b
Bonner et al.; 2006	Yes	Can't say	Yes	No	Yes	Yes	Yes	0,95% were randomized but received no treatment, 0,7% discontinued treatment after 1st dose of cetuximab without RT	Yes	Can't say	High quality (++)	Although results of centres were not compared, overall effect shown in trial is due to treatment	Yes	Significant survival advantage of cetuximab-RT compared to RT, while having moderate toxic adverse events. Still 5-year survival is low in both groups. Study did not compare overall results with results of single centers included in study. Patients were randomized but study did not report on method.	1b
Budach et al.; 2005	Yes	Yes	Can't say	No	Yes	Yes	Yes	2,3% dropped out before therapy<br>10% loss to follow-up in first 3 years	Yes	Can't say	High quality (++)	Study results align with other studies' findings.	Yes	No long-term late morbidity data (e.g. cause of non-cancer related deaths). To few events for proper competing risk analysis. No centre specific data reported.	1b

Essa et al.; 2010	Yes	Can't say	No	No	Yes	Yes	Yes	None of the patients randomized dropped out.	Yes	Does not apply	High quality (++)	The study reports results that are comparable to those of other studies.	Yes	Paclitaxel-RT had comparable response rates to Cisplatin-RT and seems an feasible alternative when Cisplatin is contraindicated. No randomization method specified.	1b
Fallai et al.; 2006	Yes	Yes	No	No	Yes	Yes	Yes	7% did not complete RT,<br>2,2% did not have planned CRT<br>(due to refusal/ missing data)	Yes	Can't say	High quality (++)	Study results are comparable to other studies investigating a similar regimen.	Yes	CRT results compared to RT alone did not reach statistical significance, but 5-year outcomes (OS, relapse-free survival and LC) were doubled compared to RT. Study did not specify whether results were comparable between all centers.	1b
Gebre-Medhin et al.; 2021	Yes	Yes	Yes	No	Yes	Yes	Yes	2,3% of patients were not available for evaluation of primary endpoint<br>3,7% of patients were not available for tumor response evaluation	Yes	Can't say	High quality (++)	Results of this study are comparable to other studies.	Yes	Patient inclusion was prematurely discontinued, primary endpoint did not reach significance. Concurrent cisplatin had significant better results than cetuximab, especially HPV-related cancer. No centre specific outcomes reported. Inclusion of more patients was early discontinued.	1b
Geoffrois et al.; 2018	Yes	Yes	Yes	No	Yes	Yes	Yes	2,7% of randomized patients dropped out, due to being not eligible. No cross-over reported	Yes	Can't say	High quality (++)	Study had lower RR than other studies evaluating TPF induction chemotherapy, but also restricted inclusion to more advanced stages, so overall effect is due to intervention	Yes	No benefit in PFS for induction TPF-regimen, but less distant metastases. TPF has still high toxicity.<br>No centre specific data was reported. Randomization was carried out and described in great detail.	1b

Gillison et al.; 2019	Yes	Yes	Yes	No	Yes	Yes	Yes	5,2% not included in analysis retrospectively, 6,4% lost to follow-up, 13,8% discontinued treatment	Yes	Can't say	Acceptable (+)	Other retrospective studies reported comparable results.	Yes	Cetuximab-RT had inferior outcome to ciplatin-RT. Cisplatin had higher rate of AE. No results reported specific for centre of treatment. High rate for treatment discontinuation, although patients included in final analysis.	1b
Giralt et al.; 2015	Yes	Yes	Yes	No	Yes	Yes	Yes	0,7% did not receive any study medication	Yes	Can't say	High quality (++)	Study results are comparable to other studies investigating concomitant application of EGFR-inhibitors with radiotherapy	Yes	5,2% of patients were declared ineligible retrospectively and removed from final analysis. Panatimumab-RT is not as effective as cisplatin-RT, while having more toxicity related AE and deaths. Study used computerized randomization and adequate concealment, but was open-label and did not report whether centre specific results were comparable.	1b
Halim et al.; 2012	Yes	Yes	Yes	No	Yes	Yes	Yes	No drop-outs	Yes	Can't say	High quality (++)	The reported results of this study are comparable to studies investigating a similar regimen.	Yes	Paclitaxel had better outcome than gemcitabine while being less toxic. Study reported no drop-outs, but also did not report whether results of their centres were comparable. Randomization with closed envelope was used.	1b
Magrini et al.; 2016	Yes	Yes	Yes	No	Yes	Yes	Yes	5,7% were not analyzed for toxicity and survival, because those patients discontinued treatment (death, treatment related AE)	Yes	Can't say	High quality (++)	Other trials report comparable outcomes regarding OS, MFS and LC. Adverse events have different profiles but are comparable in toxicity.	Yes	No significant differences between treatment outcome of both groups. Cisplatin had better outcome in LC, OS and cancer specific survival. Cetuximab had better outcome at MFS. AE were comparable in severity. No centre specific results reported.	1b

Mehanna et al.; 2019	Yes	Yes	Yes	No	Yes	Yes	Yes	2,4% withdrew prior to treatment	Yes	Can't say	High quality (++)	Prior non-randomized trials showed a comparable outcome.	Yes	Cetuximab had no benefit in toxicity, yet worse tumor control. Only toxicity profile differed between treatment groups. 8 patients withdrew prior to treatment, but one of them was still analysed. No centre specific data reported.	1b
Mercke et al.; 2023	Yes	Yes	Yes	No	Yes	Yes	Yes	1,3% withdrew from study,<br>2% were radiologically not evaluable	Yes	Can't say	High quality (++)	Results of this study are comparable with studies investigating the same entity with comparable treatment interventions.	Yes	Intensive RT improved outcome without increasing toxicity. TPF-IC reduced (not significant) distant metastases. Short period of follow-up is a limitation. Study did not report on a comparison of results of single centers. Open-label study.	1b
Mesia et al.; 2012	Yes	Can't say	No (None reported)	No	Yes	Yes	Yes	2,2% discontinued treatment of concomitant RT, another 9% dropped out before adjuvant cetuximab	Yes	Can't say	Low quality (-)	Study had small sample size and did not report on specific methodology for randomization, concealment and single center results.<br>However bigger studies with a comparable treatment had similar results	Yes	Adjuvant cetuximab did not improve long-term survival. Small sample size. No method for randomization reported, as well as concealment method. No comparison of results between single centers.	2b due to low quality
Nagpal et al.; 2021	Yes	Yes	No	No	Can't say	Yes	Yes	2% were lost to follow-up and excluded from study	No (per-protocol analysis)	Does not apply	Low quality (-)	Worse results than other trials evaluating erlotinib/gefitinib + RT, could be because of only concurrent application	Yes	No significant improvement in DFS and OS in both treatment arms. Small cohort and use of conventional RT. Simple randomization method.<br>Small sample size. No treatment specific characterization of study groups. Per-protocol analysis.	2b due to low quality

Rischin et al.; 2021	Yes	Yes	Yes	No	Yes	Yes	Yes	2,6% did not receive allocated treatment, 30,4% in CDDP arm discontinued/ were lost to follow-up 11,1% in CTX arm discontinued/ were lost to follow-up	Yes	Can't say	Acceptable (+)	Results comparable to two other trials (RTOG 1016 and De-ESCALaTE)	Yes	Cetuximab had less tumor control, while having comparable rate of AE. Patient reported symptom burden was comparable in both groups. Limited sample size could not show difference in OS, while a longer period of follow-up probably would be needed. Patients reported more depression symptoms in CDDP arm, while biased result due to open-label design or chance finding possible.High rate of discontinuation, especially in CDDP arm. No centre specific data reported.	2b due to <80% follow up
Rodriguez et al.; 2010	Yes	Yes	Yes	Yes	Yes	Yes	Yes	1% dropped out before treatment	Yes	Can't say	High quality (++)	Results align with previous results from other RCTs.	Yes	Significant survival benefit from treatment with nimotuzumab could be shown in delayed effects.<br>No center specific results reported.	1b
Ruo Redda et al.; 2010	Yes	Can't say	No (None reported)	No	Yes	Yes	Yes	4,6% were excluded after randomization	Yes	Does not apply	Acceptable (+)	Although the study could not show a beneficial outcome from combining carboplatin with RT, the reported effect is due to the intervention of the study	Yes	Carboplatin-RT could not improve outcome compared to RT. No randomization method specified. Long period of follow-up.	1b
Semrau et al.; 2006	Yes	Can't say	No	No	Yes	No (imbalance in randomization to G-CSF therapy)	Yes	9% did not start treatment	No (as-treated analysis)	No (one centre did not apply G-CSF as the others)	Low quality (-)	A lot of bias is likely contained, starting at randomization which was not further described, an as-treated analysis, and imbalanced additional treatment with G-CSF	Yes	5-year survival statistically significantly better. No significant benefit of LC. Randomization method not specified, as-treated analysis, imbalance in treatment with G-CSF between both groups (A and B). No centre specific data	2b due to low quality

												which was reported to have worse outcome in this study, as well as having a varying treatment schedule between one centre and the others (as it did not apply G-CSF to patients)		reported. Late toxicity was measured without grading according to common toxicity scales.	
Tao et al.; 2018	Yes	Yes	Yes	No	Yes	Yes	Yes	1,5% dropped out before treatment started;<br>3,2% received a different treatment than their assigned one	Yes	Can't say	High quality (++)	The results of this study are in line with many other studies investigation a comparable regimen.	Yes	Significant improvement of PFS and LC, insignificant better outcome in OS. Still, intensified treatment is more toxic.<br>No centre specific outcome data reported.	1b
Tao et al.; 2023	Yes	Yes	Yes	No	Yes	Yes	Yes	1,5% of patients dropped-out before treatment started;<br>0,75% lost to follow-up	Yes	Can't say	High quality (++)	Results comparable to another study investigating PD-1 inhibitors combined with RT.	Yes	Primary endpoint LC at 15 months not met (no differences). OS, PFS, locoregional progression and distant metastases also not different at 2-years. Pembrolizumab had less toxicity. No centre specific outcomes reported.	1b
Thompson et al.; 2023	Yes	Yes	Yes	Yes	Yes	Yes	Yes	3% did not start treatment;<br>6% dropped-out during treatment	Yes	Can't say	High quality (++)	Another study investigating nimorazole in SCCCHN plus RT reported comparable results.	Yes	No significant improvement of LC and survival with nimorazole. Study recruited mainly elderly (>70y) or comorbid patients. No reported data on single centre results.	1b
Bourhis et al.; 2012	Yes	Yes	Yes	No	Yes	Yes	Yes	2,6% had protocol violations<br>(death before treatment, off-protocol treatment, no concomitant CT, additional CT)	Yes	Can't say	High quality (++)	Results of this study are comparable with another study investigating acceleration plus CT.	Yes	CRT plus acceleration did not show significant benefit in PFS. Very accelerated RT was inferior to both CRT regimens considering outcome and toxicity. No centre specitic data reported.	1b
Siu et al.; 2017	Yes	Yes	Yes	No	Yes	Yes	Yes	1,6% withdrew consent or received therapy	Yes	Can't say	High quality (++)	Study results align with other studies' findings.	Yes	No significant better or worse outcome of bioradiotherapy vs.	1b

								different to treatment arm						chemoradiotherapy. No centre specific data reported.	
Ezzat et al.; 2005	Yes	Can't say	No (None reported)	No	Yes	Yes	Yes	No drop-outs	Yes	Does not apply	Acceptable (+)	Outcome of this study was a bit worse than comparable studies, but the included cancer stages were also more severe.Compliance in this trial was moderate, as patients delayed their treatment.	Yes	Statistically insignificant improvement of OS, while having significant worse acute toxicity. Late toxicity was not increased. Method used for randomization not specified. Small sample size in each arm.	1b
Chitapanarux et al.; 2013	Yes	Can't say	No	No	Yes	Yes	Yes	16,7% of the patients withdrew consent prior to treatment	No (as-treated analysis)	Does not apply	Low quality (-)	Other studies reported comparable outcomes.	Yes	Study was terminated early. No significant difference in LC, marginally improved OS. High hematologic toxicity. High drop-out rate. Method of randomization not specified. Small sample size due to early termination of study.	2b due to low quality
Mell et al.; 2024	Yes	Yes	Yes	No	Yes	Yes	Yes	12% lost to follow-up	Yes	Yes	High quality (++)	Yes, although this study is open-label, it is of good methodical quality and study results align with research data of other studies	Yes	Durvalumab did not increase efficacy or showed a favourable toxicity profile. Because of those results the trial did not enter phase 3. No long-time follow-up because trial closed early.	1b

Abbreviations: ***1.1*** *The study addresses an appropriate and clearly focused question;* ***1.2*** *The assignment of subjects to treatment groups is randomized;* ***1.3*** *An adequate concealment method is used;* ***1.4*** *The design keeps subjects and investigators 'blind' about treatment allocation;* ***1.5*** *The treatment and control groups are similar at the start of the trial;* ***1.6*** *The only difference between groups is the treatment under investigation;* ***1.7*** *All relevant outcomes are measured in a standard, valid and reliable way;* ***1.8*** *What percentage of the individuals or clusters recruited into each treatment arm of the study dropped out before the study was completed?;* ***1.9*** *All the subjects are analysed in the groups to which they were randomly allocated (often referred to as intention to treat analysis);* ***1.10*** *Where the study is carried out at more than one site, results are comparable for all sites;* ***2.1*** *How well was the study done to minimise bias?;* ***2.2*** *Taking into accout clinical considerations, your evaluation of the methodology used, and the statistical power of the study, are you certain that the overall effects is due to the study intervention?;* ***2.3*** *Are the results of this study directly applicable to the patient group targeted by this guideline?;* ***2.4*** *Notes: summarize the author's conclusions. Add any comments on your own assessment of the study, ant the extent to which it answers your question and mention any areas of uncertainty raised above.*
