# Supplementary figures and images for: Non-Cisplatin Concurrent Systemic Therapy with Radiotherapy for Locally Advanced Head and Neck Squamous Cell Carcinoma: A Network Meta-Analysis of Randomized Clinical Trials
Source: Cancers (Basel). 2026 May 14;18(10):1599. doi: 10.3390/cancers18101599 (PMC13204043; doi:10.3390/cancers18101599)

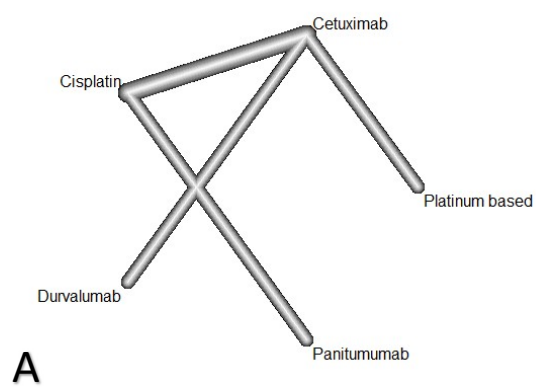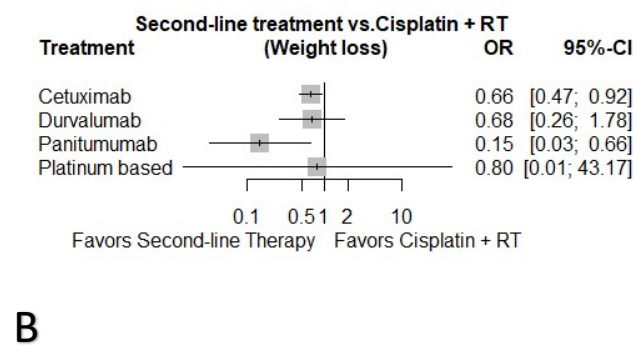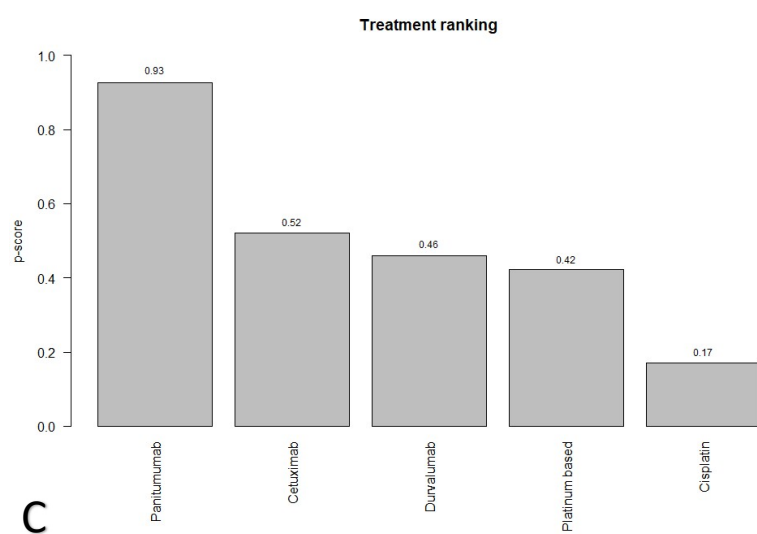

Supplementary material 10: Results for weight loss (A) netgraph, (B) forest plot, (C) treatment ranking

Supplement: Supplementary file 1 [file cancers-18-01599-s001.zip › cancers-4313239-supplementary/Supplementary material 11.pdf]

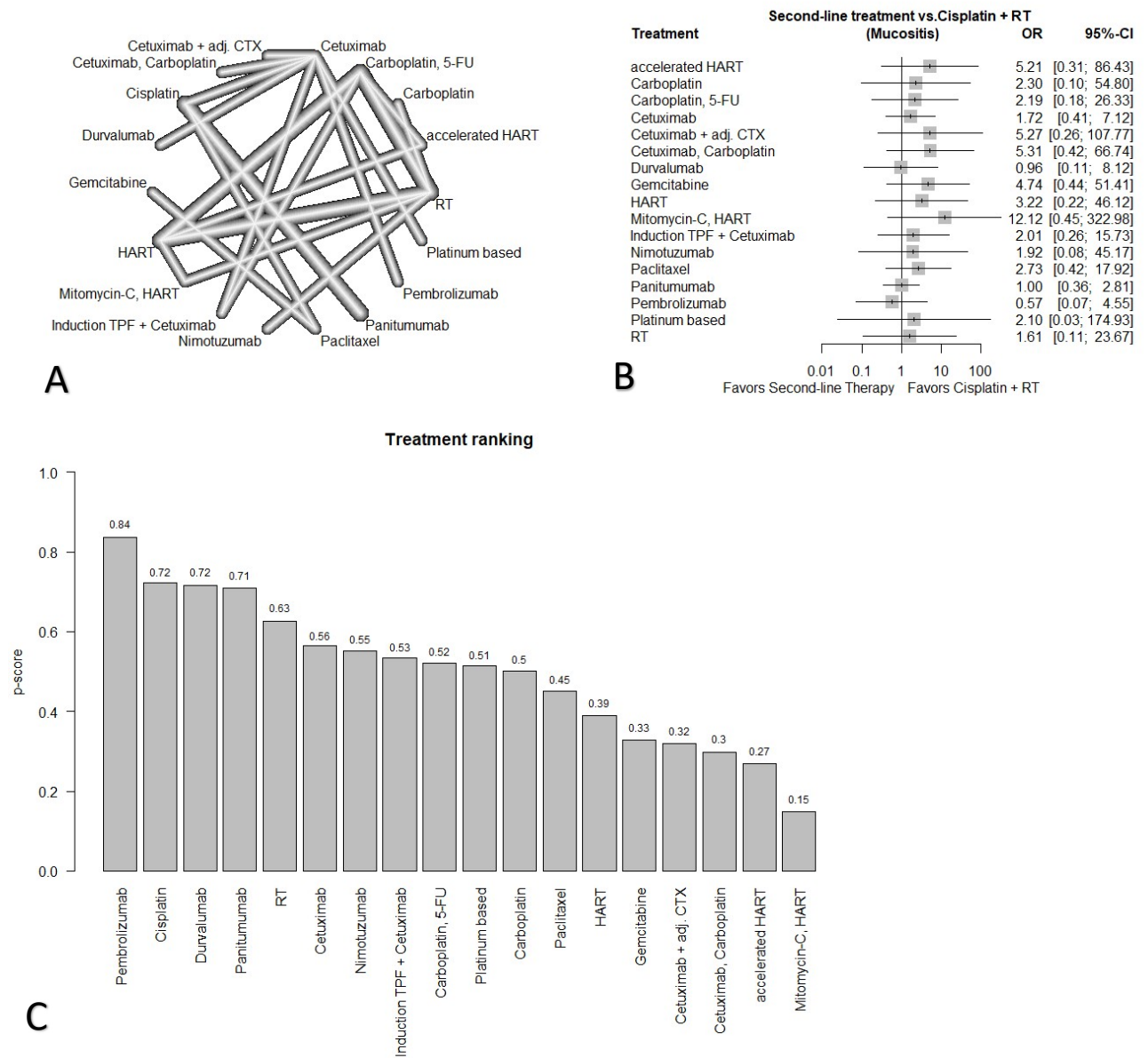

Supplementary material 11: Results for mucositis (A) netgraph, (B) forest plot, (C) treatment ranking

Supplement: Supplementary file 1 [file cancers-18-01599-s001.zip › cancers-4313239-supplementary/Supplementary material 12.pdf]

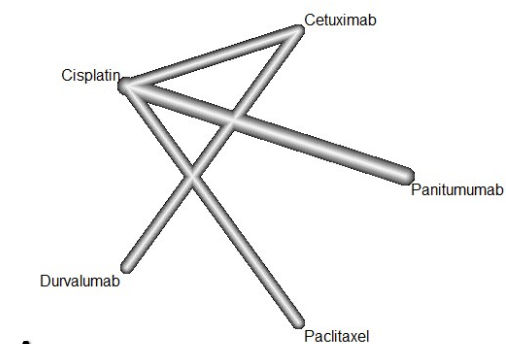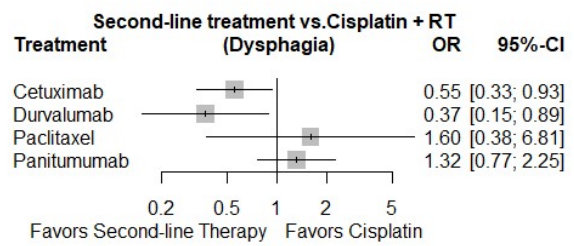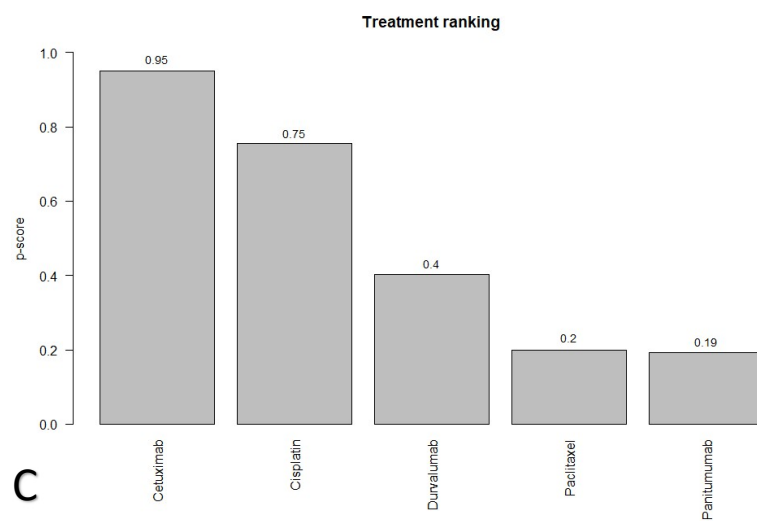

Supplementary material 6: Results for dysphagia (A) netgraph, (B) forest plot, (C) treatment ranking

Supplement: Supplementary file 1 [file cancers-18-01599-s001.zip › cancers-4313239-supplementary/Supplementary material 7.pdf]
